# Bacterial Foreignization Nanosystem Elicits Multi‐Phenotypic T Cells for Antitumor Immunity

**DOI:** 10.1002/advs.202504155

**Published:** 2025-07-10

**Authors:** Wan‐Ru Zhuang, Wen‐Chi Xue, Chao Liang, Pan Liu, Yao Lei, Jiaqi He, Ran Cheng, Weidong Nie, Jianxiong Wang, Jie Tang, Hai‐Yan Xie

**Affiliations:** ^1^ Chemical Biology Center Peking University Beijing 100191 China; ^2^ State Key Laboratory of Natural and Biomimetic Drugs School of Pharmaceutical Sciences Peking University Beijing 100191 China; ^3^ Ningbo Institute of Marine Medicine Ningbo 315832 China; ^4^ School of Medical Technology Beijing Institute of Technology Beijing 100081 China; ^5^ School of Life Science Beijing Institute of Technology Beijing 100081 China; ^6^ Drug Delivery Disposition and Dynamics Monash Institute of Pharmaceutical Sciences Monash University Parkville 3052 Australia

**Keywords:** bacterial outer membrane vesicles, foreignized tumor cells, multi‐phenotypic T cells, tumor intrinsic immunogenicity

## Abstract

T lymphocytes are pivotal targets in clinical cancer immunotherapy; however, tumor cells frequently evade the T‐cell attacks by altering the intrinsic immunogenicity manifested as adjuvanticity, antigenicity, and reactogenicity. Here, a bacterial outer membrane vesicle (OMVs)‐based nanosystem is presented to elicit robust T‐cell responses by reshaping tumor immunogenicity. OMVs are engineered with vesicular stomatitis virus G‐protein that facilitates the fusion of OMVs with tumor cells, leading to tumor “foreignization” and adjuvanticity augment. Consequently, the innate immune system is mobilized to sense the foreignized tumor cells and present the whole‐cell tumor antigens, significantly improving the tumor antigenicity and priming multi‐phenotypic T cells, including both pathogen‐specific and tumor‐specific T cells. Meanwhile, the tumor reactogenicity is reinvigorated through direct cytoplasmic delivery of siPD‐L1 during the fusion, amplifying T cells‐mediated cytotoxicity. In murine models, this foreignization strategy potentiates adaptive immunity and induces durable immune memory, thus effectively suppressing bilateral and metastatic tumors, demonstrating its therapeutic potential in comprehensively reprogramming tumor immunogenicity to fight cancer.

## Graphical abstract

In this study, a comprehensive strategy for tumor immunogenicity reshaping is implemented, in which all adjuvanticity, antigenicity, and reactogenicity of tumor cells are highly improved by an engineered bacterial nanosystem through a selective membrane fusion process. The resulting foreignized tumor cells evoke potent innate immune responses, inducing the spreading of whole‐cell antigens, including bacterial antigens, thereby priming multi‐phenotypic T cells, particularly pathogen‐specific T cells, for effective cancer therapy.

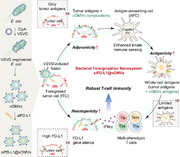

## 1 Introduction

Tumor‐infiltrating lymphocytes (TILs), especially the tumor‐specific T cells (Tts), play crucial roles in immune‐mediated tumor eradication.^[^ ^1^ ^]^ However, accumulating evidence reveals that most TILs are inactive or bystanders, with active cytotoxic Tts being rare.^[^ ^2^ ^]^ This inactivity closely associates with tumor‐induced suppression of immunogenicity, including adjuvanticity, antigenicity, and reactogenicity.^[^ ^3^ ^]^ First of all, tumors tend to reduce the adjuvanticity by subverting immunogenic cell death (ICD), preventing the recognition of tumor cells and the presentation of tumor antigens by the innate immune cells.^[^ ^4^ ^]^ Meanwhile, antigenicity loss is inevitable in most malignant cells through downregulating the tumor‐associated antigens (TAAs) and neoantigens, limiting the generation and diversity of Tts.^[^ ^5^ ^]^ In addition, TAAs‐specific Tts are usually eliminated in the thymus by the central tolerance mechanism.^[^ ^6^ ^]^ Even if some Tts can infiltrate into tumors, the checkpoint molecules, such as PD‐L1, inhibit the tumor reactogenicity, leading to the Tts exhaustion and anergy.^[^ ^7^ ^]^ Therefore, restoring the immunogenicity of tumor cells is emphasized as a closed‐loop approach for T‐cell immunity.

Efforts have been made in recent years to reprogram tumor immunogenicity, but there are still many problems. For example, several strategies are proposed to enhance tumor adjuvanticity by restoring or reinforcing ICD since it is accompanied by the emitting of damage‐associated molecular patterns.^[^ ^8^ ^]^ However, these adjuvant‐like danger signals are exclusive in necrotic tumor cells and mostly unavailable due to their release into the extracellular space.^[^ ^9^ ^]^ Approaches such as DNA mutation,^[^ ^10^ ^]^ aberrant RNA splicing,^[^ ^11^ ^]^ subcellular knockout,^[^ ^12^ ^]^ and cell transdifferentiation^[^ ^13^ ^]^ have been reported to promote tumor antigenicity. However, the neoepitopes generated through mutation or splicing may suffer from deletion, and only a small proportion of them are antigenic enough to elicit T cells.^[^ ^14^ ^]^ Subcellular knockout or transdifferentiation can improve the TAAs presentation by upregulating the expression of major histocompatibility complex (MHC), but Tts specific for TAAs are easily trapped by T‐cell tolerance. To unleash the tumor reactogenicity toward Tts, specific antibodies like anti‐PD‐L1 are used to disrupt the immune checkpoint.^[^ ^15^ ^]^ Nevertheless, only the existing PD‐L1 can be blocked, and antibody resistance is easily developed.^[^ ^16^ ^]^ Therefore, innovative strategies for immunogenicity reprogramming are needed.

The bacteria and bacterial derivatives are of excellent adjuvanticity due to the abundant pathogen‐associated molecular patterns (PAMPs) on the surface.^[^ ^17^ ^]^ Bacterial infection can trigger immediate innate immunity,^[^ ^18^ ^]^ which is also the prerequisite for adaptive T‐cell responses.^[^ ^19^ ^]^ More importantly, the pathogen‐specific T cells (Tps) are the bystanders that can bypass the central immune tolerance.^[^ ^20^ ^]^ These unique and versatile immunoregulatory functions of bacteria have attracted great attention in immunotherapy.^[^ ^21^ ^]^ The bacterial outer membrane vesicles (OMVs) have been used to potentiate cancer vaccination, and several OMVs‐based vaccines have been approved in some countries.^[^ ^22^ ^]^ The tumor‐associated bacteria were targeted to yield microbial epitopes for enhancing tumor antigenicity and priming T cells.^[^ ^23^ ^]^ Although prospective, their potential to directly modulate the immunogenicity of tumor cells remains underexplored.

**Figure 1:**
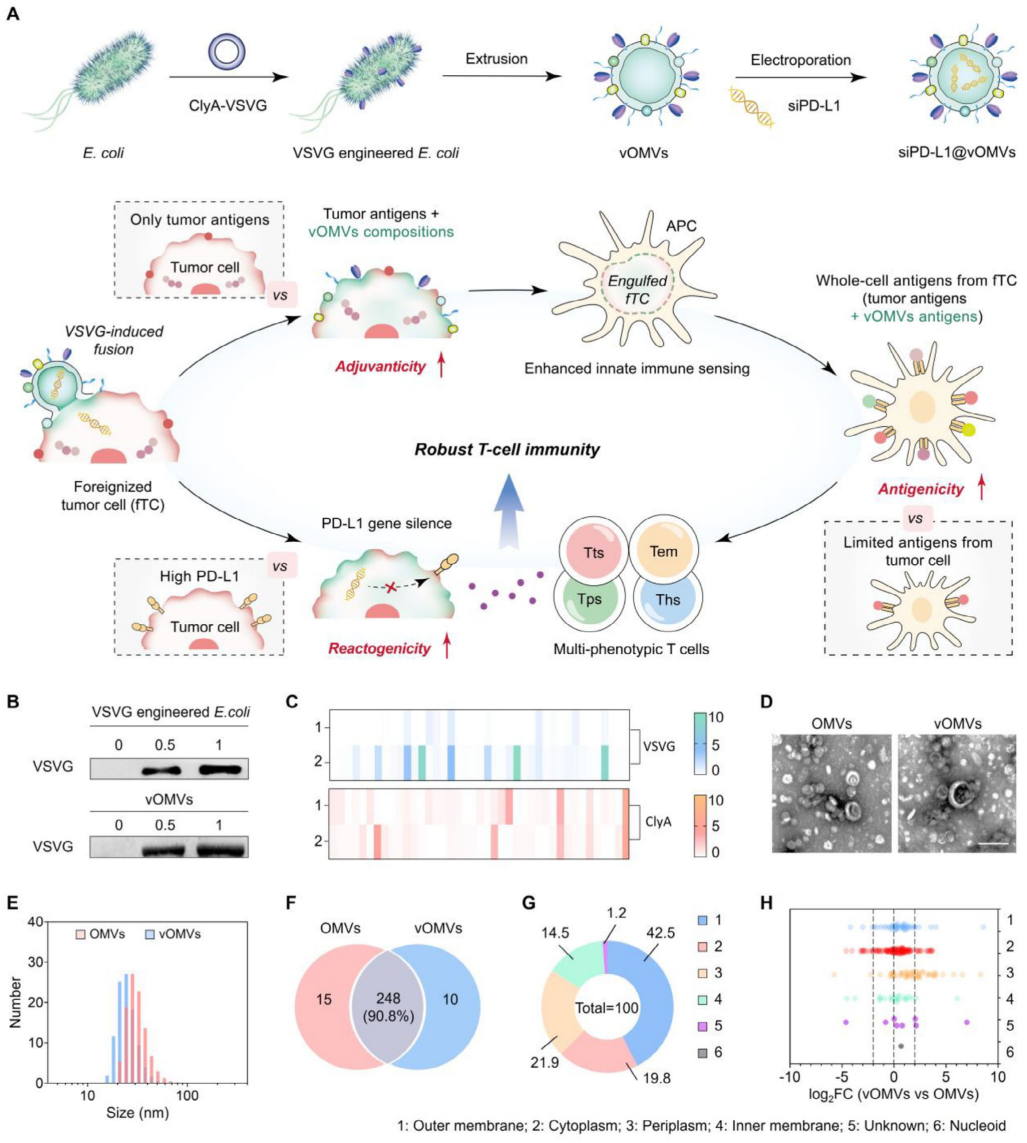
Therapeutic illustration and characterization of siPD‐L1@vOMVs. A) Therapeutic strategy of siPD‐L1@vOMVs for tumor foreignization. B) Western blot analysis of VSVG in engineered *E. coli* and the derived vOMVs (IPTG concentration: 0, 0.5, 1 mM). C) Heatmap of the expression of the recombinant ClyA‐VSVG peptides verified by proteomic analysis (1: OMVs; 2: vOMVs). D) TEM images. Scale bar: 100 nm. E) Size distribution. F) Venn diagram of identified proteins from OMVs and vOMVs by proteomic analysis. G) Pie chart for the subcellular localization of expressed proteins in vOMVs. H) Fold change (FC) of common proteins calculated by the ratio between protein levels of vOMVs and OMVs.

Herein, we develop siPD‐L1@vOMVs, a nanosystem designed for the comprehensive tumor immunogenicity reshaping, thus the priming and boosting of multi‐phenotypic T cells (**Figure** **1**A). The OMVs are engineered with vesicular stomatitis virus G‐protein (VSVG), which enables the selective fusion of OMVs with tumor cells in an acid‐sensitive and lipoprotein receptor‐dependent manner. This fusion results in the foreignized tumor cells (fTCs), significantly enhancing adjuvanticity, and effectively mobilizing the innate immune cells to sense fTCs. The subsequent presentation of the whole‐cell tumor antigens significantly enhances the tumor antigenicity and expands T‐cell phenotypes to include Tts and pathogen‐specific T cells (Tps). Meanwhile, siPD‐L1 is loaded into OMVs, which are directly delivered to the cytoplasm during the membrane fusion, silencing PD‐L1 and restoring the reactogenicity of tumor cells to T cells. Moreover, the helper T cells (Ths) and effector memory T cells (Tem) are potently stimulated, synergizing with the effector Tts to generate robust T‐cell immunity and durable immune memory. As a result, both bilateral and metastatic tumors are effectively suppressed, providing a pioneering concept for T cell‐based antitumor immunotherapy.

## 2 Results and Discussion

### 2.1 Preparation and Characterization of vOMVs

To engineer OMVs of *Escherichia coli* (*E. coli*) with VSVG, the sequence of VSVG was fused with the C‐terminus of proteinaceous cytolysin A (ClyA), which is specifically embedded in the bacterial outer membrane.^[^ ^24^ ^]^ The expression of recombinant protein ClyA‐VSVG on E. coli and the derived OMVs was verified by SDS‐PAGE, western blot, and mass spectrometry analyses (Figure 1B,C; Figure S1, Supporting Information). Both OMVs and VSVG‐fused OMVs (vOMVs) exhibited uniform size distribution with the average size ≈20 nm, which was kept during one‐week storage (Figure 1D,E; Figure S2, Supporting Information). The total protein analysis found that the compositions of OMVs and vOMVs were of no significant difference since 90.8% of the expressed proteins were common (Figure 1F). Meanwhile, more than 80% of proteins in vOMVs were located at the outer membrane, periplasm, or cytoplasm, consistent with that in OMVs (Figure 1G; Figure S3, and Table S1, Supporting Information). Although most of the identified proteins were moderately regulated or even unchanged in subcellular localization (defined as |log2FC|<2), a certain increase of periplasmic proteins in vOMVs was found (defined as |log2FC|≥2) (Figure 1H). This was reasonable since some enzymes are necessary for the recombinant protein folding in the periplasm.^[^ ^25^ ^]^ All these results confirmed the successful engineering of OMVs with VSVG, while the inherent features of OMVs were retained in vOMVs.

### 2.2 Tumor Foreignization Triggering Tumor Internalization and Immune Activation

**Figure 2:**
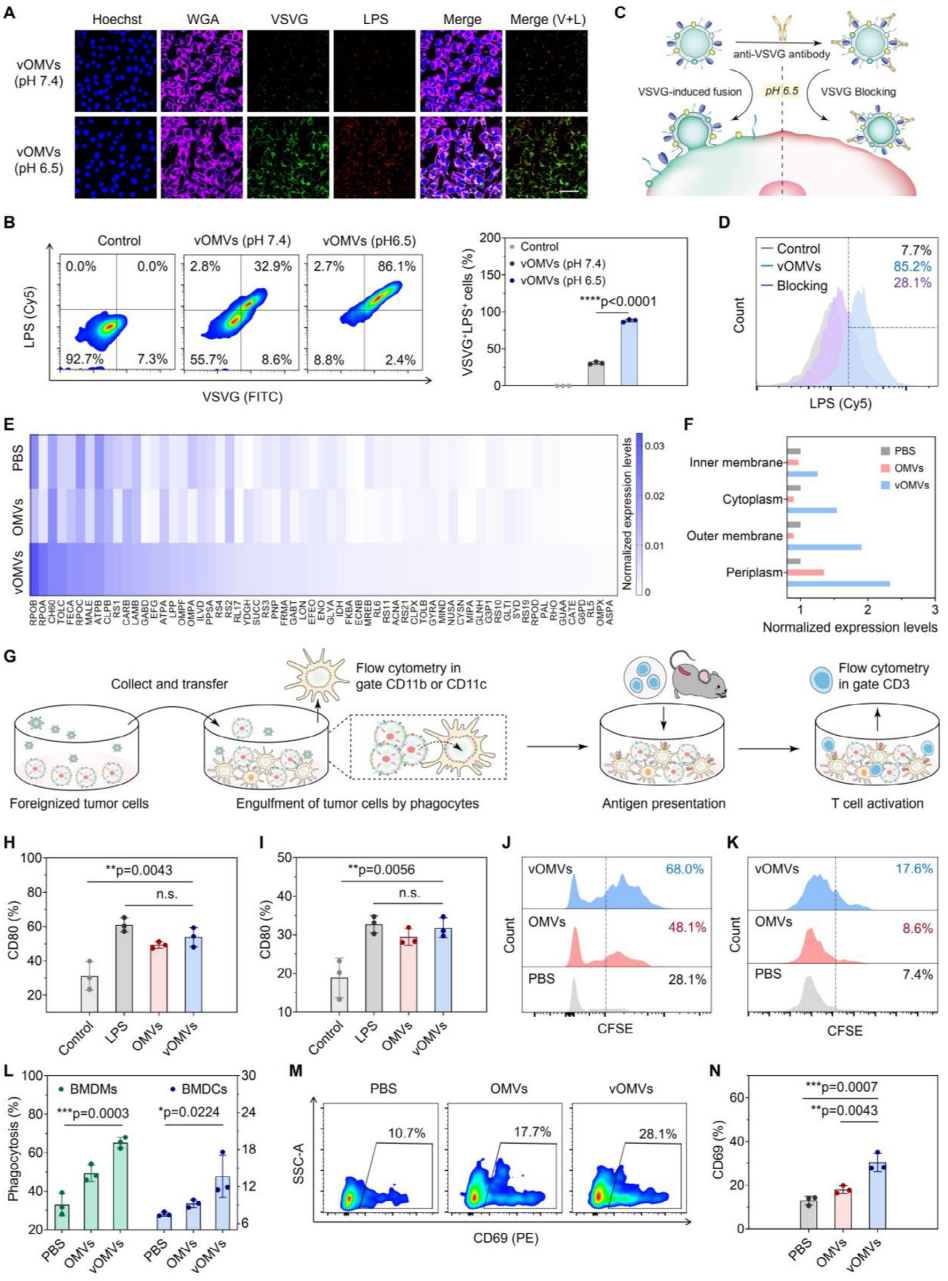
In vitro tumor foreignization ability and immunoregulatory effect of vOMVs. A) CLSM imaging of the vOMVs‐fused tumor cells. CT26 cells were separately incubated with OMVs or vOMVs in the medium with pH 7.4 or pH 6.5, and the presence of VSVG and LPS on the cell membrane was measured by CLSM. Cell membrane (iFluor555‐conjugated WGA labeled, pink); VSVG (FITC‐conjugated anti‐VSVG labeled, green); LPS (Cy5‐conjugated anti‐LPS labeled, red); Cell nucleus (Hoechst 33342, blue). Scale bar: 50 µm. B) Flow cytometry and quantification of VSVG^+^LPS^+^ tumor cells after different treatments. C) Schematic of vOMVs‐mediated membrane fusion and the inhibition of fusion by the antibody blockade. D) Flow cytometry of LPS^+^ tumor cells after different treatments. E) Heatmap of bacterial proteins on the tumor cell surface detected by proteomic analysis. F) Subcellular localization of fused proteins identified in (E). G) Schematic illustration of the experiment of immune cell activation. Foreignized tumor cells (fTCs) along with the supernatant were co‐cultured with BMDMs or BMDCs for 4 h, and the cells from different treatments were collected for flow cytometry assay in the gate CD11b or CD11c. Then, mouse splenic T cells were isolated and incubated with the above mixed cell cultures, and the cell suspension containing T cells was collected for flow cytometry assay in the gate CD3. H,I) Expression levels of CD80 on BMDMs and BMDCs were analyzed by flow cytometry. LPS (1 µg mL^−1^) is a positive control. J,K) Flow cytometry of the phagocytosis of fTCs by BMDMs and BMDCs. The CFSE‐labeled CT26 cells were pretreated with vOMVs in pH 6.5 medium. Next, fTCs along with the supernatant were collected to incubate with BMDMs or BMDCs. The phagocytosis of fTCs by APCs (vOMVs group) was evaluated by comparison with native CT26 tumor cells (control group) or OMVs‐treated tumor cells (OMVs group). L) Quantification of the phagocytosis of fTCs. M,N) Flow cytometry and quantification of expression levels of CD69 gated on CD8^+^ T cells. Data in B, D, H, I, L, and N are presented as mean ± s.d. (n = 3 biologically independent samples). Statistically significant differences between groups were identified by an unpaired two‐tailed Student's t‐test. ^****^ *P* < 0.0001, ^****^ *P* < 0.001, ^**^ *P* < 0.01, ^*^ *P* < 0.05, n.s., not significant.

VSVG is a typical type‐III fusion protein that can be activated via conformational transition in acidic conditions (pH ≈5 to 6), enabling the virus attachment and invasion of the host cell.^[^ ^26^ ^]^ To evaluate the pH‐sensitive fusion ability of vOMVs, we incubated OMVs or vOMVs with CT26 tumor cells in the medium of pH 7.4 or 6.5. The subsequent confocal laser scanning microscope (CLSM) imaging showed that neither VSVG nor lipopolysaccharides (LPS) were found on the cell surface after OMVs treatment in both pH 7.4 and 6.5 (Figure S4, Supporting Information). There were also no obvious signals on the cells of the vOMVs (pH 7.4) group (**Figure** **2**A). In contrast, strong signals of VSVG and LPS appeared in the vOMVs (pH 6.5) group, and the green fluorescence of VSVG overlapped with the red signal from LPS (as shown in “V+L” channel). The proportion of VSVG^+^LPS^+^ CT26 cells reached almost 90% as determined by flow cytometry (Figure 2B; Figure S5A, Supporting Information). Moreover, the VSVG proteins transferred from vOMVs could stay for at least 6 h on CT26 cells (Figure S5B, Supporting Information). But once vOMVs were pre‐blocked by excessive anti‐VSVG antibodies, the percentage of LPS^+^ tumor cells decreased to 28% (Figure 2C,D; Figure S6, Supporting Information). The proteomic analysis found a total of 63 bacterial proteins on the cell membrane after vOMVs treatment at pH 6.5 (Figure 2E; Figure S7, Supporting Information), including the bacterial periplasmic proteins, outer membrane proteins, and cytoplasmic proteins, nearly all from vOMVs (Figure 2F; Table S2, Supporting Information). All these results proved the acid‐dependent fusion of vOMVs with tumor cells, resulting in the foreignization of tumor cells, whose adjuvanticity would be enhanced.

Antigen‐presenting cells (APCs), mainly including macrophages and DCs, are the “sentinels” of the innate immune system,^[^ ^27^ ^]^ and OMVs are strong activators of pattern recognition receptors (PRRs) expressed on APCs.^[^ ^28^ ^]^ Therefore, OMVs as well as vOMVs could effectively activate the bone marrow‐derived macrophages (BMDMs) and bone marrow‐derived DCs (BMDCs), demonstrated by the significant upregulation of polarization or maturation markers, such as CD80, CD86, and MHC‐II (Figure 2H,I; Figures S8 and S9, Supporting Information). In general, tumor cells with low mutation loads usually lack immunogenicity and cannot be efficiently recognized by APCs.^[^ ^29^ ^]^ As anticipated, CT26 cells without treatment could hardly be phagocytized by BMDMs or BMDCs. Notably, the internalization of vOMVs‐foreignized tumor cells (fTCs) was distinctly augmented due to the innate sensing ability of APCs toward the bacterial compositions (Figure 2J–L), which would lead to the effective presentation of whole‐cell antigenic peptides and then the stimulation of T cells. As expected, the splenic T cells were effectively activated after 24 h incubation with the mixture of BMDCs and fTCs, as proved by the significantly increased expression of CD69 (Figure 2G,M,N; Figure S10, Supporting Information).

### 2.3 In Vivo Modulation of Innate Immunity Broadening the T Cell Epitopes

**Figure 3:**
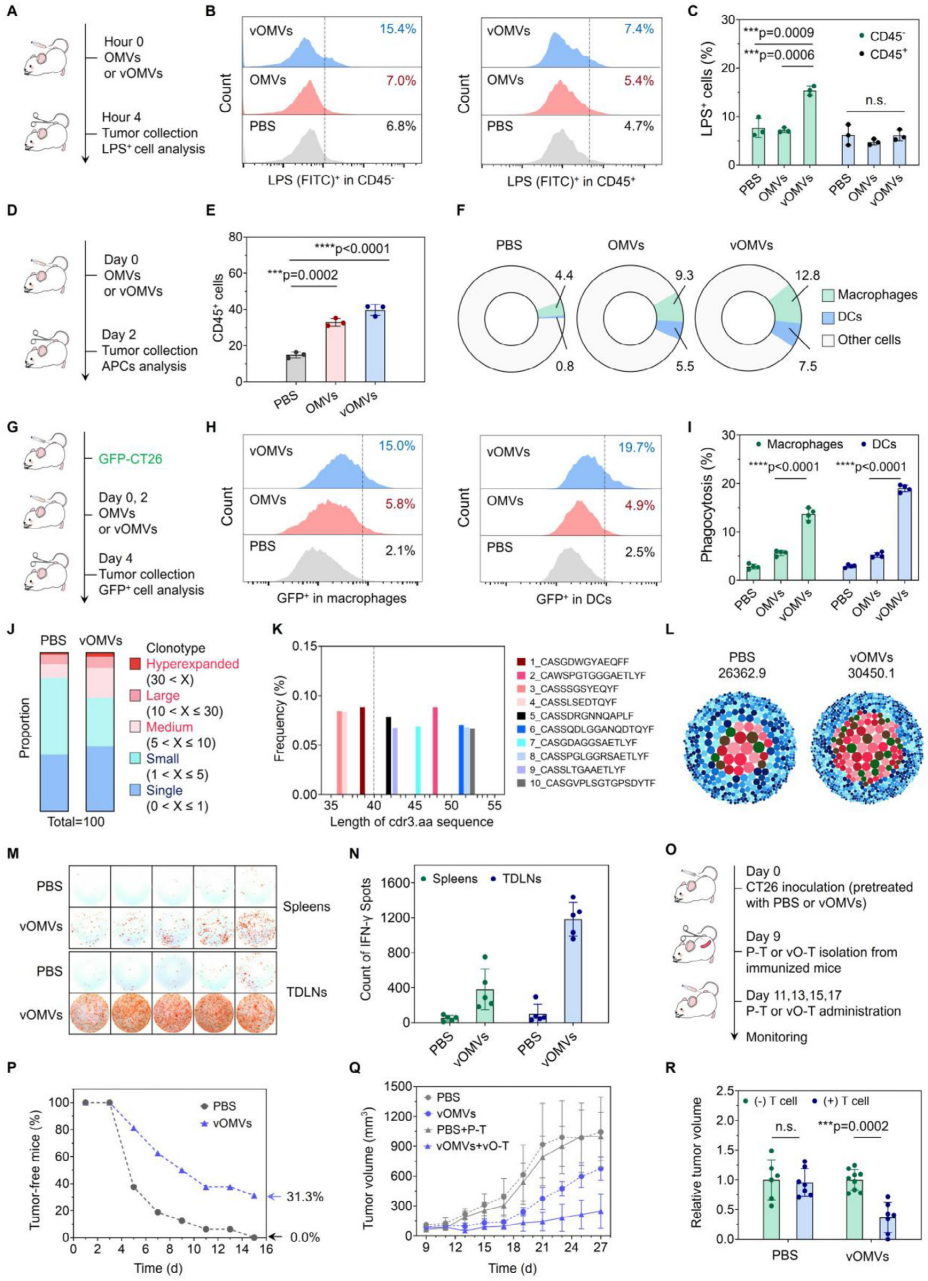
Innate immunity induced by vOMVs broadens the phenotypic diversity of T cells. A) Experimental design for detecting tumor foreignization. CT26 tumor‐bearing mice were intratumorally injected with PBS, OMVs, or vOMVs, and the tumors were extracted at 4 h post‐administration. The LPS^+^ cells were then detected by flow cytometry. B,C) Flow cytometry and quantification of LPS^+^ tumor cells (gated on CD45^+^ or CD45^‐^ cells). D) Experimental design for detecting the APCs recruitment. CT26 tumor‐bearing mice were intratumorally injected with PBS, OMVs, or vOMVs, and the tumors were extracted at 48 h post‐administration. The infiltrating immune cells were then detected by flow cytometry. E) Quantification of CD45^+^ immune cells in tumors. F) Pie chart for the proportions of macrophages and DCs in tumor tissues. G) Experimental design for detecting the phagocytosis of fTCs in vivo. GFP‐CT26 tumor‐bearing mice were intratumorally injected with PBS, OMVs, or vOMVs, and the tumors were extracted at 48 h post‐administration. The GFP^+^ cells in tumors were then detected by flow cytometry. H,I) Flow cytometry and quantification of GFP^+^ cells (gated on F4/80^+^ or CD11c^+^ cells) in tumors. J) Frequencies of TCR clonotypes of T cells in TDLNs from PBS or vOMVs‐treated mice. These TCR clonotypes were divided into five groups, including hyperexpanded (30 < X), large (10 < X ≤ 30), medium (5 < X ≤ 10), small (1 < X ≤ 5), and single (0 < X ≤ 1). K) Cdr3 length distribution. L) Bubble charts showing TCR diversity (Inverse Simpson index, data from single clonotypes were not shown). M) Representative images of IFN‐γ ELISpot (Up: splenocytes; Down: TDLN cells). N) Quantification of IFN‐γ spots. O) Experimental design for P‐R. P) Tumorigenesis of CT26 cells pretreated with PBS or vOMVs. Q) Antitumor effect of adoptive splenic T cells obtained from vOMVs‐immunized mice (vO‐T) and PBS‐treated mice (P‐T). R) Relative tumor volume after adoptive T therapy. Data in C, E, and I are presented as mean ± s.d. (n = 3 biologically independent mice). Data in N are presented as mean ± s.d. (n = 5 biologically independent mice). Data in P‐R are presented as mean ± s.d. (n = 16 biologically independent mice, mice in Q,R were randomly divided from mice in p). Statistically significant differences between groups were identified by an unpaired two‐tailed Student's *t*‐test. ^*****^ *P* < 0.0001, ^***^ *P* < 0.001, ^**^ *P* < 0.01, ^*^ *P* < 0.05, n.s., not significant.

Next, we explored the capability of vOMVs in restoring innate immunity in vivo. First, it was found that the proportion of fTCs (defined as LPS^+^CD45^‐^ cells or VSVG^+^CD45^‐^ cells) in vOMVs treated tumors was ≈2‐fold higher than that in the OMVs group, suggesting a highly increased intrinsic immunogenicity of tumor cells (**Figure** **3**A–C; Figure S11, Supporting Information). We noted that LPS or VSVG signals were hardly observed in immune cells (defined as CD45^+^ cells), probably because of the low expression of lipoprotein receptors that were identified as a primary receptor facilitating VSVG fusion with the tumor cell membrane,^[^ ^30^ ^]^ highlighting the fusion specificity of vOMVs. Meanwhile, due to the outstanding adjuvanticity of OMVs for immune cell recruitment,^[^ ^31^ ^]^ the tumor‐infiltrating CD45^+^ immune cells, F4/80^+^ macrophages, and CD11c^+^ DCs were all remarkably elevated in the OMVs and vOMVs group (Figure 3D–F; Figure S12, Supporting Information). To assess whether fTCs could be phagocytized by the recruited APCs, green fluorescent protein (GFP)‐expressing CT26 cells were used, and the GFP signals in macrophages or DCs were detected 48 h post‐administration (Figure 3G). As could be seen, the GFP^+^ macrophages and DCs in the vOMVs group were more than 7‐fold higher than those in the PBS control (Figure 3H,I; Figure S13, Supporting Information), again validating that fTCs contained strong sensing and phagocytic signals for the innate immune cells.

The augmented phagocytosis effects would improve the in situ antigen utilization and presentation. For verification, we evaluated the T cell receptor (TCR) repertoire of T cells in tumor‐draining lymph nodes (TDLNs) by TCR sequencing analysis. After vOMVs treatment, enhanced oligoclonal expansion of T cells was observed, and the frequencies of highly expanded clonotypes (X>5) increased by 12.6% in comparison to PBS control (Figure 3J), suggesting more active T cell epitopes. It was worth mentioning that among the top 10 representative complementarity‐determining region 3 (cdr3) sequences, T cells from vOMVs‐immunized mice showed more variation in cdr3 length distribution (Figure 3K; Figure S14, Supporting Information), indicating the diverse T cell epitopes for recognizing the processed antigen peptides.^[^ ^32^ ^]^ Depth TCR repertoire analysis also confirmed that T cells from the vOMVs group exhibited TCR clones with greater breadth and diversity (Figure 3L; Table S3, Supporting Information), corroborating that the foreignization‐promoted phagocytosis led to more efficient epitope spreading of whole‐cell tumor antigens and thus the considerable priming of multi‐phenotypic T cells.

Besides tumor‐specific antigens, APCs can also present bacteria‐associated antigens to T cells, leading to the activation and proliferation of pathogen‐specific T cells (Tps). This is of great importance since Tps are usually hardly exhausted, free from tolerance, and even preserve memory properties for T cell receptors (TCR)‐independent activation.^[^ ^33^ ^]^ Therefore, we particularly detected the Tps after restimulating splenocytes or TDLN cells with vOMVs peptides. The enzyme‐linked immunospot (ELISpot) assay results showed that T cells from vOMVs‐immunized mice (vO‐T) produced more cytotoxic interferon‐gamma (IFN‐γ) in comparison with those from the PBS (P‐T) group (Figure 3M,N), indicating the presence of Tps. To explore the efficacy of activated Tps against tumors, CT26 cells were pretreated with PBS or vOMVs, and then injected into mouse subcutaneously (Figure 3O). It was found that the tumorigenesis of CT26 cells was retarded in the vOMVs group, which should be ascribed to the foreignization of tumor cells with vOMVs and then the improved tumor adjuvanticity (Figure 3P). Next, the appeared tumors in PBS or vOMVs group were adoptively treated with P‐T or vO‐T, which were pre‐stimulated by vOMVs peptides. As expected, P‐T exhibited slight therapeutic effects, whereas vO‐T effectively inhibited tumor growth (Figure 3Q,R). Taken together, the vOMVs‐based tumor foreignization strategy could spread bacterial epitopes along with tumor‐specific epitopes, thereby broadening the phenotypic diversity of T cells. Given that most TAAs‐specific T cells are usually eliminated in the thymus, these multi‐phenotypic T cells possibly bypass the central immune tolerance.

### 2.4 PD‐L1 Gene Silence Reinvigorating the Tumor Reactogenicity Toward T cells

**Figure 4:**
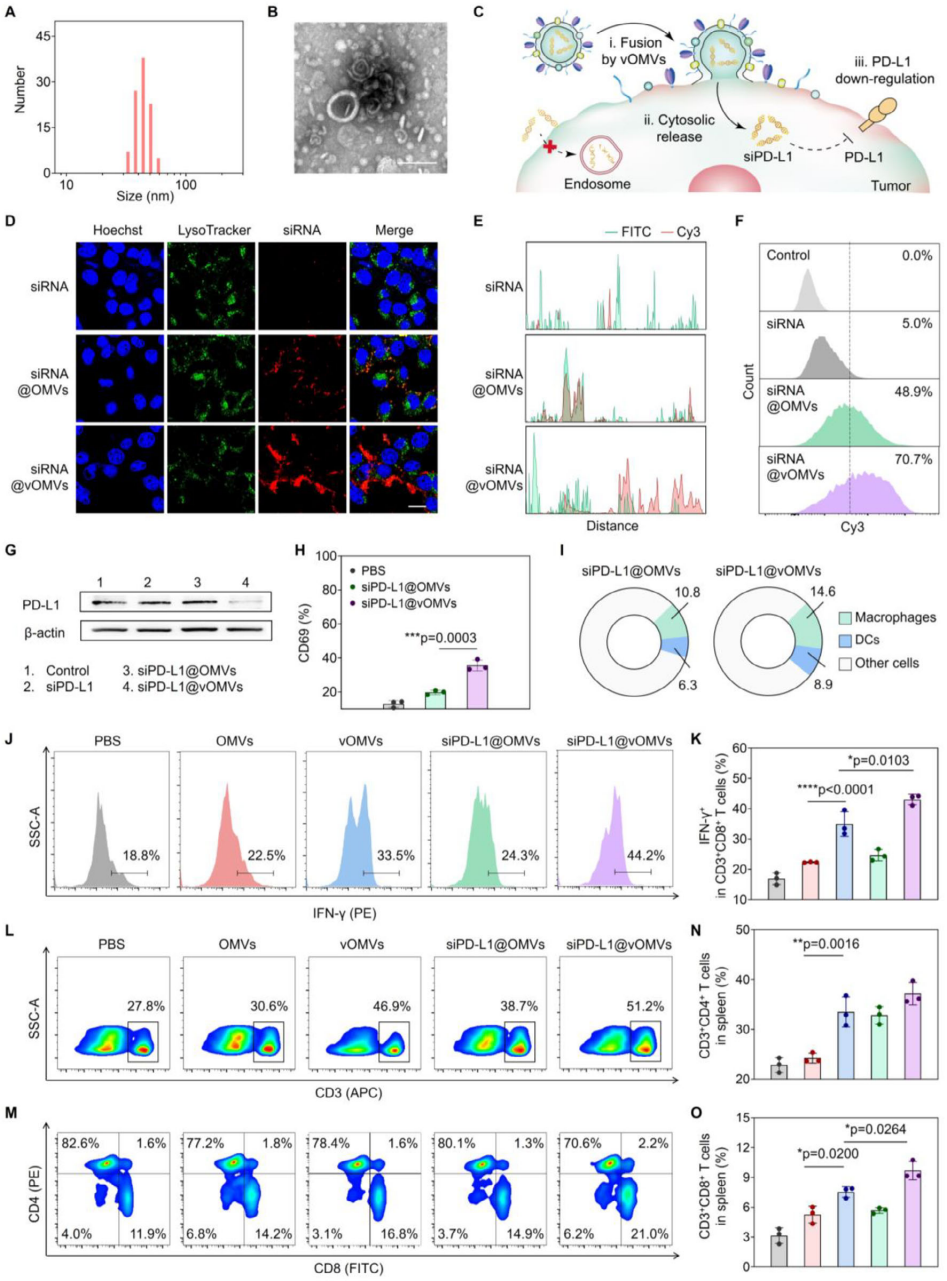
Enhanced tumor reactogenicity toward T cells by siPD‐L1@vOMVs. A) Size distribution. B) TEM image. Scale bar: 100 nm. C) Schematic illustration of vOMVs‐promoted cytoplasmic delivery of siRNA and PD‐L1 gene silence. D) CLSM imaging of siRNA internalization. The CT26 tumor cells were individually incubated with Cy3‐labeled siRNA, siRNA‐loaded OMVs (siRNA@OMVs), and siRNA‐loaded vOMVs (siRNA@vOMVs), and then the internalization of siRNA was analyzed by CLSM. Endosome/lysosome (Lysotracker Green labeled, green); siRNA (Cy3 labeled, red); Cell nucleus (Hoechst 33342, blue). Scale bar: 20 µm. E) Spectrum profiles of the co‐localization of endosome/lysosome and siRNA in (D). F) Flow cytometry of the internalization of siRNA after different treatments. G) PD‐L1 protein expression in CT26 cells after different treatments was determined by western blot. H) Flow cytometry and quantification of expression levels of CD69 gated on CD3^+^CD8^+^ T cells after different treatments. To detect the systemic immune responses in vivo, CT26 tumor‐bearing BALB/c mice were intratumorally injected with PBS, OMVs, vOMVs, siPD‐L1@OMVs, or siPD‐L1@vOMVs, and then the tumors, TDLNs, and spleens were extracted at 48 h post‐administration. I) Pie chart for the proportions of macrophages and DCs in the tumor. J,K) Flow cytometry and quantification of expression levels of IFN‐γ^+^ T lymphocytes (gated on CD3^+^CD8^+^ cells) in tumors. L) Representative flow cytometry plots showing the CD3^+^ T cells in the spleens. M‐O) Percentages of CD3^+^CD4^+^ or CD3^+^CD8^+^ T lymphocytes detected by flow cytometry in the spleens. Data in H, K, N, and O are presented as mean ± s.d. (n = 3 biologically independent samples). Statistically significant differences between groups were identified by an unpaired two‐tailed Student's t‐test. ^****^ *P* < 0.0001, ^***^ *P* < 0.001, ^**^ *P* < 0.01, ^*^ *P* < 0.05, n.s., not significant.

In addition to the deletion by central tolerance, T cells can also be hampered by peripheral tolerance checkpoints.^[^ ^34^ ^]^ To enhance the tumor reactogenicity and avoid peripheral T‐cell tolerance, siPD‐L1 was loaded in vOMVs and delivered to tumor cells in this study. The loading of siRNA was saturated at the ratio of 1:2, with a loading efficiency (LE) of 28.6% and an encapsulation efficiency (EE) of 18.8% (Figure S15, Supporting Information). The siRNA‐loaded vOMVs (siRNA@vOMVs) displayed a similar vesicle structure to that of native OMVs, with a uniform size of 53.1 ± 6.7 nm (**Figure** **4**A,B; Figure S16A–C, Supporting Information). Furthermore, the encapsulated siRNA was stable in vOMVs during storage at 4 °C for 24 h (Figure S16D, Supporting Information).

Endosomal entrapment of siRNA is a ubiquitous problem for most delivery strategies and severely limits the therapeutic outcomes of siRNA.^[^ ^35^ ^]^ Considering the fact that viral genes can be released to the host cell through membrane fusion,^[^ ^36^ ^]^ vOMVs were also expected to avoid the endosomal entrapment of the loaded siRNA (Figure 4C). We found that free siRNA could hardly enter the tumor cells due to the poor membrane permeability.^[^ ^37^ ^]^ OMVs could improve the intracellular siRNA (siRNA@OMVs), but the majority of siRNA were colocalized with endosomes, probably because of the endocytosis of OMVs (Figure 4D,E). Thus, the MFI of siRNA in cells was only moderately improved owing to the fast degradation of siRNA in acidic endosomes (Figure 4F; Figure S17, Supporting Information). When encapsulated into vOMVs, considerable siRNA entered the tumor cells, with 2.5‐fold higher MFI relative to the siRNA@OMVs group. More importantly, most of the siRNA was dispersed in the cytoplasm, and the colocalization ratio between siRNA and endosome decreased by 62.3%, illustrating the direct cytosolic transport of siRNA facilitated by the membrane fusion (Figure S18, Supporting Information). As a result, the PD‐L1 expression in tumor cells was greatly inhibited after siPD‐L1@vOMVs treatment (Figure 4G), and the percentage of CD69^+^ T cells was significantly higher than that of the siPD‐L1@OMVs group (Figure 4H; Figure S19, Supporting Information). Collectively, siPD‐L1@vOMVs could efficiently downregulate the checkpoint PD‐L1 and reinvigorate the reactogenicity of tumor cells toward T cells.

On the other hand, similar to vOMVs, siPD‐L1@vOMVs treatment enhanced the infiltration of macrophages and DCs in tumors (Figure 4I; Figure S20, Supporting Information). As a result, the percentage of intratumoral IFN‐γ^+^ CD8^+^ T cells significantly increased in the siPD‐L1@vOMVs treated mice (Figure 4J,K; Figure S21, Supporting Information). We also evaluated the systemic immune responses in TDLNs and spleens. OMVs or siPD‐L1@OMVs treatment increased the proportion of mature DCs to a certain extent, owing to the adjuvanticity of OMVs (Figures S22 and S23, Supporting Information). Assisted by the tumor foreignization and checkpoint silencing effects, vOMVs and siPD‐L1@vOMVs exhibited much stronger potency in promoting DCs maturation. In consequence, siPD‐L1@vOMVs induced the highest contents of CD3^+^ T cells, CD3^+^CD4^+^ T cells, and CD3^+^CD8^+^ T cells in the spleens, individually increased by 22.6%, 14.3%, and 6.5% in comparison with the PBS control (Figure 4L–O; Figure S24, Supporting Information), confirming the robust elicitation of T cell responses in vivo.

### 2.5 Therapeutic Efficacy in Bilateral Tumor Model

**Figure 5:**
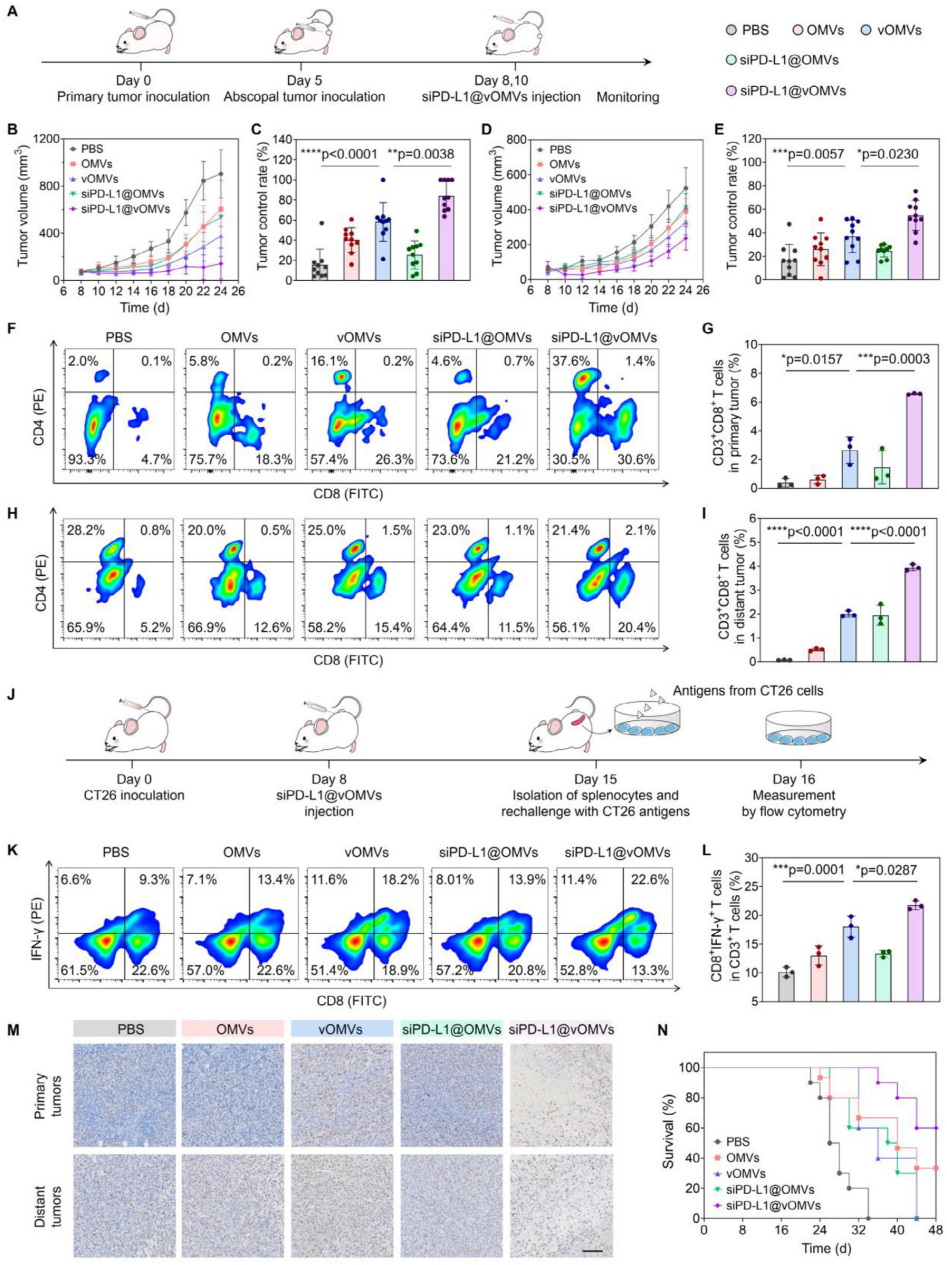
Systemic therapeutic benefit in a bilateral tumor model. A) Schematic illustration of siPD‐L1@vOMVs‐mediated antitumor experiment in a bilateral tumor model. CT26 cells were respectively implanted into the right (as the primary tumors) and left (as the abscopal tumors) flanks of BALB/c mice on days 0 and 5, and when the right tumors reached ≈100 mm^3^, the mice were randomly divided into five groups and intratumorally treated with PBS, OMVs, vOMVs, siPD‐L1@OMVs or siPD‐L1@vOMVs at the primary tumors. The individual tumor volumes of primary and abscopal tumors were recorded every 2 days. B,D) Tumor volumes of the primary tumors and abscopal tumors. C,E) Tumor control rate of the primary tumors and abscopal tumors. F) Representative flow cytometry plots showing the CD8^+^ T cells in primary tumors (gated on CD3^+^ T cells). G) Percentages of CD3^+^CD8^+^ T cell in primary tumors. H) Representative flow cytometry plots showing the CD8^+^ T cells in abscopal tumors (gated on CD3^+^ T cells). I) Percentages of CD3^+^CD8^+^ T cell in abscopal tumors. J) Schematic illustration of the experiment for the determination of antigen‐specific immune responses in splenocytes. The splenocytes from mice with different treatments were collected and re‐stimulated ex vivo with CT26 antigens for 24 h, and then the expression of IFN‐γ^+^CD8^+^ T cells was measured by flow cytometry. K,L) Representative flow cytometry plots and levels of IFN‐γ secretion by splenocytes. M) Ki67 staining of primary and abscopal tumors at the end of the experiment. Scale bar: 100 µm. N) Survival curve of each group. Data in b‐e and n are presented as mean ± s.d. (n = 9 biologically independent mice). Data in G, I, and L are presented as mean ± s.d. (n = 3 biologically independent mice). Statistically significant differences between groups were identified by an unpaired two‐tailed Student's t‐test. ^****^ *P* < 0.0001, ^***^ *P* < 0.001, ^**^ *P* < 0.01, ^*^ *P* < 0.05, n.s., not significant.

Encouraged by the systemic and potent T cell‐based immunity provoked by siPD‐L1@vOMVs, we next evaluated its antitumor efficacy in a bilateral tumor model (**Figure** **5**A). OMVs exhibited faint therapeutic effects on distant tumors, and the tumor control rate was similar to that of the PBS group (Figure 5B–E; Figure S25, Supporting Information), illustrating the insufficient immune responses induced by OMVs. siPD‐L1@OMVs did not display a more pronounced effect in suppressing either primary or distant tumors owing to the poor siRNA delivery efficiency of OMVs. Conversely, vOMVs treatment not only inhibited the primary tumors but also notably delayed the growth of distal tumors. Upon assistance by siPD‐L1 (siPD‐L1@vOMVs), the suppression of bilateral tumors was further improved, with the tumor inhibitory rate reaching ≈85% in primary tumors and 55% in abscopal tumors.

We next investigated the immunophenotyping of T lymphocytes isolated from the bilateral tumors of different groups. Consistently, siPD‐L1@vOMVs treatment displayed the highest activation of tumor‐infiltrating CD8^+^ and CD4^+^ T cells in both primary and distant tumors (Figure 5F,H; Figure S26, Supporting Information). The percentage of CD8^+^ T cells in distant tumors was 56.1 times that of the PBS group (Figure 5G,I). Particularly, the proportion of CD4^+^ T cells increased by 16.2‐fold (Figure S27, Supporting Information), which would augment the tumor‐killing capacity of CD8^+^ T cells.^[^ ^38^ ^]^ In addition, large amounts of IFN‐γ were produced by the splenocytes from siPD‐L1@vOMVs treated mice, confirming the abundant cytotoxic tumor‐specific T cells (Tts) in vivo (Figure 5J–L; Figure S28, Supporting Information). These activated Tts were consistent with the reshaped tumor immunogenicity by our foreignization strategy, thus generating the whole‐antigen dependent priming and boosting of cytotoxic Tts. Therefore, extensive tumor damage and decreased proliferation were found in the bilateral tissues, further proving the strong curative effects of siPD‐L1@vOMVs (Figure 5M; Figure S29, Supporting Information). Thus, improved survival was achieved as the mice in other groups died within 40 days, but ≈60% of the mice survived after siPD‐L1@vOMVs therapy (Figure 5N). We also monitored the body weight of mice as a proxy for mouse health and observed no significant loss in mice treated with siPD‐L1@vOMVs (Figure S30, Supporting Information). Moreover, the H&E staining of the heart, liver, spleen, lung, and kidney showed negligible tissue damage (Figure S31, Supporting Information). The satisfactory safety profile of the siPD‐L1@vOMVs‐based therapy was also confirmed by the normal levels of hepatic, cardiac, or renal function biomarkers (Figure S32, Supporting Information).

### 2.6 Inhibition Efficacy of siPD‐L1@vOMVs in Metastatic Tumor Model

**Figure 6:**
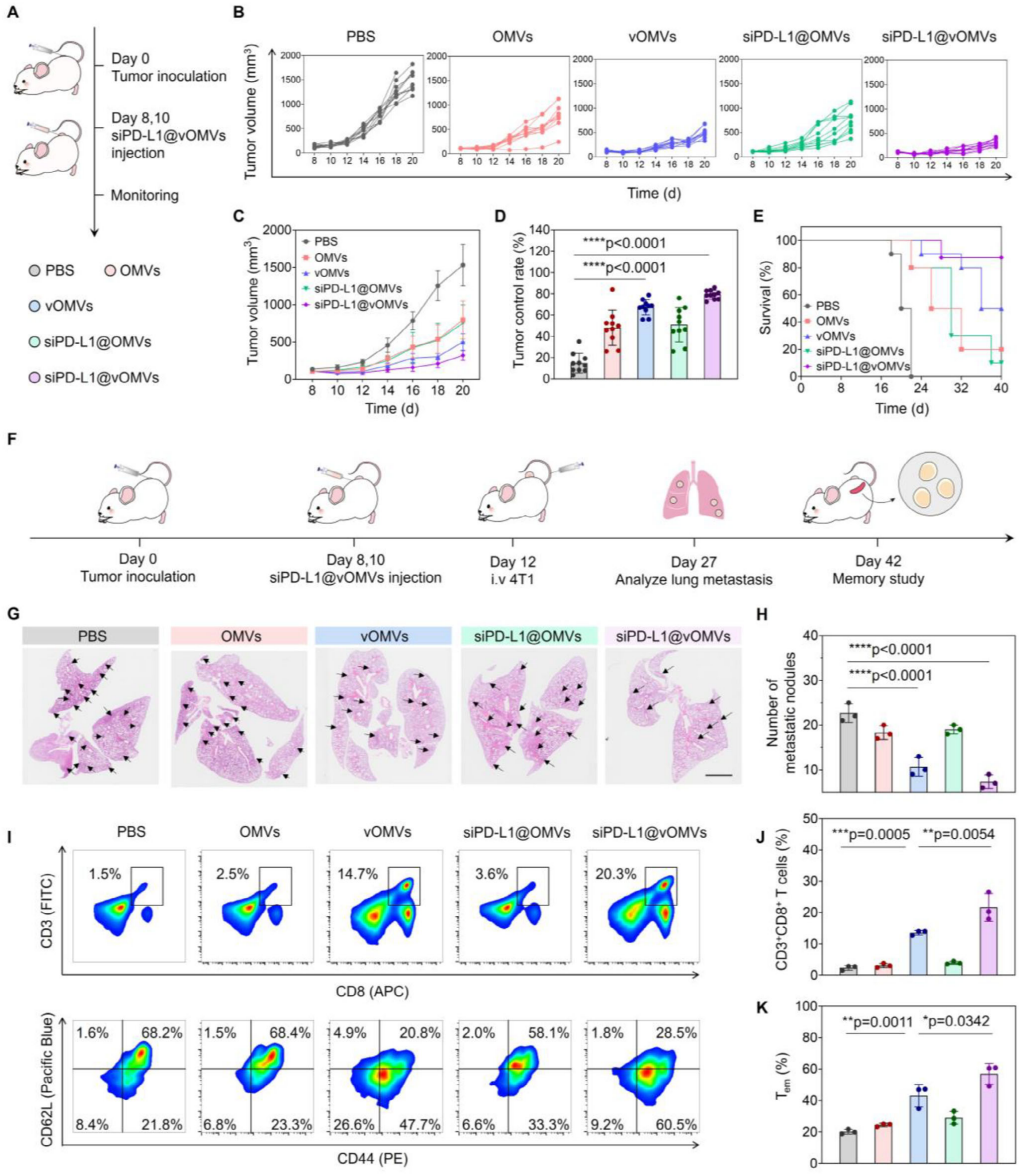
siPD‐L1@vOMVs treatment delays the growth of breast tumor and metastasis. A) Treatment schedule. BALB/c mice bearing 4T1 breast tumors were randomly divided into five groups and intratumorally injected with PBS, OMVs, vOMVs, siPD‐L1@OMVs, or siPD‐L1@vOMVs, and then the individual tumor volumes were recorded every 2 days. B,C) Tumor growth curves and volumes of mice receiving different treatments. D) Tumor control rate. E) Survival curve of each group. F) Schematic illustration of siPD‐L1@vOMVs‐triggered cancer immunotherapy in a 4T1 metastasis model. 4T1 tumor‐bearing mice were randomly divided into five groups and treated with different formations intratumorally. Two days after the last administration, 3 × 10^5^ 4T1 cells were intravenously injected into the mice to mimic a simulation of hematological metastasis. For quantification of pulmonary tumor nodules, lung sections were stained with H&E, and the metastatic nodules were manually demarcated. G) Representative H&E‐stained lung slices and the metastatic nodules were outlined with black arrows. Scale bar: 2 mm. H) Numbers of lung metastatic nodules in each group. I) Flow cytometry data of CD3^+^CD8^+^ and CD3^+^CD8^+^CD44^+^CD62L^−^ T lymphocytes in spleens. J,K) Quantification of expression levels of CD3^+^CD8^+^ T lymphocytes and CD3^+^CD8^+^CD44^+^CD62L^−^ T lymphocytes in spleens. Data in B‐E are presented as mean ± s.d. (n = 10 biologically independent mice). Data in H, I, and J are presented as mean ± s.d. (n = 3 biologically independent mice). Statistically significant differences between groups were identified by an unpaired two‐tailed Student's t‐test. ^****^ *P* < 0.0001, ^***^ *P* < 0.001, ^**^ *P* < 0.01, ^*^ *P* < 0.05, n.s., not significant.

Considering the efficient host immunity and therapeutic effects of siPD‐L1@vOMVs, we further explored its performance on the metastatic tumor models (**Figure** **6**A). Treatment with OMVs or siPD‐L1@OMVs delayed the growth of breast tumors, yet was insufficient to generate an appreciable antitumor outcome (Figure 6B–D). By contrast, vOMVs exhibited prominent antitumor effects and remarkably improved the survival of mice (Figure 6E). Notably, siPD‐L1@vOMVs significantly suppressed tumor development with a tumor inhibitory rate near 80%. In addition, the survival rate reached 90% during 40 days with a negligible decrease in body weight, while mice in other groups gradually died within 30 days (Figure S33, Supporting Information), which further verified that the tumor foreignization strategy by siPD‐L1@vOMVs is effective in treating different kinds of cancers and more importantly, not restricted by tumors with distinct immunogenicity.

We then evaluated its potential in resisting the metastasis of breast cancer (Figure 6F). As shown in the representative histological images, obvious lung metastatic foci were found in PBS, OMVs as well and siPD‐L1@OMVs groups (Figure 6G). On the contrary, the mice treated with vOMVs and siPD‐L1@vOMVs showed significant regression of the reinjected 4T1 tumors, and the number of metastatic nodules in siPD‐L1@vOMVs was reduced by more than 50% compared with other groups (Figure 6H). Correspondingly, a 5‐fold increase of CD8^+^ T cells was found in the spleens of siPD‐L1@vOMVs‐treated mice compared with other groups (Figure 6I,J; Figure S34, Supporting Information). As is known, CD8^+^ T cells were tightly coupled with the memory T‐cell differentiation.^[^ ^39^ ^]^ As expected, the frequency of effector memory T cell (Tem) (CD3^+^CD8^+^CD44^+^CD62L^−^) in splenic lymphocytes increased to almost 60%, while it was only 29.0% in the siPD‐L1@OMVs group (Figure 6K). These evoked Tem would induce systemic and long‐term T cell memory, thereby contributing to the enhanced anti‐metastatic effects.

## 3 Conclusion

siPD‐L1@vOMVs can represent a novel priming‐boosting strategy for reinvigorating T cell responses by the tumor foreignization strategy. On one side, VSVG‐equipped OMVs can transfer the pathogen‐associated molecular patterns (PAMPs) and classic bacterial antigens to the tumor surface through the acidic tumor microenvironment (TME)‐induced membrane fusion, not only reprograming tumor immunogenicity but also recruiting more APCs to the tumor tissues. For another, by virtue of the innate immunity against the invasion of foreign microorganisms, the vOMVs‐foreignized tumor cells (fTCs) mobilize APCs to engulf the tumor cells and present the whole‐cell tumor antigens, then potentiating the antigen‐specific immunity. By loading siPD‐L1 into vOMVs, the foreignization of tumor cells is synergized with the direct delivery of gene therapeutics siPD‐L1 into the cytoplasm, silencing PD‐L1 and further reverting T cell‐mediated attack. Therefore, potent therapeutic effects are achieved in the subcutaneous, bilateral, as well as metastatic tumors, validating its broad application prospects, particularly the cancer subtypes with distinguishing immunogenic properties.

This foreignization strategy provokes the elimination of fTCs by APCs, resulting in a diverse phenotypic profile of T cells, including both tumor and pathogen‐specific T cells. Although persistent antigen stimulation during cancer or pathogenic infections may lead to T cell exhaustion,^[^ ^40^ ^]^ we find that the adaptive immunity is elevated to an extremely high level in this study, not only awakening the systemic antigen‐specific and renewed memory immune responses but also upregulating the effector T lymphocytes in TME (even more than 50 folds than that of PBS control). The enhanced helper CD4^+^ T cells possess direct cytotoxicity in killing tumor cells, meanwhile synergizing with CD8^+^ T to promote their effector functions and prevent exhaustion,^[^ ^41^ ^]^ thereby a robust adaptive immunity is induced by siPD‐L1@vOMVs. The bacterial antigens in vOMVs can also enforce the microbe‐specific T cells during antibacterial immunity, yielding complementary antitumor effects. More importantly, OMVs may provide an alternative source of neoepitopes for cancer immunotherapy. Although microbe‐specific T cells can be activated in a TCR‐independent manner upon exposure to pro‐inflammatory cytokines (such as type I interferon and interleukin‐12) or immunogenic microbial molecules,^[^ ^33^ ^]^ the MHC‐bound antigens may dictate T‐cell function more specifically and underly a significant part of protective immunity. Considering that the delivery of viral epitopes to MHC molecules has been proven to harness cytomegalovirus‐specific T cells for tumor therapy,^[^ ^42^ ^]^ it is promising that “OMVs epitopes” on foreignized tumors will guide the returned bacteria‐specific T cells against cancer once “OMVs epitopes” can be presented by MHC molecules.

In essence, the treatment mechanism of the foreignization nanosystem siPD‐L1@vOMVs is consistent with that of the pharmaceutical vaccines. OMV‐based vaccines known as 4CMenB have been licensed in more than 35 countries, emphasizing the great potential of OMVs as a vaccine platform. Also, in situ tumor antigens engulfed by APCs in this study contain a whole array of epitopes, providing a novel avenue for cancer vaccination since it has no restrictions on individual variation, cancer types, and heterogeneity, even promising for developing personalized tumor vaccines. Moreover, this tumor foreignization strategy is feasible in various cancers with quite different immunogenicity, such as low‐mutation burden types, immunologically “cold” colorectal or breast cancers, holding great foreground to boost the development of tumor vaccines in broad patient coverage.

## Conflict of Interest

The authors declare no conflict of interest.

## Author Contributions

W.‐R.Z., J. T, and H.‐Y.X. conceptualized and designed the research. W.‐R.Z., W.X., C.L., J.W., and P.L. performed the experiments. W.‐R.Z., Y.L., J.H., R.C. and W.N. collected and processed the data. All authors analyzed and interpreted the data. W.‐R.Z., J. Tang and H.‐Y.X. wrote the manuscript. All authors discussed the results and commented on the manuscript.

## Supporting information



Supporting Information

## Data Availability

The data that support the findings of this study are available from the corresponding author upon reasonable request.
