# Supplementary material for: Bacterial Foreignization Nanosystem Elicits Multi‐Phenotypic T Cells for Antitumor Immunity
Source: Adv Sci (Weinh). 2025 Jul 10;12(38):e04155. doi: 10.1002/advs.202504155 (PMC12520458; doi:10.1002/advs.202504155)
Supplement: Supplementary file 1 — Supporting Information [file ADVS-12-e04155-s001.docx]

Supporting Information

Bacterial Foreignization Nanosystem Elicits Multi-phenotypic T Cells for Antitumor Immunity

Wan-Ru Zhuang, Wen-Chi Xue, Chao Liang, Pan Liu, Yao Lei, Jiaqi He, Ran Cheng, Weidong Nie, Jianxiong Wang, Jie Tang, Hai-Yan Xie*

**Supplementary Figure Captions:**

**Table S1.** Representative proteins identified from vOMVs detected by proteomic analysis.

***Table S2.*** Representative bacterial proteins on the CT26 cell surface after vOMVs treatment detected by proteomic analysis.

***Table S3.*** Depth TCR repertoire analysis of Shannon.Index, Invsimpson.Index, Simpson.Index, Pielous, Singleton, DE50, and Convergence of T cells in tumor-draining lymph nodes (TDLNs) from PBS or vOMVs-treated mice bearing CT26 tumor.

***Figure S1.*** Expression of fusion protein ClyA-VSVG on engineered *E. coli* (A) and the derived vOMVs (C) verified by SDS-PAGE. B) Western blot analysis of GAPDH as loading control in *E. coli.*

***Figure S2.*** Size (A) and zeta potential (B) of OMVs or vOMVs. C) Stability of OMVs or vOMVs in PBS at 4 °C.

***Figure S3.*** Pie chart for the subcellular localization of the expressed proteins in OMVs detected by proteomic analysis.

***Figure S4.*** A) CLSM imaging of the CT26 tumor cells treated by OMVs. Enlarged CLSM images of tumor cells with different treatments were shown within the dashed box. B) 3D reconstruction imaging of vOMVs-fused tumor cells. C) Quantification of the mean fluorescence intensities (MFI) of VSVG and LPS on tumor cells in each group.

***Figure S5.*** A) Flow cytometry and quantification of VSVG^+^LPS^+^ tumor cells after different treatments. B) Representative flow cytometry plots and quantification of VSVG^+^ CT26 cells at different time points.

***Figure S6.*** Quantification of LPS^+^ tumor cells after different treatments detected by flow cytometry.

***Figure S7.*** Venn diagram of the proteins from vOMVs-fused CT26 cells (vOMVs-CT26) identified by proteomic analysis.

***Figure S8.*** A) Representative flow cytometry plots of CD80^+^, CD86^+^, or MHC-II^+^ cells in BMDMs after different treatments. Expression levels of CD86 (B) and MHC-II (C) on BMDMs analyzed by flow cytometry.

***Figure S9.*** A) Representative flow cytometry plots of CD80^+^, CD86^+^, or MHC-II^+^ cells in BMDCs after different treatments. Expression levels of CD86 (B) and MHC-II (C) on BMDCs analyzed by flow cytometry.

***Figure S10.*** Gating strategy for flow cytometry analysis of CD69^+^ T cells (gated on CD3^+^CD8^+^ cells).

***Figure S11.*** A) Gating strategy for flow cytometry analysis of LPS^+^ cells gated on CD45^-^ tumor cells or CD45^+^ immune cells. B) Gating strategy for flow cytometry analysis of VSVG^+^ cells gated on CD45^-^ tumor cells or CD45^+^ immune cells. C) Flow cytometry of VSVG^+^ tumor cells gated on CD45^-^ or CD45^+^ cells. D) Quantification of VSVG^+^ tumor cells gated on CD45^+^ or CD45^-^ cells.

***Figure S12.*** Gating strategy for flow cytometry analysis of CD45^+^ immune cells, F4/80^+^ macrophages, and CD11c^+^ DCs in tumor.

***Figure S13.*** Gating strategy for flow cytometry analysis of GFP^+^ cells gated on CD45^+^F4/80^+^ macrophages and CD45^+^CD11c^+^ DCs.

***Figure S14.*** Cdr3 length distribution of PBS group (among the top 10 representative cdr3 sequences).

***Figure S15.*** Encapsulation efficiency (A) and loading efficiency (B) of siRNA in vOMVs. Different amounts of siRNA were individually incubated with the same amount of vOMVs (10 µg). The loading was saturated at the ratio of 1:2 (vOMVs:siRNA).

***Figure S16.*** Size (A) and zeta potential (B) of siRNA@vOMVs. C) Stability of siRNA@vOMVs in PBS at 4 °C. D) Agarose gel electrophoresis of siRNA@vOMVs.

***Figure S17.*** Quantification of the internalization of Cy3 labeled-siRNA by CT26 cells.

***Figure S18.*** Colocalization ratio of siRNA and endosome calculated by Pearson’s correlation coefficient.

***Figure S19.*** Flow cytometry of expression levels of CD69 gated on CD8^+^ T cells. The gating strategy was the same as in Figure S10.

***Figure S20.*** Flow cytometry of (A) tumor-infiltrating CD45^+^ immune cells, (B) F4/80^+^ macrophages, and (C) CD11c^+^ DCs in tumor after different treatments.

***Figure S21.*** Gating strategy for flow cytometric studies of IFN-γ^+^ T cells (gated on CD3^+^CD8^+^ T cells) in tumors.

***Figure S22.*** Gating strategy for flow cytometry analysis of CD80^+^, CD86^+^ (A), and MHC^+^ (B) gated on CD11c^+^ cells in TDLNs.

***Figure S23.*** A) Representative flow cytometry plots of CD80^+^, CD86^+^, or MHC-II^+^ cells gated on CD11c^+^ cells from different treatment groups in TDLNs. B-D) Expression levels of CD80, CD86, and MHC-II gated on CD11c^+^ cells in TDLNs.

***Figure S24.*** A) Gating strategy for flow cytometry analysis of CD4^+^, CD8^+^ T cells gated on CD3^+^ cells in spleens. B) Percentages of CD3^+^ T cells from different treatment groups in spleens.

***Figure S25.*** Treatment of CT26 tumor-bearing mice by different groups. Individual tumor growth kinetics were recorded every two days.

***Figure S26.*** Gating strategy for flow cytometric studies of infiltrating T cells in primary or abscopal tumors. The tumors were isolated and analyzed on the 7th day after the final administration in the bilateral tumor model.

***Figure S27.*** A) Representative flow cytometry plots showing the CD3^+^ T cells in primary tumors. B) Percentages of CD3^+^CD4^+^ T cells in primary tumors. C) Representative flow cytometry plots showing the CD3^+^ T cells in abscopal tumors. D) Percentages of CD3^+^CD4^+^ T cells in abscopal tumors.

***Figure S28.*** Gating strategy for flow cytometric studies of CD8^+^IFN-γ^+^ T cells (gated on CD3^+^ T cells) in splenocytes after restimulation with CT26 antigens for 24 h.

***Figure S29.*** Hematoxylin and eosin (H&E) staining of primary and abscopal tumors at the end of the experiment.

***Figure S30.*** Body weight of mice during antitumor experiment against CT26.

***Figure S31.*** H&E staining of major organs at the endpoint of the antitumor experiment against CT26.

***Figure S32.*** Serum biochemical indexes of hepatic function biomarkers, such as alanine transaminase (ALT) (A), aspartate transaminase (AST) (B), and alkaline phosphatase (ALP) (C), renal function biomarkers including urea nitrogen (BUN) (D) and creatinine (Crea) (E), and cardiac function biomarkers including lactate dehydrogenase (LDH) (F) in different groups at the endpoint of the antitumor experiment against CT26.

***Figure S33.*** Body weight of mice during antitumor experiment against 4T1.

***Figure S34.*** Gating strategy for flow cytometric studies of CD3^+^CD8^+^ T lymphocytes and CD3^+^CD8^+^CD44^+^CD62L^−^ T lymphocytes (Tem) in spleens.

**1. Materials and Methods**

**1.1. Materials and reagents**

DMEM, RPMI-1640, Opti-MEM cell culture medium, fetal bovine serum (FBS), and 0.25 % trypsin-ethylenediaminetetraacetic acid (EDTA) were purchased from Gibco (USA). Penicillin-streptomycin was purchased from Hyclone (USA). Micro BCA Protein Assay Kit and Prestained Protein Ladder were purchased from Thermo Scientific (USA). Kanamycin, isopropyl β-D-1-thiogalactopyranoside (IPTG), coomassie brilliant blue, SDS-PAGE loading buffer, and RNase-free water were purchased from Solarbio (China). Hoechst 33342 was purchased from Life Technologies (USA). iFluor®555-Wheat Germ Agglutinin (WGA) Conjugate was purchased from AAT Bioquest (USA). Recombinant murine IL-4, macrophage colony-stimulating factor (M-CSF), and granulocyte-macrophage colony-stimulating factor (GM-CSF) were purchased from PeproTech (USA). FITC-conjugated anti-VSVG antibody (catalogue no. bs-2110R-FITC), anti-VSVG antibody (catalogue no. bs-2110R), FITC-conjugated anti-E. coli LPS antibody (catalog no. bs-8000R-FITC) and Cy5-conjugated anti-E. coli LPS antibody (catalog no. bs-8000R-Cy5) were purchased from Bioss (China). Anti-PD-L1 (catalogue no. ab213480), anti-β-Actin (catalogue no. ab8227) and Goat-Anti-Rabbit IgG (HRP) (catalogue no. ab6721) antibodies were purchased from Abcam (UK). LysoTracker™ Green DND-26 was purchased from Invitrogen (USA). True-Nuclear™ Transcription Factor Buffer Pacific Blue-conjugated anti-mouse CD45 antibody (catalogue no. 103125), PE-conjugated anti-mouse F4/80 antibody (catalogue no. 157303), FITC-conjugated anti-mouse F4/80 antibody (catalogue no. 123107), PE-conjugated anti-mouse CD69 (catalogue no. 104507), PE-conjugated anti-mouse CD4 (catalogue no. 100408), FITC-conjugated anti-mouse CD3 (catalogue no. 100203), APC-conjugated anti-mouse CD3 (catalogue no. 100236), FITC-conjugated anti-mouse CD8 (catalogue no. 100705), APC-conjugated anti-mouse CD8 (catalogue no. 140410), PE-conjugated anti-mouse IFN-γ (catalogue no. 163503), FITC-conjugated anti-mouse CD80 (catalogue no. 104706), PE-conjugated anti-mouse CD86 (catalogue no. 159203), APC-conjugated anti-mouse CD86 (catalogue no. 105011), APC-conjugated anti-mouse MHC-II (catalogue no. 116417), Pacific Blue-conjugated anti-mouse MHC-II (catalogue no. 107619), APC anti-mouse CD11b (catalogue no. 101211), APC-conjugated anti-mouse CD11c (catalogue no. 117309), PE-conjugated anti-mouse CD11c (catalogue no. 117307), Pacific Blue-conjugated anti-mouse CD62L (catalogue no. 104423) and PE-conjugated anti-mouse CD44 (catalogue no. 103023) were purchased from BioLegend (USA). ProteinExt Mammalian Membrane Protein Extraction Kit was purchased from TransGen Biotech (China). Carboxyfluorescein succinimidyl ester (CFSE) was purchased from Beyotime (China). siRNA targeting mouse PD-L1 (siPD-L1, sense strand: 5′-AGACGUAAGCAGUGUUGAAdT-s-dT-3′, antisense: 3′-UUCAACACUGCUUACGUCUdT-s-dT-5′), the negative control siRNA (scrambled siRNA) and Cy3-labeled siRNA were synthesized from Suzhou Biosyntech (China). Cy3 fluorophore was labeled at the 5′-end of the sense strand. Flow Cytometry Staining Buffer was purchased from eBioscience (China).

**1.2. Cell lines and animals**

CT26 (mouse colon cancer cell line) and 4T1 (mouse breast cancer cell line) were kindly obtained from the Institute of Process Engineering (China). All cell lines were maintained in a 37 °C humidified chamber with 5 % CO_2_ and cultured in DMEM medium containing 10 % FBS and 1 % penicillin-streptomycin.

Female BALB/c mice were purchased from Beijing Vital River Laboratory Animal Technology Co. Ltd (Beijing, China). Mice used in these studies were 5 weeks old at the start of the experiment. Mice were housed in a room with a temperature of 20-22 °C and a humidity of 30-70%. Feed and water were available. Artificial light was provided in a 12-h light/12-h dark cycle. All care and handling of animals were performed with the approval of the Ethics Committee of Peking University (protocol number LA2019039).

**1.3. Plasmid construction, bacterial strain, and growth**

The recombinant pET28a-ClyA-VSVG plasmid was obtained from GENEWIZ (China). Briefly, the cytotoxin ClyA gene was amplified from E. coli (strain K12) genomic DNA and cloned into the pET28a vector at the EcoRI restriction site. Then, the ectodomain of VSVG was placed downstream of ClyA at the EcoRI and XhoI restriction sites, yielding a fused protein where VSVG is fused to the C-terminus of ClyA. The flexible linker sequence SSSSGSSSSG was inserted between both ClyA and VSVG. The E. coli production strain BL21 (Transgen, China) was transformed with the expression plasmid pET28a-ClyA-VSVG and grown at 37 °C in LB medium with 50 μg/ml Kanamycin, with shaking at 180 rpm, until the OD600 was 0.5. Then, 0, 0.5, or 1 mM IPTG was respectively added to further induce protein expression at 25 °C, and the incubation was continued for 14 h with shaking at 160 rpm. Protein samples extracted from bacteria were separated with SDS polyacrylamide gel electrophoresis and blotted onto polyvinylidene difluoride (PVDF) membrane. The membrane was blocked with 5% skim milk powder for 3 h, and incubated with anti-VSV-G antibody (1:2500) overnight at 4 °C. After washing with PBST (phosphate-buffered saline with Tween20) for three times, the membrane was further incubated with a diluted secondary antibody Goat-Anti-Rabbit IgG (HRP) (1:10000) for 2 h at 25 °C with shaking at 70 rpm. Then the PVDF membrane was washed by PBST for three times and visualized by IBright Imaging Systems (Thermo, USA).

**1.4. Preparation of OMVs, vOMVs, and siRNA@vOMVs**

The normal E. coli or transformed E. coli was utilized for the generation of OMVs or vOMVs. Briefly, bacterial cells were removed by centrifuging at 10000 g for 10 min at 4 °C (Sorvall ST16R, Thermo, USA). The obtained supernatant was filtered by 0.45 μm filters (Vacuum Filter System, Corning, USA) and concentrated by centrifugal filters with a molecular weight cutoff (MWCO) of 100 kDa (Millipore, USA). The concentrated supernatant was filtered again through 0.22 μm pore size filters (Millipore, USA) to remove any remaining debris or bacteria. Then, the concentrated medium was centrifuged at 150000 g for 3 h at 4 °C (XPN 100, Beckman, USA). The supernatant was removed and the pellet was resuspended in PBS and stored at -80 °C. The protein concentration of OMVs or vOMVs was determined by the Micro BCA Protein Assay Kit. Protein samples extracted from OMVs or vOMVs were separated with SDS polyacrylamide gel electrophoresis and blotted onto polyvinylidene difluoride (PVDF) membrane. The membrane was blocked with 5% skim milk powder for 3 h, and incubated with anti-VSV-G antibody (1:2500) overnight at 4 °C. After washing with PBST (phosphate-buffered saline with Tween20) for three times, the membrane was further incubated with a diluted secondary antibody Goat-Anti-Rabbit IgG (HRP) (1:10000) for 2 h at 25 °C with shaking at 70 rpm. Then the PVDF membrane was washed by PBST for three times and visualized by IBright Imaging Systems (Thermo, USA).

For the preparation of siRNA@vOMVs, vOMVs and siRNA were mixed in PBS with a volume of 400 µL at a ratio of 1:2 (w/w) and pulsed in a 4 mm cuvette (Bio-Rad, USA) under conditions of 100 V, 100 µF, and 200 Ω by the GenePulser electroporators (Bio-Rad, USA). After electroporation, the mixtures were then incubated for 1 h at 25 °C. Unloaded free siRNA was removed by ultracentrifugation at 150000 g for 3 h at 4 °C (XPN 100, Beckman, USA). The pellet containing siRNA@vOMVs was resuspended in PBS and stored at -80 °C. To measure the loading efficiency of siRNA, standard Cy3-siRNA solutions were prepared with known concentrations, and a standard curve was established by detecting the fluorescence signal of Cy3 by UV-spectrophotometry (Multiskan Sky, Thermo, USA). Then, vOMVs solution with 10 μg protein was diluted in PBS and mixed with different amounts of Cy3-labeled siRNA to a total volume of 200 μL and incubated on ice for 10 min. Electroporation was carried out in a 4 mm gap cuvette under conditions of 100 V, 200 Ω, and 100 μF. After that, residual siRNA was removed by ultracentrifugation at 150000 g for 3 h at 4 °C (XPN 100, Beckman, USA). The content of siRNA in the supernatant was determined by UV-vis spectrum to ensure the variety of loading efficiency and encapsulation efficiency by UV-spectrophotometry.

**1.5. Characterization of OMVs, vOMVs, and siRNA@vOMVs**

The sample morphology of OMVs, vOMVs, or siRNA@vOMVs was observed by the transmission electron microscope (TEM) (120 kV, Tecnai Spirit, USA). The samples were negatively stained with 2% uranyl acetate solution in distilled water before observation. The hydrodynamic sizes and zeta potentials were measured by dynamic light scattering (DLS) (Malvern Instruments). To test the stability of vesicles, 10 µg of OMVs, vOMVs, or siRNA@vOMVs was incubated with 1 mL PBS for 7 days at 4 °C and the hydrodynamic diameters were recorded by DLS. The protein distributions of OMVs, vOMVs, and siRNA@vOMVs were detected by SDS-PAGE electrophoresis, and proteins were stained with Coomassie brilliant blue for 2 h and then imaged to further analyze the proteins in these vesicles. For the proteomic analysis, the trypsin was first digested with vOMVs or OMVs, and then the polypeptides were subsequently detected via liquid chromatography-mass spectrometry (LC-MS). The identification results were analyzed to confirm the protein components of the vOMVs or OMVs. The stability of siRNA in prepared siRNA@vOMVs was investigated by agarose gel electrophoresis. Given the relatively small molecular weight of siRNA (~15 kDa), the integrity of siRNA was assessed using 4% agarose gel electrophoresis.

**1.6. *In vitro* membrane fusion ability of vOMVs**

CT26 cells (1 × 10^5^ cells/well) were seeded in confocal culture dishes and cultured overnight in DMEM containing 10% FBS. Then the medium was replaced by Opti-MEM with pH of 7.4 or 6.5. Then, OMVs or vOMVs (50 µg/mL) were respectively added into the cells with different Opti-MEM medium with pH of 7.4 or 6.5 and incubated for 30 min at 37 °C. Afterward, cells were stained with iFluor®555-conjugated WGA (1:500) and Hoechst 33342 (1:1000) for 20 min at 37 °C. Then, cells were washed with PBS for three times and stained with Cy5-conjugated anti-E. coli LPS antibody (1:250) and FITC-conjugated anti-VSVG antibody (1:500) for 30 min at 4 °C. Then, cells were observed by confocal microscopy (Eclipse-Ti2, Nikon, Japan).

For flow cytometry analysis, CT26 cells (5 × 10^4^ cells/well) were seeded in a 24-well plate and cultured overnight in DMEM containing 10% FBS. Then the medium was replaced by Opti-MEM with pH of 7.4 or 6.5. Then, OMVs or vOMVs (50 µg/mL) were respectively added into the cells with different Opti-MEM medium with pH of 7.4 or 6.5 and incubated for 30 min at 37 °C. Afterward, cells were stained with Cy5-conjugated anti-E. coli LPS antibody (1:250) and FITC-conjugated anti-VSVG antibody (1:500) for 30 min at 4 °C. Then, cells were detected by the flow cytometer (Bioscience FACSAria, BD, USA).

For the proteomic analysis, CT26 cells (5 × 10^5^ cells/well) were seeded in a 6-well plate and cultured overnight in DMEM containing 10% FBS. Then the medium was replaced by Opti-MEM with pH of 6.5. OMVs or vOMVs (50 µg/mL) were respectively added into the cells with different Opti-MEM medium with pH of 6.5 and incubated for 30 min at 37 °C. Afterward, cells were washed with PBS for three times and a membrane extraction kit was used to extract membrane proteins from CT26 cells with different treatments. The trypsin was first digested with membrane proteins from CT26 cells, then the polypeptides were subsequently detected via LC-MS. The identification results were analyzed to confirm the protein components of the cell membranes. Briefly, the proteomic data were analyzed by PEAKS Studio 10.6.0. Carbamidomethyl (C) was set as a fixed modification, while oxidation (M) and N-terminal acetylation were allowed as variable modifications. Trypsin digestion with up to 2 missed cleavages was specified. Mass tolerances were set to 20 ppm for both precursor and fragment ions. Searches were performed against the database of *Escherichia coli*. To search for the subcellular location of a protein, we used its primary accession number in UniProt (https://www.uniprot.org/, 2025). ﻿As a result, 63 bacterial proteins were identified on the cell membrane after vOMVs treatment at pH 6.5, and their subcellular location in *Escherichia coli* (including periplasmic proteins, outer membrane proteins, inner membrane proteins, and cytoplasmic proteins) were concluded.

To determine the mechanism of membrane fusion ability of vOMVs, CT26 cells (5 × 10^4^ cells/well) were seeded in a 24-well plate and cultured overnight in DMEM containing 10% FBS. vOMVs were prelabeled with FITC-conjugated LPS antibody and incubated at 37 °C for 1 h. The free antibody was removed by centrifugal filters with MWCO of 300 kDa by Opti-MEM with pH 6.5 (Pall, USA). Then, FITC-labeled vOMVs were cultured with anti-VSVG antibody (1:100) for 1 h at 25 °C to block the VSVG protein on the surface of vOMVs. Afterward, FITC-labeled vOMVs or pre-blocked vOMVs were respectively added into the cells with Opti-MEM medium with pH of 6.5 and incubated for 30 min at 37 °C. The obtained cells were washed with PBS for three times and detected by flow cytometer.

**1.7. Activation of BMDMs and BMDCs and phagocytosis of tumors by BMDMs or BMDCs**

Murine bone marrow-derived dendritic cells (BMDCs) from marrow cavities of femurs and tibias of C57BL/6 mice were cultivated in plates with a medium containing 20 ng/mL GM-CSF and 20 ng/mL IL-4 for 7 days. Murine bone marrow-derived macrophages (BMDMs) from C57BL/6 mice were cultivated in plates with a medium containing 20 ng/mL M-CSF and 20 ng/mL IL-4 for 7 days. Then, BMDCs or BMDMs were seeded in 24-well plates at a density of 5 × 10^5^ per well. Then, PBS, LPS (1 µg/mL), OMVs (10 µg/mL), and vOMVs (10 µg/mL) were respectively added into BMDCs or BMDMs for 24 h. Then, BMDCs or BMDMs were collected and stained with fluorescent antibodies of CD11c/CD11b, CD80, CD86, and MHCII (1:100) individually, and measured by flow cytometry.

For tumor phagocytosis analysis, CT26 cells (5 × 10^4^ cells/well) were seeded in a 24-well plate and cultured overnight in DMEM containing 10% FBS and then CT26 were labeled with 5 µM CFSE at 37 °C for 20 min. After washing for three times, PBS, OMVs, or vOMVs (10 µg/mL) were respectively added into the CFSE-labeled cells with Opti-MEM medium with pH 6.5 and incubated for 30 min at 37 °C. Then, the supernatant, as well as the treated tumor cells, were collected and added into BMDMs or BMDCs (ratio=1:1). Following the mixed incubation for 12 h, BMDMs or BMDCs in mixed cells were labeled with APC-conjugated anti-CD11b antibody or anti-CD11c antibody. Then, the CFSE signal in the gate of CD11b or CD11c was analyzed by flow cytometer.

**1.8. The cellular uptake and subcellular localization of siRNA@vOMVs**

To verify the internalization and intracellular distribution of siRNA@vOMVs in cells, CT26 cells (1 × 10^5^ cells/well) were seeded in confocal culture dishes and cultured overnight in DMEM containing 10% FBS. Then, OMVs or vOMVs were mixed with Cy3-labeled siRNA in Opti-MEM medium with pH of 6.5 at a ratio of 1:2 (w/w) and pulsed in a 4 mm cuvette (Bio-Rad, USA) under conditions of 100 V, 100 µF, and 200 Ω by the GenePulser electroporators (Bio-Rad, USA). After electroporation, the mixtures were then incubated for 1 h at 25 °C. The obtained siRNA@OMVs, siRNA@vOMVs as well as free Cy3-labeled siRNA were respectively added into the CT26 cells with Opti-MEM medium with pH of 6.5 and incubated for 4 h at 37 °C (all containing 20 µg/mL siRNA). Afterward, parts of the CT26 cells were stained with Hoechst 33342 (1:1000) for 20 min at 37 °C and then stained with LysoTracker (1:1000) for 45 min at 37 °C. The final cells were detected by flow cytometer or confocal microscopy, respectively.

**1.9. Western Blot analysis of gene silencing efficiency of siPD-L1@vOMVs**

CT26 cells (5 × 10^5^ cells/well) were seeded in a 6-well plate and cultured overnight in DMEM containing 10% FBS. Then, free siPD-L1, siPD-L1@OMVs, or siPD-L1@vOMVs were respectively added into the CT26 cells with Opti-MEM medium with pH of 6.5 and incubated for 48 h at 37 °C (all containing 2 µg/mL siPD-L1). Protein samples extracted from the above cells were separated with SDS polyacrylamide gel electrophoresis and blotted onto the nitrocellulose (NC) membrane (Millipore). The membrane was blocked with 5% skim milk powder for 3 h, and incubated with anti-PD-L1 antibody (1:1000) or anti-β-Actin antibody (1:10000) overnight at 4 °C. After washing with PBST (phosphate-buffered saline with Tween20) for three times, the membrane was further incubated with a diluted secondary antibody Goat-Anti-Rabbit IgG (HRP) (1:10000) for 2 h at 25 °C with shaking at 70 rpm. Then the PVDF membrane was washed by PBST for three times and visualized with ECL reagent (Thermo, USA) by Chemiluminescence system (ChemiDoc XRS+ System, Bio-Rad).

**1.10. *In vitro* activation of T cells**

CT26 cells (5 × 10^4^ cells/well) were seeded in a 24-well plate and cultured overnight in DMEM containing 10% FBS. Then, PBS, OMVs, vOMVs, siPD-L1@OMVs, or siPD-L1@vOMVs (containing 10 µg/mL vesicles and 4.3 µg/mL siPD-L1) were respectively added into tumor cells with Opti-MEM medium with pH 6.5 and incubated for 30 min at 37 °C. Then, the supernatant as well as the treated tumor cells were collected and added into BMDMs or BMDCs (ratio=1:1) for further incubation for 24 h. Splenocytes were isolated from the spleens of C57BL/6 mice (aged 6−8 weeks) and CD8^+^ T cells were isolated using a CD8^+^ no-touch isolation kit (Miltenyi Biotec) according to the manufacturer’s guidelines. Afterward, T cells were co-incubated with the above BMDMs or BMDCs for 24 h at a ratio of 4:1. Then, T cells were collected and labeled with APC-conjugated anti-mouse CD3, FITC-conjugated anti-mouse CD8 as well as PE-conjugated anti-mouse CD69, and analyzed by flow cytometer.

**1.11. *In vivo* innate immune activation assay**

For *in vivo* tumor foreignization analysis, BALB/c mice were subcutaneously incubated with CT26 tumor cells (1 × 10^6^). When tumors reached about 300 mm^3^, mice were randomly divided into three groups, and intratumorally injected with 100 µL of PBS, OMVs, and vOMVs (vesicle dose: 10 μg per mouse). After 4 h, mice were sacrificed and tumor tissues were surgically collected for further investigation. Briefly, tumors were cut into small pieces and then homogenized in cold PBS to form the single-cell suspension. The obtained cells were stained with fluorescent antibodies of CD45, LPS and VSVG. Then, stained cells from tumors were washed with PBS three times and measured by flow cytometer.

For *in vivo* APCs recruitment analysis, BALB/c mice were subcutaneously incubated with CT26 tumor cells (1 × 10^6^). When tumors reached about 300 mm^3^, mice were randomly divided into three groups, and intratumorally injected with 100 µL of PBS, OMVs, vOMVs, siPD-L1@OMVs or siPD-L1@vOMVs (vesicle dose: 10 μg per mouse; siPD-L1 dose: 4.3 μg per mouse). After 48 h, mice were sacrificed and tumor tissues were surgically collected for further investigation. Briefly, tumors were cut into small pieces and then homogenized in cold PBS to form the single-cell suspension. The obtained cells were stained with fluorescent antibodies of CD45, F4/80, and CD11c. Then, stained cells from tumors were washed with PBS three times and measured by flow cytometer.

For *in vivo* tumor phagocytosis analysis, BALB/c mice were subcutaneously incubated with GFP-CT26 tumor cells (1 × 10^6^). When tumors reached about 300 mm^3^, mice were randomly divided into three groups, and intratumorally injected with 100 µL of PBS, OMVs, and vOMVs (vesicle dose: 10 μg per mouse) every 2 days for two times. After 48 h, mice were sacrificed and tumor tissues were surgically collected for further investigation. Briefly, tumors were cut into small pieces and then homogenized in cold PBS to form the single-cell suspension. The obtained cells were stained with fluorescent antibodies of CD45. Then, stained cells from tumors were washed with PBS three times and measured by flow cytometer.

**1.12. Bulk TCR amplification, sequencing, and data analysis**

BALB/c mice were subcutaneously incubated with CT26 tumor cells (1 × 10^6^). When tumors reached about 300 mm^3^, mice were randomly divided into three groups, and intratumorally injected with 100 µL of PBS and vOMVs (vesicle dose: 10 μg per mouse) every 2 days for two times. On the 7th day after the final administration, the TDLNs were surgically collected and the RNA samples from TDLNs were analyzed by high-throughput sequencing of TCR using the ImmuHub TCR profiling system at the deep level (ImmuQuad Biotech, China). Briefly, a 5′ RACE unbiased amplification protocol was used, and the sequencing was performed on an Illumina HiSeq X10 system with PE150 mode. The raw sequencing data were then aligned with NCBI and PCR. The resulting nucleotide and amino acid sequences of CDR3 of TCRβ were determined, and those with out-of-frame and stop codon sequences were removed from the identified TRB repertoire. We further defined the amount of each TCRβ clonotype by adding numbers of TCRβ clones sharing the same nucleotide sequence as CDR3. Shannon's index, Inverse Simpson index, Simpson's Index, Pielou's Index, Singleton, DE50, and TCR Convergence were used to assess the diversity of TCR clones within each sample, which was defined as follows:

Shannon's index: $H^{'}=-\sum pi(\ln\mathrm{pi})$;

Inverse Simpson index: $\frac{1}{D}=1/\sum_{i=1}^{N} \mathrm{pi}^{2}$;

Simpson's Index: $1-D=1-\sum ni(ni-1)/N(N-1)$;

Pielou's Index: $Pielou's=-\sum\frac{\mathrm{pi}\left( \ln\mathrm{pi} \right)}{\ln S}$;

Singleton: $Singleton=\frac{Single clone counts}{Total clone counts}$;

DE50:$DE50=\frac{nb rearrangements accounting for 50\% total map intensity}{Total nb rearrangements present}$;

TCR Convergence: $Convergence=\sum\mathrm{pi}$;

**1.13. Activation of pathogen-specific T cells**

To detect the existence and activity of pathogen-specific T cells (Tps), BALB/c mice were subcutaneously incubated with CT26 tumor cells (1 × 10^6^). When tumors reached about 300 mm^3^, mice were randomly divided into three groups, and intratumorally injected with 100 µL of PBS and vOMVs (vesicle dose: 10 μg per mouse) every 2 days for four times. On the 10th day after the final administration, TDLNs and spleens were surgically collected for enzyme-linked immunospot (ELISpot) assay. Briefly, splenocytes or cells from TDLNs were seeded at 1 × 10^5^ cells per well in a 96-well plate coated with a mouse anti-IFN-γ antibody and incubated for 48 h with peptide antigen from vOMVs. The secreted and captured IFN-γ was subsequently detected using a biotinylated antibody specific for IFN-γ and alkaline-phosphatase conjugated to streptavidin. After the addition of the substrate solution, a red precipitate formed and appeared as spots at the sites of cytokine production. Automated spot quantification was performed by AID vSpot-High Resolution Image Analyzer (Germany).

To explore the efficacy of Tps against tumors *in vivo*, CT26 cells were cultured overnight in DMEM containing 10% FBS. Then, PBS or vOMVs (50 µg/mL) were respectively added into tumor cells with Opti-MEM medium of pH 6.5 and incubated for 30 min at 37 °C. Afterward, BALB/c mice were subcutaneously incubated with treated CT26 cells (1 × 10^6^). On day 11 after tumor engraftment, appeared tumors in the PBS or vOMVs group were adoptively treated with antigen-prestimulated T cells obtained from above (P-T and vO-T, respectively) every 2 days for three times. The length (L) and width (W) of the subcutaneous tumors were measured every other day after the first administration. The tumor volumes were calculated by the formula of (L × W^2^)/2. When the tumor volume was larger than 1000 mm^3^, the mice were euthanized.

**1.14. Systemic immune activation assay**

BALB/c mice were subcutaneously incubated with CT26 tumor cells (1 × 10^6^). When tumors reached about 300 mm^3^, mice were randomly divided into five groups, and intratumorally injected with 100 µL of PBS, OMVs, vOMVs, siPD-L1@OMVs or siPD-L1@vOMVs (vesicle dose: 10 μg per mouse; siPD-L1 dose: 4.3 μg per mouse). After 48 h, mice were sacrificed, and TDLNs, spleens, and tumor tissues were surgically collected for further investigation. Briefly, TDLNs, spleens, and tumors were cut into small pieces and then homogenized in cold PBS to form the single-cell suspension. The obtained cells were divided into several parts to analyze different immune cell types, respectively. For cytotoxic T lymphocytes (CTL) analysis, cells from tumors were stained with CD3, CD8, and IFN-γ antibodies. Then, stained cells from tumors were washed with PBS three times and measured by flow cytometer. For DC maturation analysis, cells from TDLNs were stained with CD11c, CD80, CD86, and MHC-II antibodies. For CD8^+^ T cells analysis, cells from spleens were stained with CD3, CD4 and CD8 antibodies.

**1.15. *In vivo* antitumor activity**

To establish a bilateral tumor model, 1 × 10^6^/100 μL CT26 tumor cells in PBS were subcutaneously transplanted into the right flank of BALB/c mice as the primary tumor. After 5 days, to form the abscopal tumor, 1 × 10^6^ CT26 tumor cells were subcutaneously injected into the left flank. When the primary tumor volume reached about 100 mm^3^, mice were randomly divided into five groups, and intratumorally injected with 100 µL of PBS, OMVs, vOMVs, siPD-L1@OMVs or siPD-L1@vOMVs (vesicle dose: 10 μg per mouse; siPD-L1 dose: 4.3 μg per mouse) every 2 days for two times. The length (L) and width (W) of the subcutaneous tumors and the body weights were measured every other day after the first administration. The tumor volumes were calculated by the formula of (L × W^2^)/2. When the tumor volume was larger than 1000 mm^3^, the mice were euthanized. At the end of therapy, some mice were sacrificed and the main organs (heart, liver, spleen, lung, and kidneys) as well as bilateral tumors were harvested, fixed in 4 % paraformaldehyde solution, and sectioned into slices for the hematoxylin and eosin (H&E) or Ki67 staining. For safety evaluation, the serum levels of alkaline phosphatase (ALP), alanine aminotransferase (ALT), aspartate transaminase (AST), blood urea nitrogen (BUN), lactate dehydrogenase (LDH) and creatinine (CREA) at the endpoint of the experiment were analyzed.

For 4T1 tumor inhibition, BALB/c mice were subcutaneously incubated with 4T1 tumor cells (1 × 10^6^). When tumors reached about 100 mm^3^, mice were randomly divided into five groups, and intratumorally injected with 100 µL of PBS, OMVs, vOMVs, siPD-L1@OMVs or siPD-L1@vOMVs (vesicle dose: 10 μg per mouse; siPD-L1 dose: 4.3 μg per mouse) every 2 days for two times. The tumor volumes were calculated by the formula of (L × W^2^)/2. The body weight was measured every 2 days after the first administration.

**1.16. T-cell infiltration in the tumor microenvironment**

To establish a bilateral tumor model, 1 × 10^6^/100 μL CT26 tumor cells in PBS were subcutaneously transplanted into the right flank of BALB/c mice as the primary tumor. After 5 days, to form the abscopal tumor, 1 × 10^6^ CT26 tumor cells were subcutaneously injected into the left flank. When the primary tumor volume reached about 100 mm^3^, mice were randomly divided into five groups, and intratumorally injected with 100 µL of PBS, OMVs, vOMVs, siPD-L1@OMVs or siPD-L1@vOMVs (vesicle dose: 10 μg per mouse; siPD-L1 dose: 4.3 μg per mouse) every 2 days for two times. On the 7th day after the final administration, both primary and abscopal tumors were isolated and cut into small pieces, and then homogenized in cold PBS to form the single-cell suspension. For T lymphocyte analysis, cells from tumors were stained with fluorescent antibodies of CD3, CD4, and CD8. Then, stained cells from tumors were washed with PBS three times and measured by flow cytometer.

**1.17. Antigen-specific immune responses**

BALB/c mice were subcutaneously incubated with CT26 tumor cells (1 × 10^6^). When tumors reached about 100 mm^3^, mice were randomly divided into five groups, and intratumorally injected with 100 µL of PBS, OMVs, vOMVs, siPD-L1@OMVs or siPD-L1@vOMVs (vesicle dose: 10 μg per mouse; siPD-L1 dose: 4.3 μg per mouse) every 2 days for two times. After one week, the splenocytes from the immunized mice were collected and restimulated ex vivo with CT26 antigens (obtained by multigelation) for 24 h. Next, the expression of IFN-γ in CD3^+^ CD8^+^ T cells was measured by flow cytometry.

**1.18. Evaluation of the metastasis**

To construct the lung metastasis model, BALB/c mice were subcutaneously incubated with 4T1 tumor cells (1 × 10^6^). When tumors reached about 100 mm^3^, mice were randomly divided into five groups and intratumorally injected with 100 µL of PBS, OMVs, vOMVs, siPD-L1@OMVs or siPD-L1@vOMVs (vesicle dose: 10 μg per mouse; siPD-L1 dose: 4.3 μg per mouse) every 2 days for two times. On the 2nd day after the final administration, the mice were intravenously injected with 4T1 cells (3 × 10^5^). After another 15 days, the mice were killed, and their lungs were excised. Lung metastasis nodules were then manually counted and lung tissue sections were subjected to H&E staining. On day 42, splenocytes were isolated and the percentage of memory T cells (CD3^+^CD8^+^CD44^+^CD62L^−^) was detected by flow cytometry.

**1.19. Statistical analysis**

Data were reported as mean ± SD. An unpaired two-tailed Student t-test was used to analyze the statistically significant differences and data were considered statistically significant when the values of P <0.05. *P < 0.05, **P < 0.01, ***P < 0.001, and ****P < 0.0001. n.s., not significant. Image Lab (version number, 3.0) was used to analyze the data of western blot. FlowJo (version number, 10.0.0.0) was used to analyze the data of flow cytometry. GraphPad Prism (version number, 8.0.2.263) was used for the statistical analysis. Living Image software (version number, 4.3.1.16427) was used to analyze the data of *in vivo* bioluminescence assay.

**Table S1.** Representative proteins identified from vOMVs detected by proteomic analysis.

| Index | Accession | Description | Area | Proportion (%) | |
| --- | --- | --- | --- | --- | --- |
| Cell outer membrane | | | | |  |
| 1 | P02943 | LAMB | 11778000000 | 20.38716956 | |
| 2 | P0A910 | OMPA | 4938900000 | 8.549005921 | |
| 3 | P02930 | TOLC | 982010000 | 1.699813583 | |
| 4 | P0A917 | OMPX | 755370000 | 1.307510296 | |
| 5 | P0A908 | MIPA | 739650000 | 1.280299708 | |
| 6 | P13036 | FECA | 653160000 | 1.130589546 | |
| 7 | P0A912 | PAL | 572050000 | 0.990191913 | |
| 8 | P05825 | FEPA | 536350000 | 0.928396875 | |
| 9 | P02931 | OMPF | 520820000 | 0.901515168 | |
| 10 | P39325 | YTFQ | 478310000 | 0.827932338 | |
| Cytoplasm | | | | |  |
| 1 | P0A6F5 | CH60 | 987660000 | 1.70959347 | |
| 2 | P0A8T7 | RPOC | 671740000 | 1.162750661 | |
| 3 | P61889 | MDH | 534200000 | 0.924675325 | |
| 4 | P04391 | OTC1 | 423510000 | 0.733076089 | |
| 5 | P06960 | OTC2 | 423510000 | 0.733076089 | |
| 6 | P0AG67 | RS1 | 419170000 | 0.725563751 | |
| 7 | P25516 | ACNA | 358540000 | 0.620616045 | |
| 8 | P60422 | RL2 | 334910000 | 0.579713615 | |
| 9 | P0A7L3 | RL20 | 306450000 | 0.53045068 | |
| 10 | P16659 | SYP | 304420000 | 0.526936845 | |
| Periplasm | | | | |  |
| 1 | P23843 | OPPA | 1804700000 | 3.123851664 | |
| 2 | P0A855 | TOLB | 1789600000 | 3.097714268 | |
| 3 | P0AEX9 | MALE | 1538300000 | 2.662725669 | |
| 4 | P0AEQ3 | GLNH | 1073900000 | 1.858870894 | |
| 5 | P37902 | GLTI | 859850000 | 1.488360311 | |
| 6 | P08331 | CPDB | 700910000 | 1.213242572 | |
| 7 | P09394 | GLPQ | 540080000 | 0.934853331 | |
| 8 | P30859 | ARTI | 502310000 | 0.86947522 | |
| 9 | P07024 | USHA | 420400000 | 0.727692824 | |
| 10 | P45523 | FKBA | 408810000 | 0.707631074 | |
| Cell inner membrane | | | | |  |
| 1 | P64451 | YDCL | 3732200000 | 6.460264411 | |
| 2 | P0CE47 | EFTU1 | 1635400000 | 2.830801248 | |
| 3 | P0CE48 | EFTU2 | 1626700000 | 2.815741953 | |
| 4 | P0ABB4 | ATPB | 925250000 | 1.601564666 | |
| 5 | P0ABB0 | ATPA | 337860000 | 0.584819928 | |
| 6 | P0ADA3 | NLPD | 168580000 | 0.29180413 | |
| 7 | P0AEZ3 | MIND | 125740000 | 0.217650085 | |
| 8 | P23865 | PRC | 121950000 | 0.211089771 | |
| 9 | P10408 | SECA | 86252000 | 0.149298196 | |
| 10 | P0AC41 | SDHA | 42745000 | 0.073989605 | |
| Unknown | | | | |  |
| 1 | P76177 | YDGH | 274320000 | 0.474835146 | |
| 2 | P23538 | PPSA | 165280000 | 0.286091984 | |
| 3 | P0ACY3 | YEAG | 163470000 | 0.282958958 | |
| 4 | P77554 | YAHJ | 62075000 | 0.107448934 | |
| 5 | P06993 | MALT | 50268000 | 0.087011567 | |
| 6 | P0AF70 | YJEI | 7409500 | 0.012825499 | |
| 7 | P00722 | BGAL | 4326800 | 0.007489489 | |
| 8 | P52644 | HSLJ | 2283600 | 0.003952805 | |
| 9 | P77161 | GLXR | 247640 | 0.000428653 | |
| Nucleoid protein | | | | |  |
| 1 | P0A6X7 | IHFA | 2190700 | 0.003792 | |

**Table S2.** Representative bacterial proteins on the CT26 cell surface after vOMVs treatment detected by proteomic analysis.

| Index | Accession | Description | Area | Proportion (%) | |
| --- | --- | --- | --- | --- | --- |
| Cell outer membrane | | | | |  |
| 1 | P02943 | LAMB | 257530000 | 1.361872741 | |
| 2 | P69776 | LPP | 202000000 | 1.068218435 | |
| 3 | P0A910 | OMPA | 191360000 | 1.01195188 | |
| 4 | P0A908 | MIPA | 38041000 | 0.201168799 | |
| 5 | P0A912 | PAL | 21286000 | 0.11256484 | |
| Cytoplasm | | | | |  |
| 1 | P0A8V2 | RPOB | 613680000 | 3.245268759 | |
| 2 | P0A7Z4 | RPOA | 497810000 | 2.632523858 | |
| 3 | P0A6F5 | CH60 | 434550000 | 2.297991688 | |
| 4 | P0A8T7 | RPOC | 388490000 | 2.054416732 | |
| 5 | P63284 | CLPB | 301720000 | 1.595558744 | |
| Periplasm | | | | |  |
| 1 | P0AEX9 | MALE | 383010000 | 2.025437341 | |
| 2 | P0AB24 | EFEO | 75941000 | 0.401591961 | |
| 3 | P45523 | FKBA | 55006000 | 0.290883283 | |
| 4 | P0A855 | TOLB | 45720000 | 0.241776965 | |
| 5 | P0AEQ3 | GLNH | 37469000 | 0.198143943 | |
| Cell inner membrane | | | | |  |
| 1 | P0ABB4 | ATPB | 374330000 | 1.979535678 | |
| 2 | P0ABB0 | ATPA | 229960000 | 1.216076789 | |
| 3 | P0ADB7 | ECNB | 54309000 | 0.287197401 | |
| 4 | P0AEZ3 | MIND | 43120000 | 0.228027618 | |
| Unknown | | | | |  |
| 1 | P23538 | PPSA | 162220000 | 0.857853438 | |
| 2 | P76177 | YDGH | 105860000 | 0.559809919 | |

**Table S3.** Depth TCR repertoire analysis of Shannon.Index, Invsimpson.Index, Simpson.Index, Pielous, Singleton, DE50, and Convergence of T cells in tumor-draining lymph nodes (TDLNs) from PBS or vOMVs-treated mice bearing CT26 tumor.

|  | Shannon.Index | Invsimpson.Index | Simpson.Index | Pielous | Singleton | DE50 | Convergence |
| --- | --- | --- | --- | --- | --- | --- | --- |
| PBS | 10.540753 | 26362.927468 | 0.999962 | 1.047033 | 0.354704 | 0.234628 | 0.102222 |
| vOMVs | 10.875313 | 30450.095733 | 0.999967 | 0.966545 | 0.406999 | 0.226691 | 0.136650 |


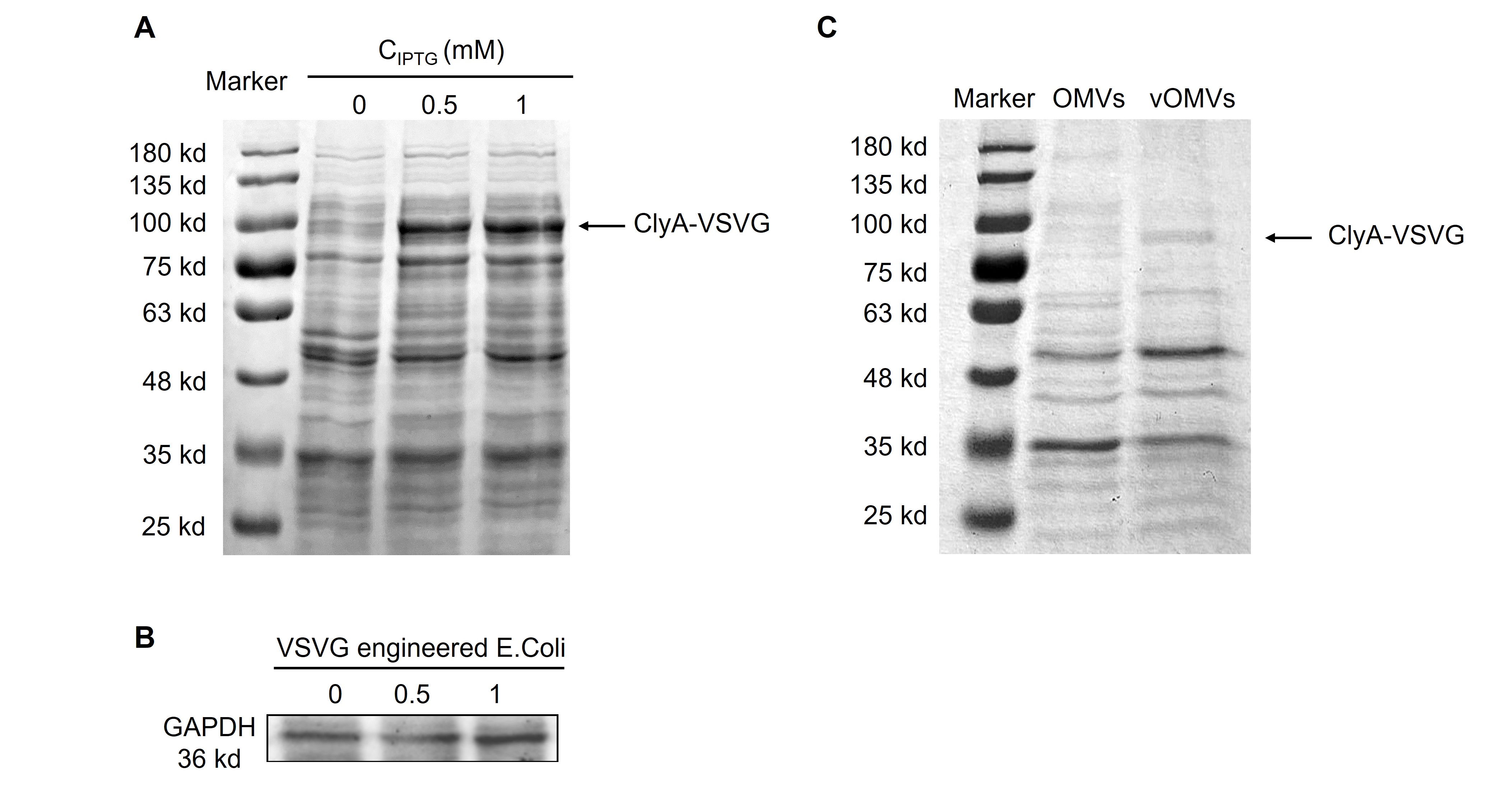


**Figure S1.** Expression of fusion protein ClyA-VSVG on engineered *E. coli* (**A**) and the derived vOMVs (**C**) verified by SDS-PAGE. **B**) Western blot analysis of GAPDH as loading control in *E. coli.*


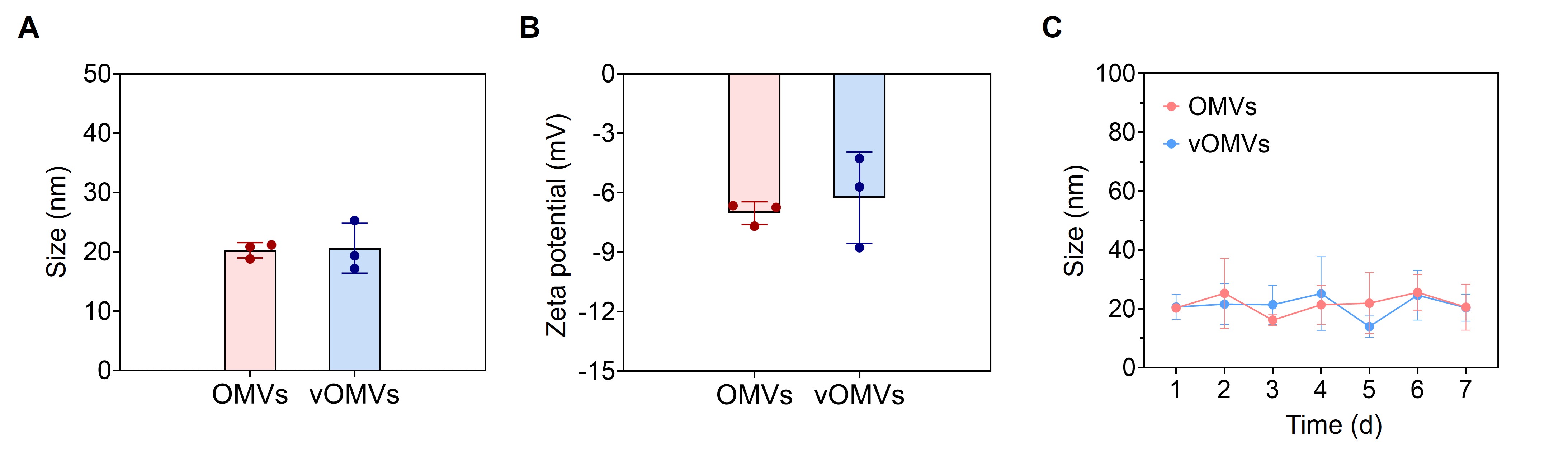


**Figure S2.** Size (**A**) and zeta potential (**B**) of OMVs or vOMVs. **C**) Stability of OMVs or vOMVs in PBS at 4 °C. Data are presented as mean ± s.d. (n = 3 biologically independent samples).


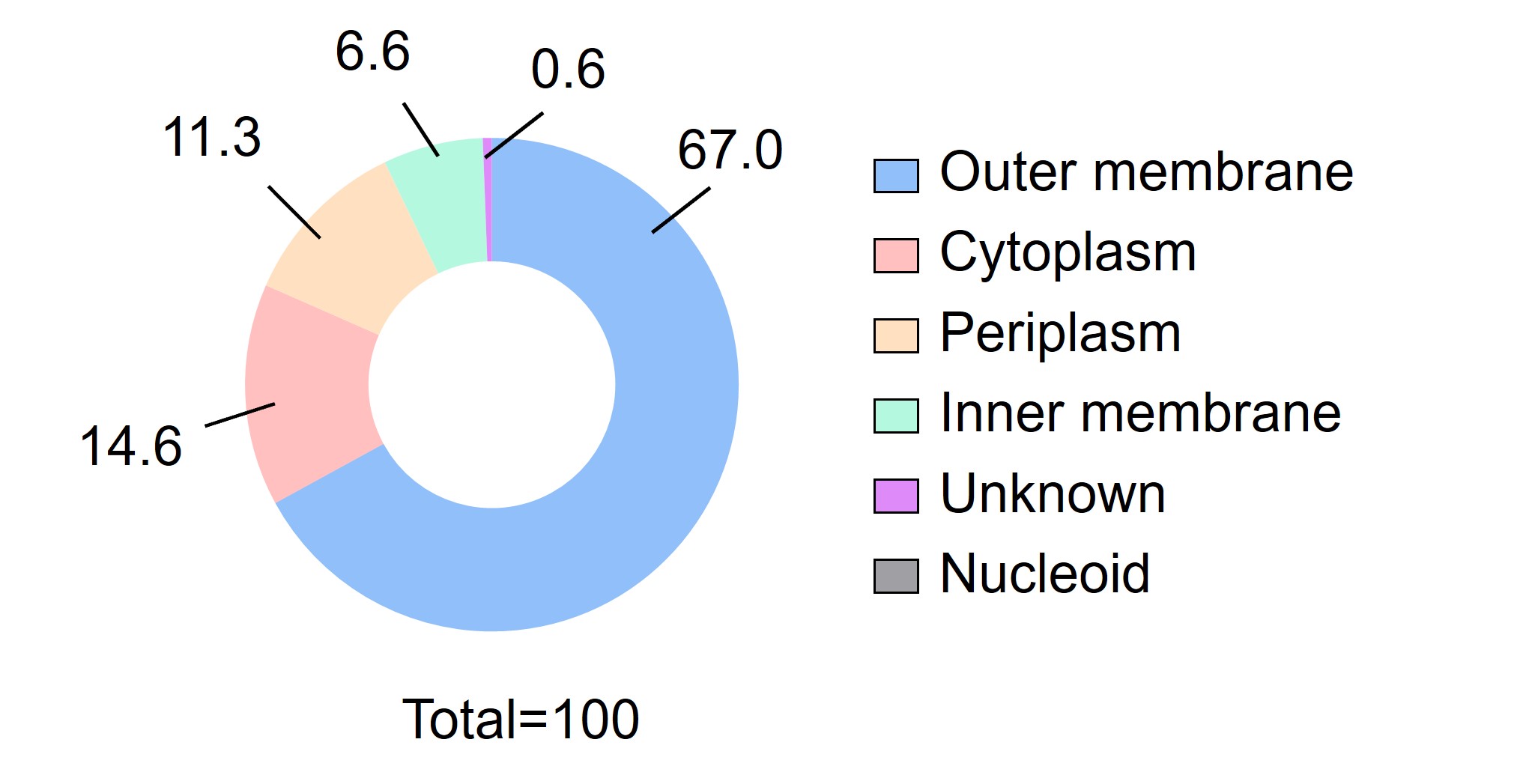


**Figure S3.** Pie chart for the subcellular localization of the expressed proteins in OMVs detected by proteomic analysis.


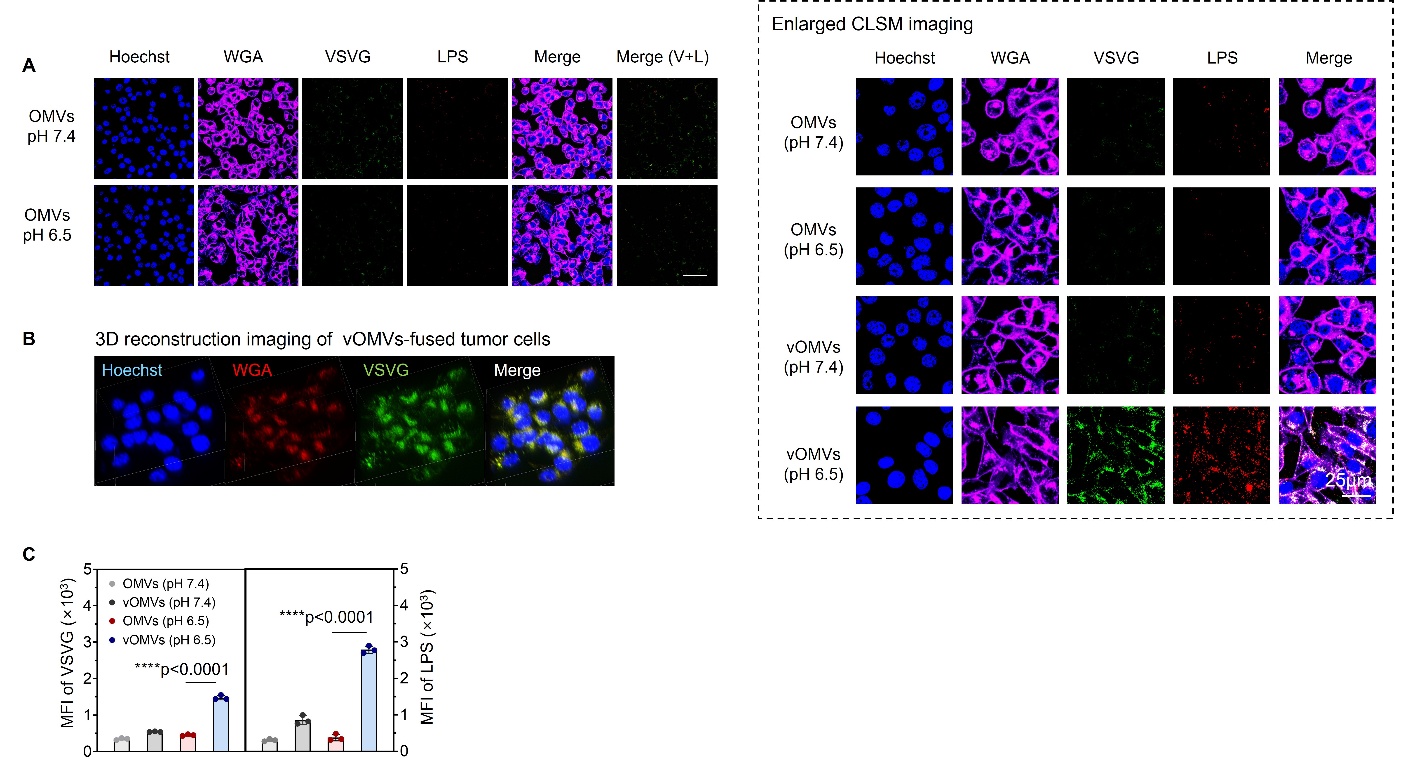


**Figure S4. A**) CLSM imaging of the CT26 tumor cells treated by OMVs. Cell membrane (iFluor®555-conjugated WGA labeled, pink); VSVG (FITC-conjugated anti-VSVG labeled, green); LPS (Cy5-conjugated anti-LPS labeled, red); Cell nucleus (Hoechst 33342, blue). Scale bar: 50 μm. Enlarged CLSM images of tumor cells with different treatments were shown within the dashed box. **B**) 3D reconstruction imaging of vOMVs-fused tumor cells. Cell membrane (iFluor®555-conjugated WGA labeled, red); VSVG (FITC-conjugated anti-VSVG labeled, green); Cell nucleus (Hoechst 33342, blue). Scale bar: 25 μm. **C)** Quantification of the mean fluorescence intensities (MFI) of VSVG and LPS on tumor cells in each group. Data are presented as mean ± s.d. (n = 3 biologically independent samples).


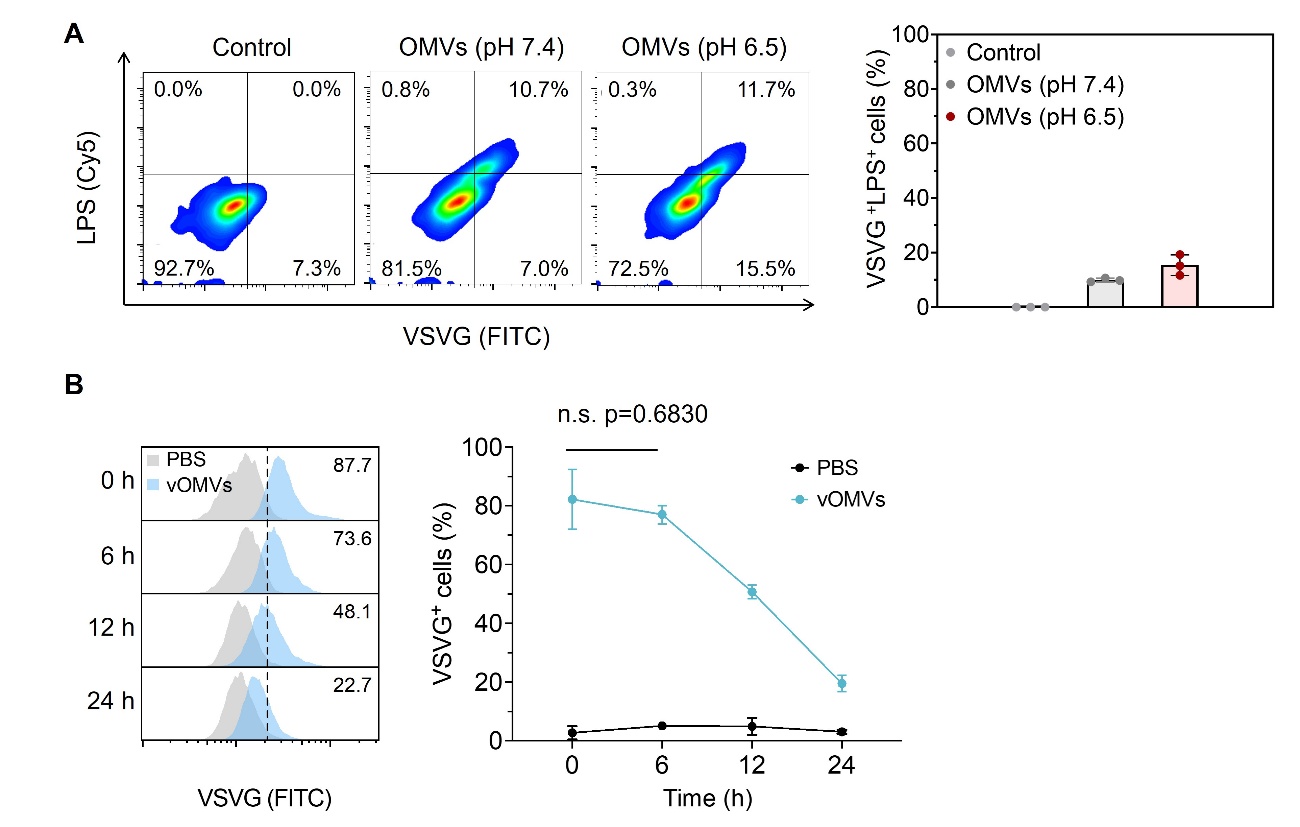


**Figure S5. A)** Flow cytometry and quantification of VSVG^+^LPS^+^ tumor cells after different treatments. **B)** Representative flow cytometry plots and quantification of VSVG^+^ CT26 cells at different time points. Data are presented as mean ± s.d. (n = 3). Statistically significant differences between groups were identified by one-way ANOVA. ****P < 0.0001, ***P < 0.001, **P < 0.01, *P < 0.05.


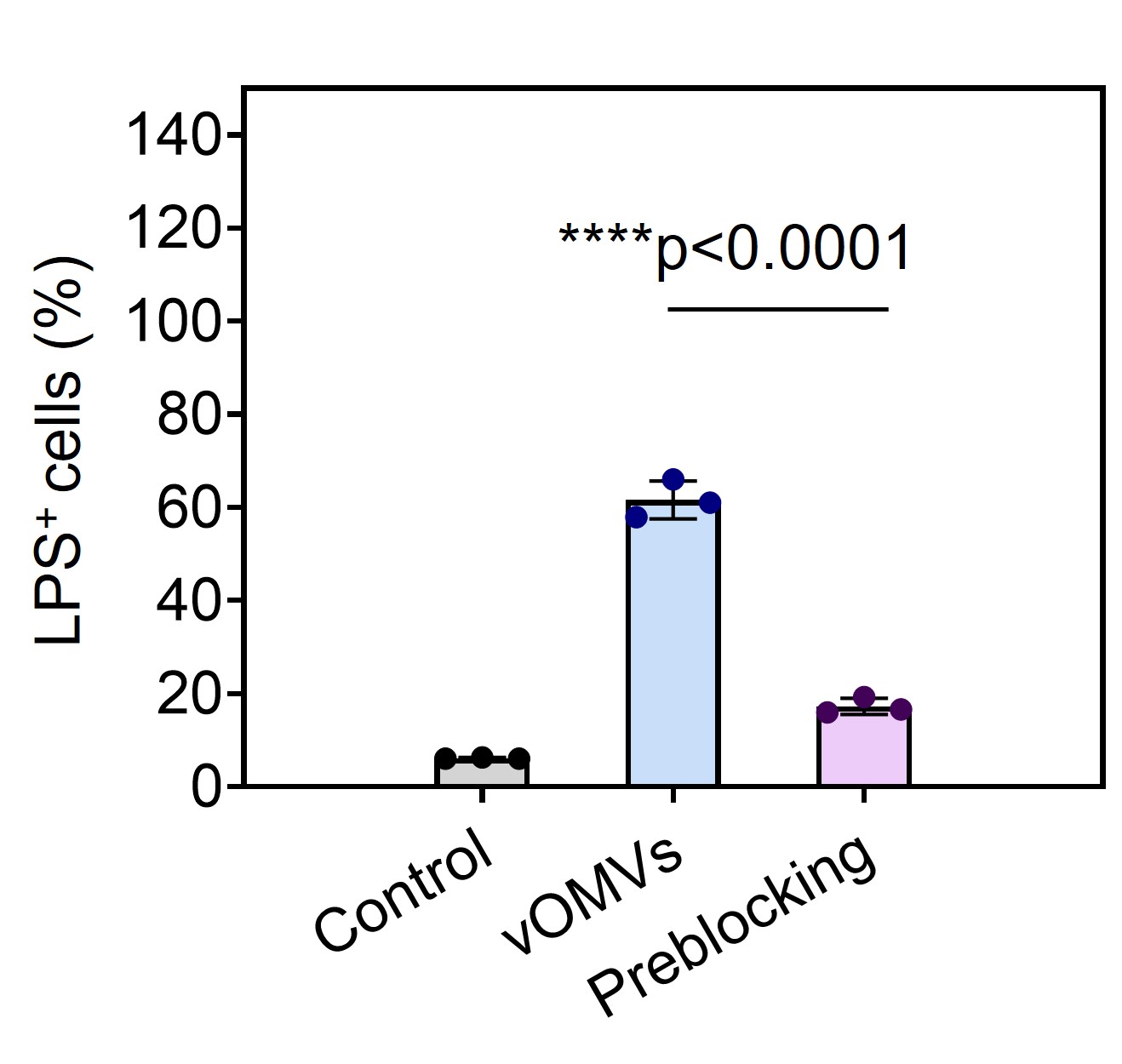


**Figure S6.** Quantification of LPS^+^ tumor cells after different treatments detected by flow cytometry. Data are presented as mean ± s.d. (n = 3 biologically independent samples).


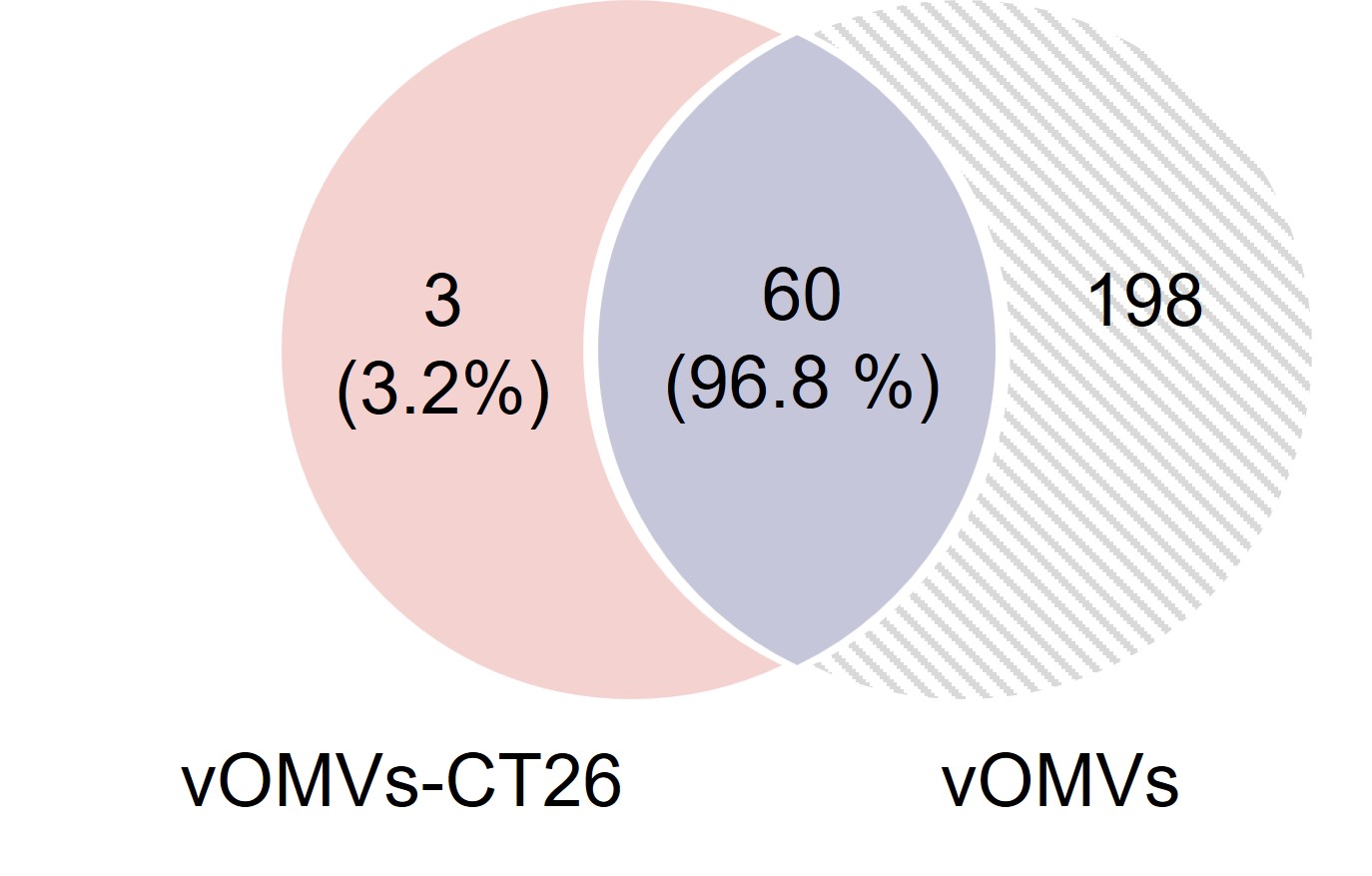


**Figure S7.** Venn diagram of the proteins from vOMVs-fused CT26 cells (vOMVs-CT26) identified by proteomic analysis.


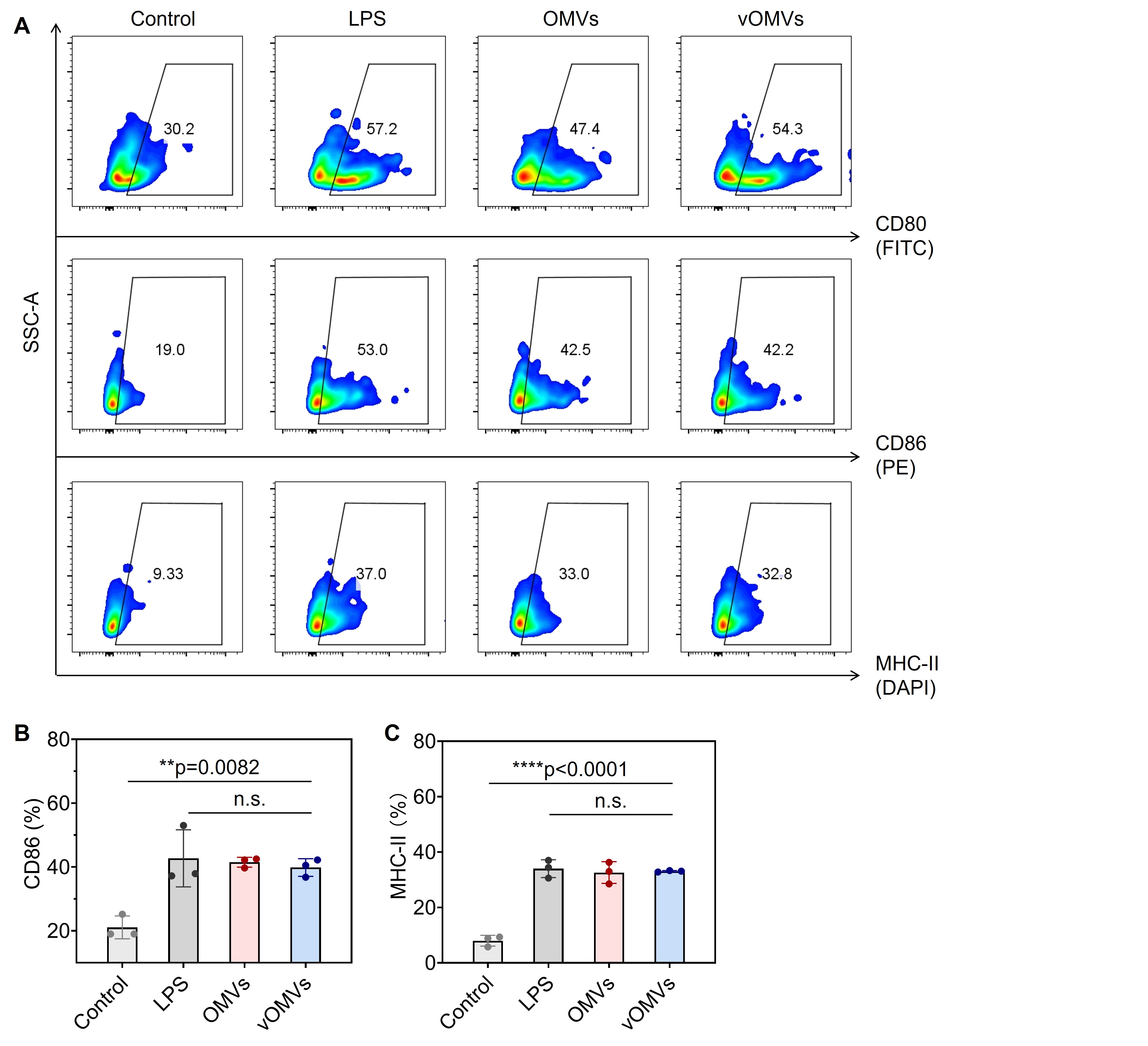


**Figure S8. A**) Representative flow cytometry plots of CD80^+^, CD86^+^, or MHC-II^+^ cells in BMDMs after different treatments. Expression levels of CD86 (**B**) and MHC-II (**C**) on BMDMs analyzed by flow cytometry. LPS (1 μg/mL) is a positive control. Data are presented as mean ± s.d. (n = 3 biologically independent samples).

**
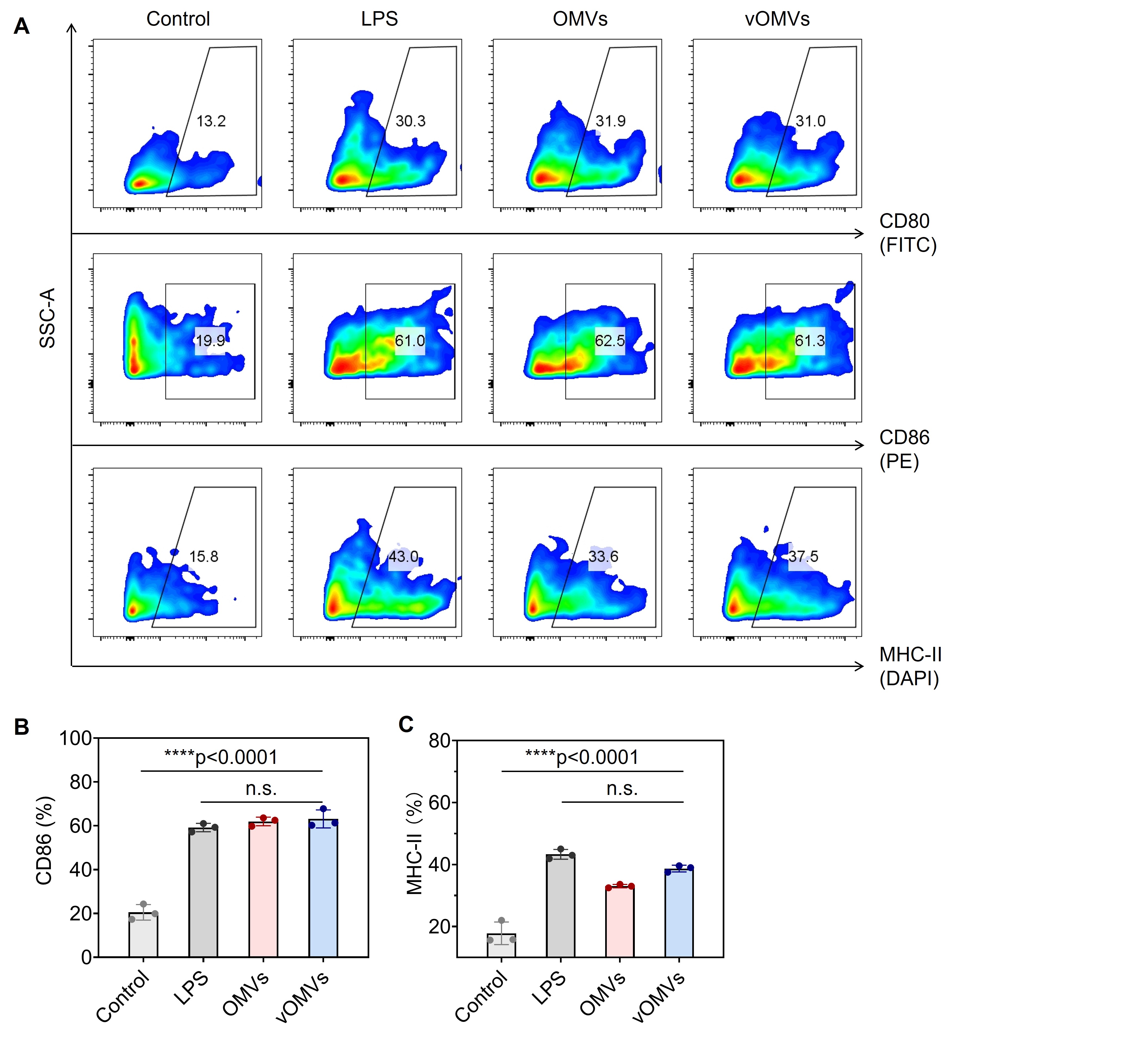
**

**Figure S9. A**) Representative flow cytometry plots of CD80^+^, CD86^+^, or MHC-II^+^ cells in BMDCs after different treatments. Expression levels of CD86 (**B**) and MHC-II (**C**) on BMDCs analyzed by flow cytometry. LPS (1 μg/mL) is a positive control. Data are presented as mean ± s.d. (n = 3 biologically independent samples).


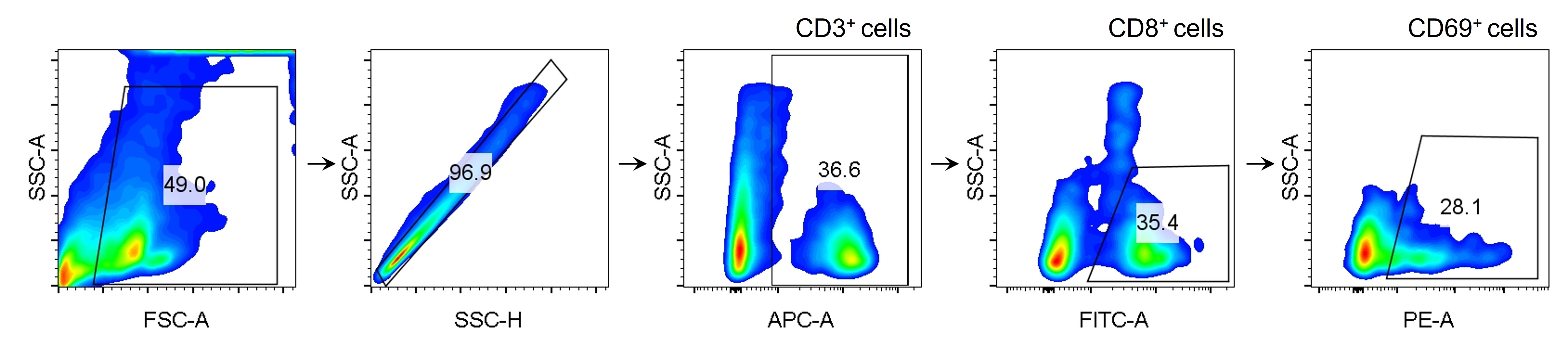


**Figure S10.** Gating strategy for flow cytometry analysis of CD69^+^ T cells (gated on CD3^+^CD8^+^ cells).

**
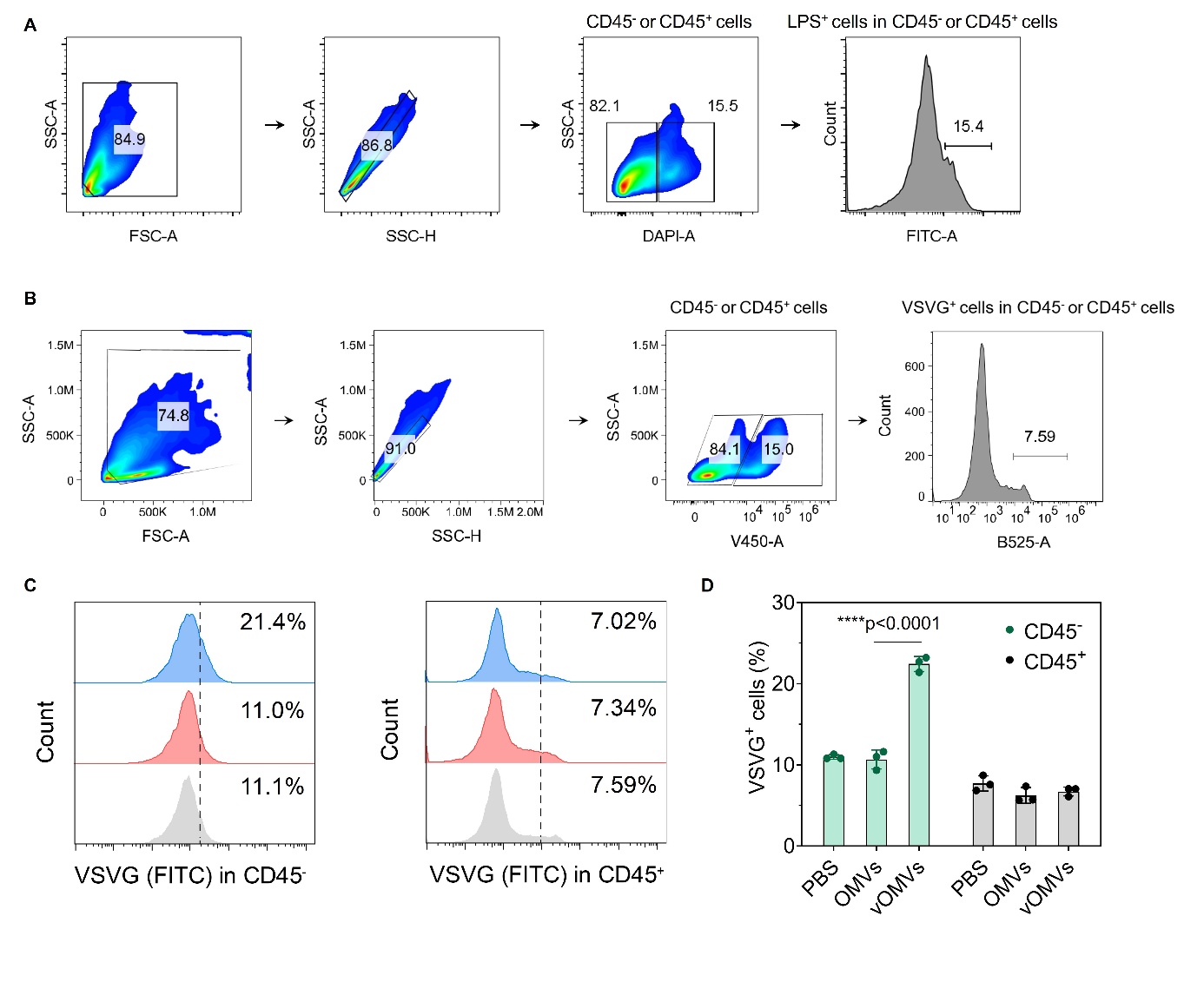
**

**Figure S11. A)** Gating strategy for flow cytometry analysis of LPS^+^ cells gated on CD45^-^ tumor cells or CD45^+^ immune cells. **B)** Gating strategy for flow cytometry analysis of VSVG^+^ cells gated on CD45^-^ tumor cells or CD45^+^ immune cells. **C)** Flow cytometry of VSVG^+^ tumor cells gated on CD45^-^ or CD45^+^ cells. D) Quantification of VSVG^+^ tumor cells gated on CD45^+^ or CD45^-^ cells. Data are presented as mean ± s.d. (n = 3). Statistically significant differences between groups were identified by one-way ANOVA. ****P < 0.0001, ***P < 0.001, **P < 0.01, *P < 0.05.


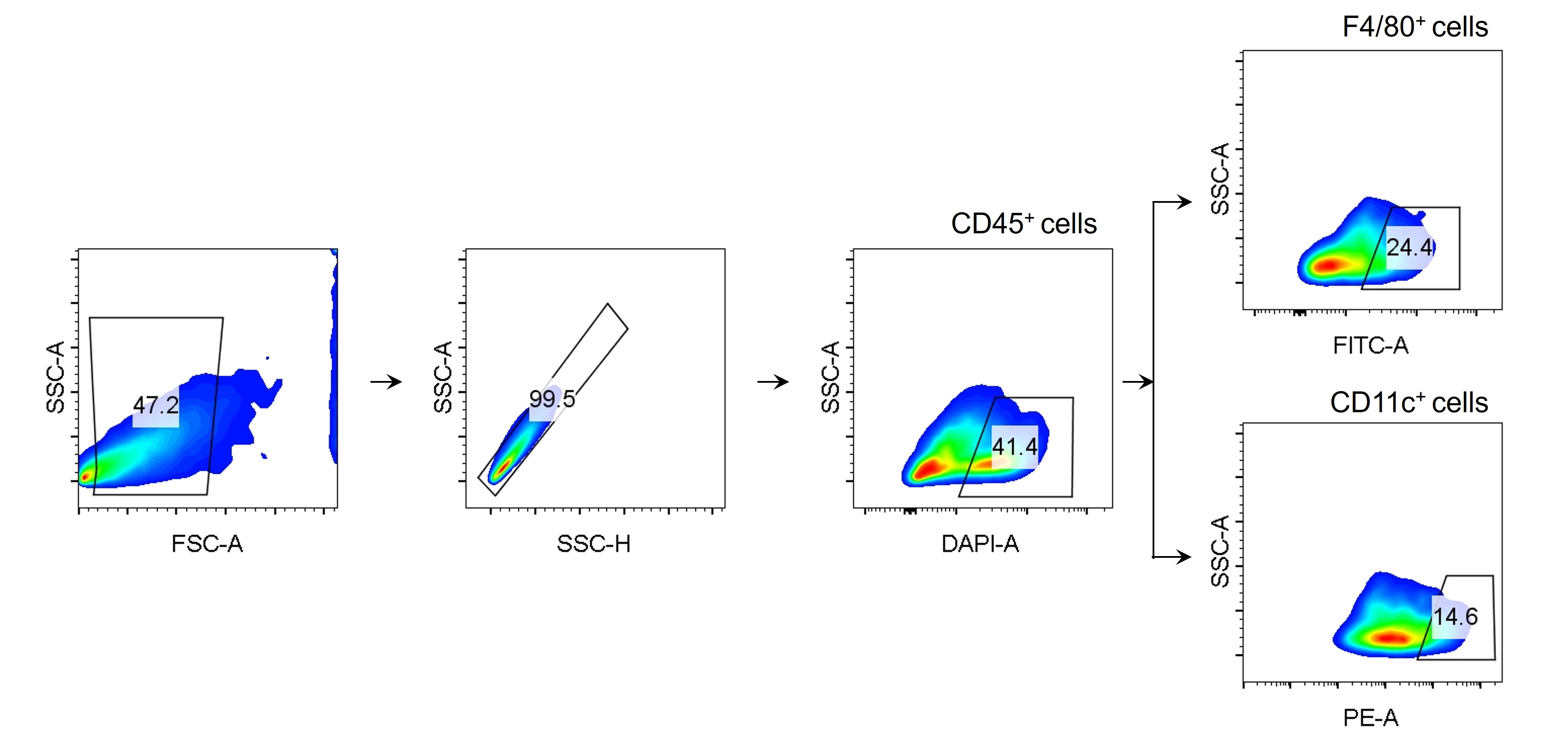


**Figure S12.** Gating strategy for flow cytometry analysis of CD45^+^ immune cells, F4/80^+^ macrophages, and CD11c^+^ DCs in tumor.


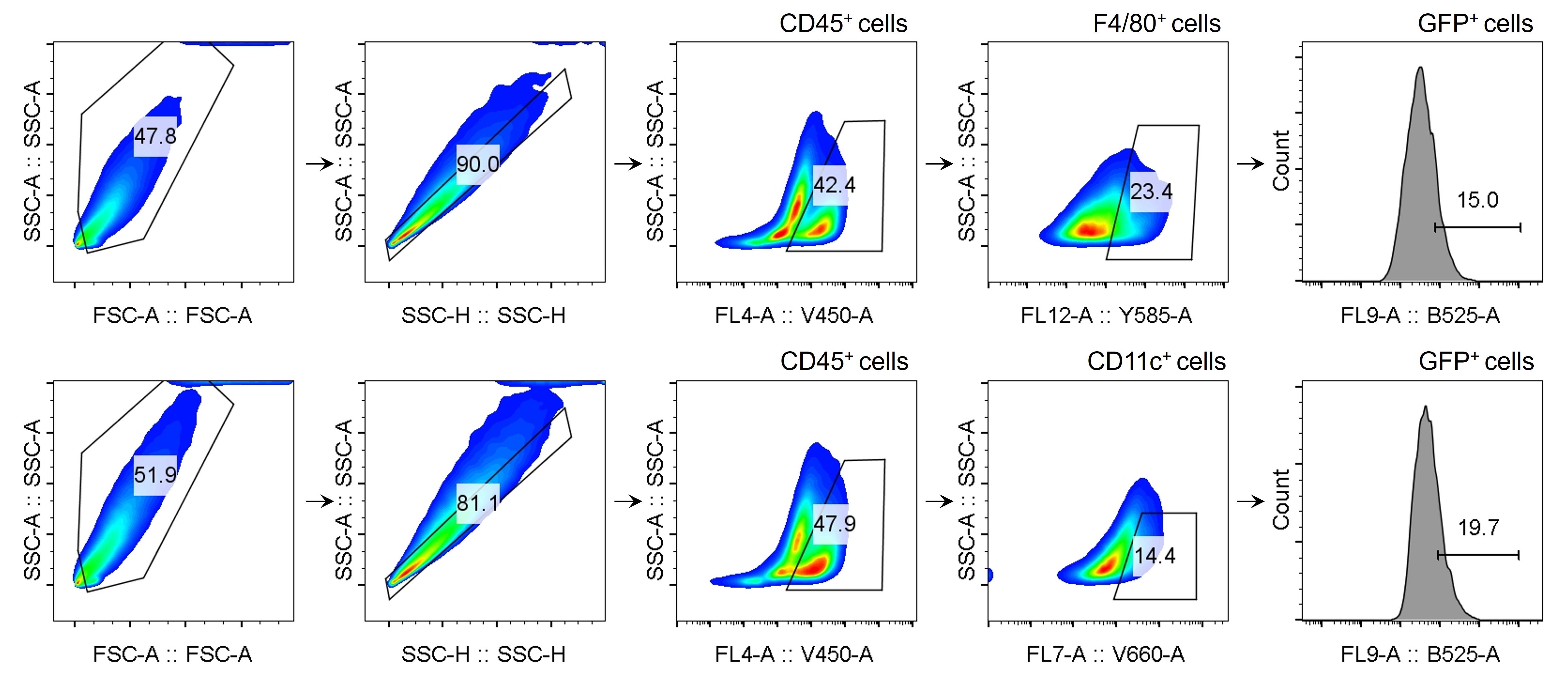


**Figure S13.** Gating strategy for flow cytometry analysis of GFP^+^ cells gated on CD45^+^F4/80^+^ macrophages and CD45^+^CD11c^+^ DCs.


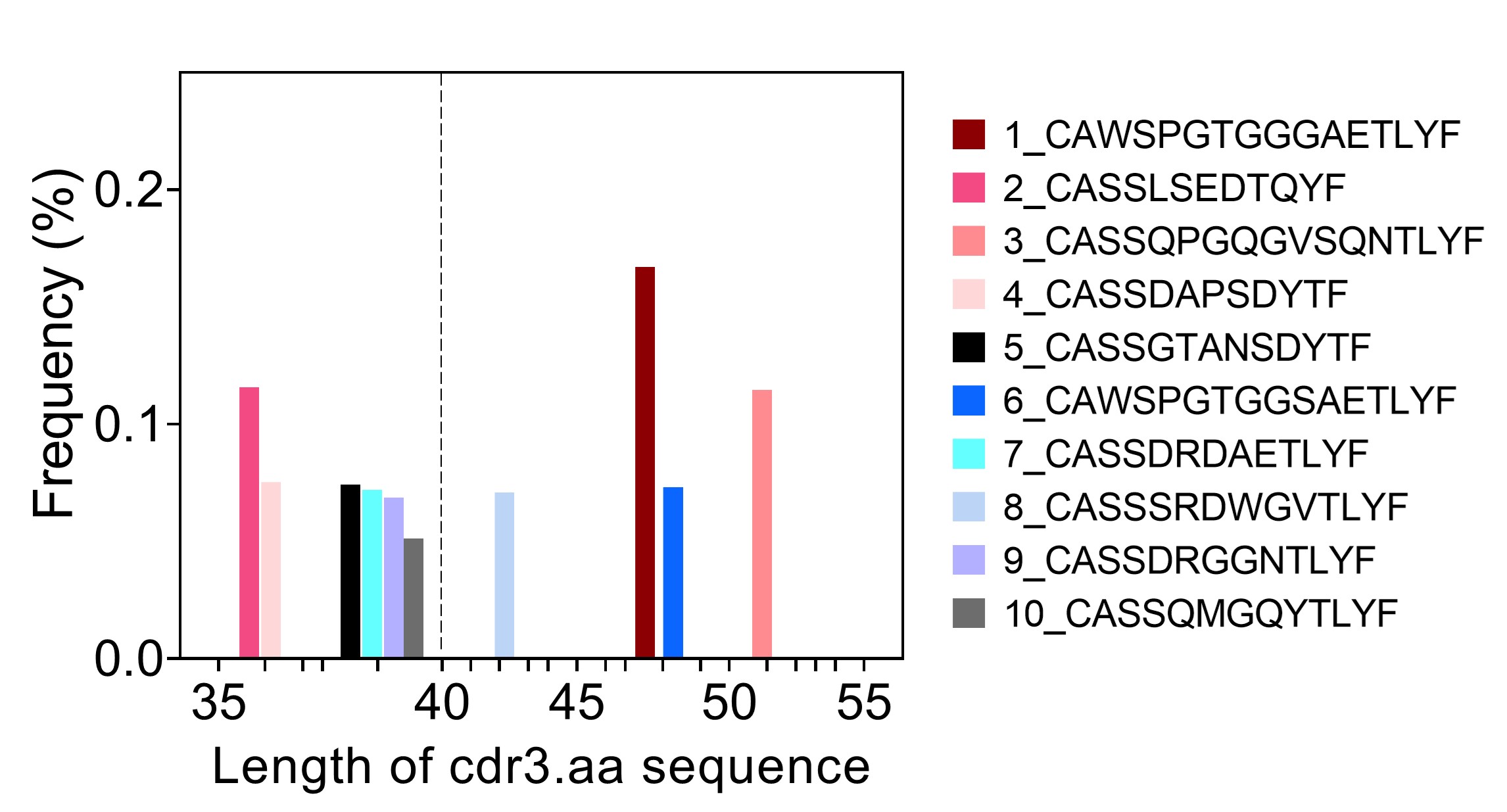


**Figure S14.** Cdr3 length distribution of PBS group (among the top 10 representative cdr3 sequences).

**
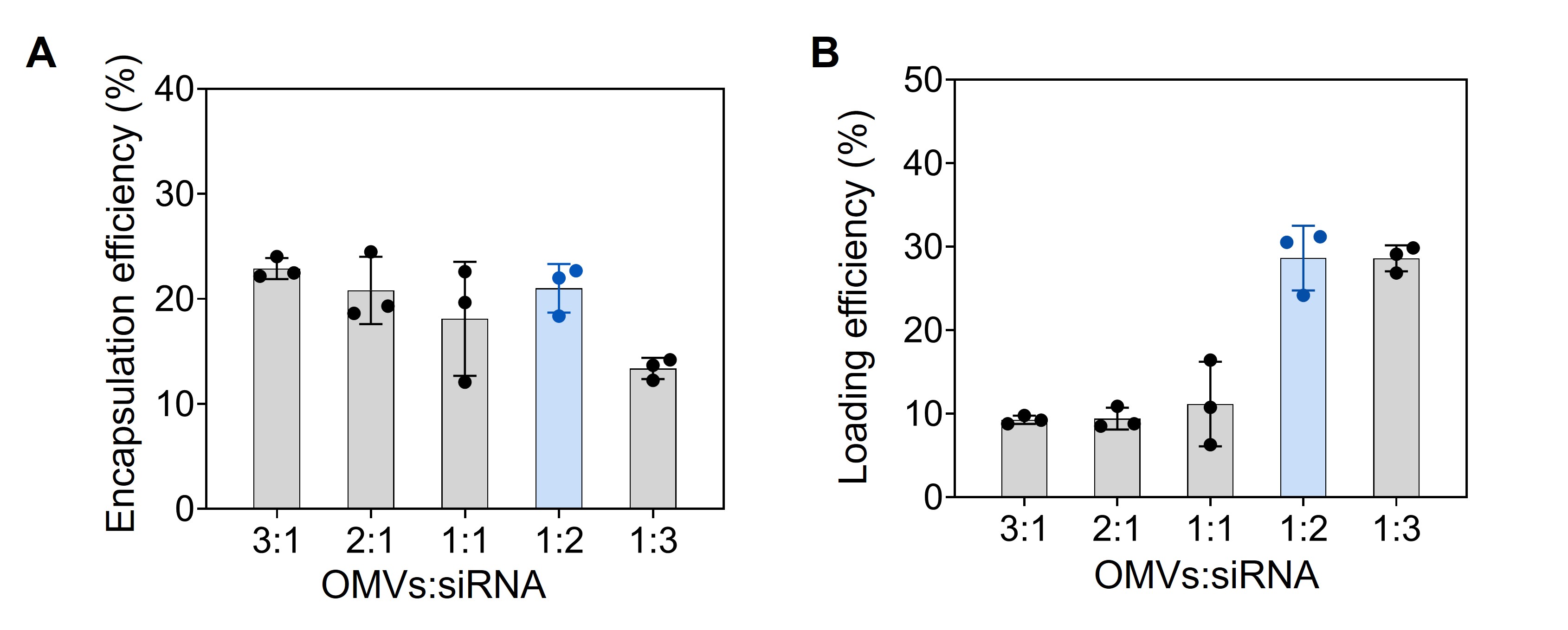
**

**Figure S15.** Encapsulation efficiency (**A**) and loading efficiency (**B**) of siRNA in vOMVs. Different amounts of siRNA were individually incubated with the same amount of vOMVs (10 µg). The loading was saturated at the ratio of 1:2 (vOMVs:siRNA). Data are presented as mean ± s.d. (n = 3 biologically independent samples).

**
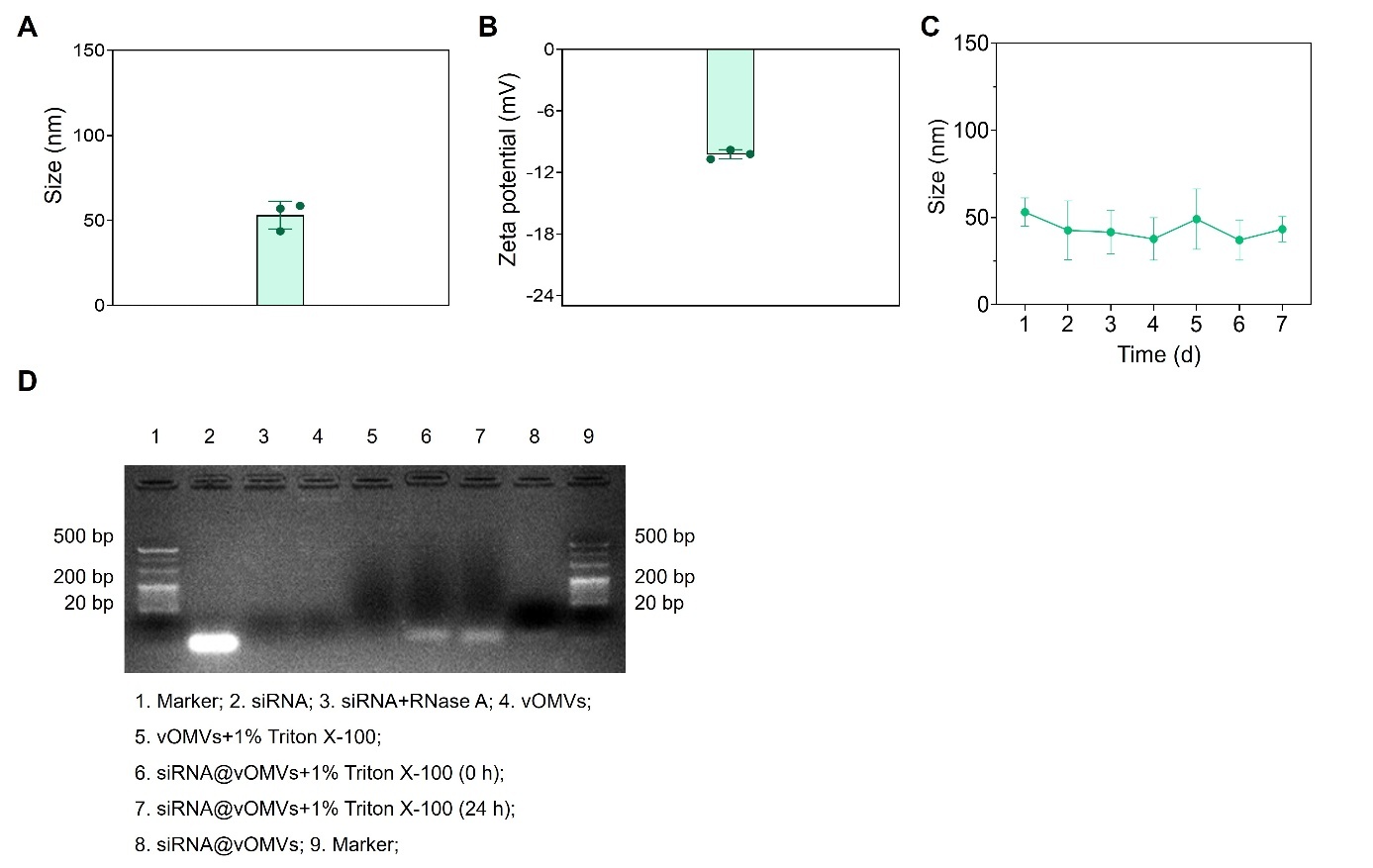
**

**Figure S16.** Size (**A**) and zeta potential (**B**) of siRNA@vOMVs. **C**) Stability of siRNA@vOMVs in PBS at 4 °C. **D)** Agarose gel electrophoresis of siRNA@vOMVs. The intact siRNA exhibited distinct and homogeneous bands (sample 2). However, the exposure to RNase would result in degradation of siRNA, as evidenced by the disappearance of the corresponding bands (sample 3). Triton X-100, a permeabilization detergent, was used for the solubilization of membranes and subsequent release of encapsulated siRNA here. Neither vOMVs (sample 4) nor Triton X-100-treated vOMVs (sample 5) exhibited the band of siRNA. Moreover, no obvious bands were observed in siRNA@vOMVs, demonstrating successful encapsulation of siRNA within the vOMVs (simple 8). Only treated with Triton X-100, which induced the release of siRNA from siRNA@vOMVs, the intact siRNA bands could be observed (simple 6). Furthermore, siRNA@vOMVs that were stored at 4°C for 24 hours retained a consistent band with fresh siRNA@vOMVs, indicating no degradation of the encapsulated siRNA and confirming its stability in vOMVs. Data are presented as mean ± s.d. (n = 3 biologically independent samples).

**
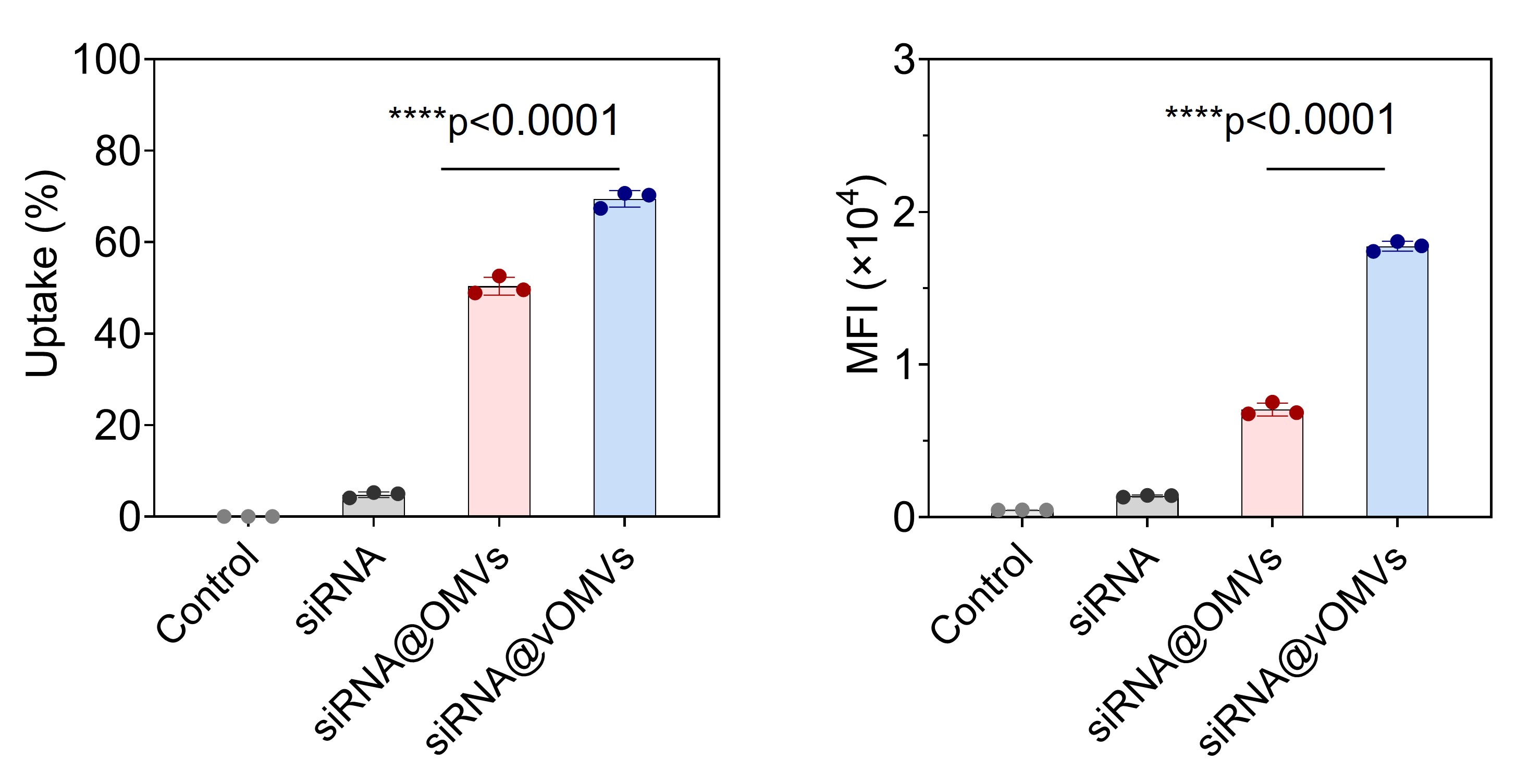
**

**Figure S17.** Quantification of the internalization of Cy3 labeled-siRNA by CT26 cells. Data are presented as mean ± s.d. (n = 3 biologically independent samples).

**
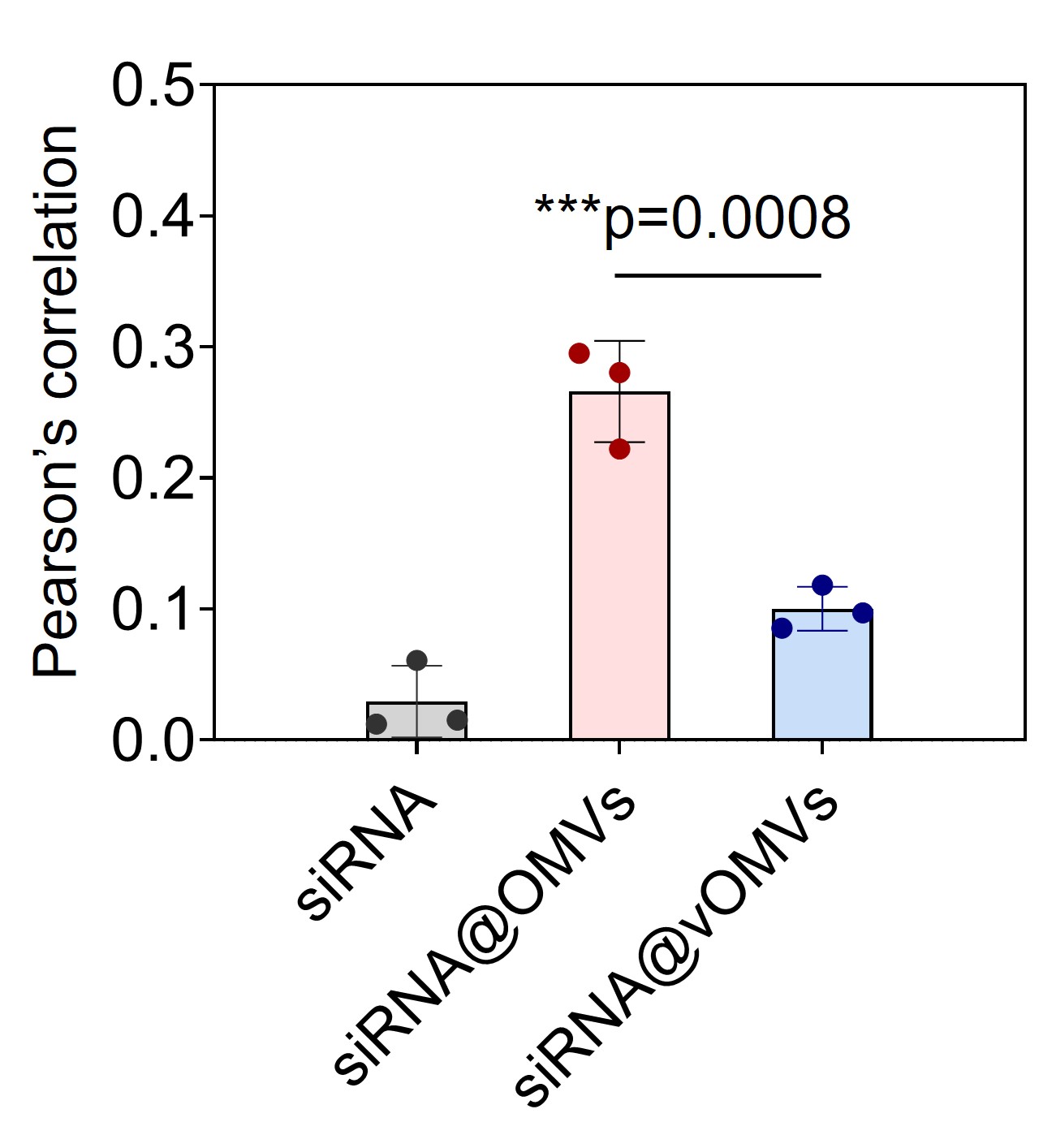
**

**Figure S18.** Colocalization ratio of siRNA and endosome calculated by Pearson’s correlation coefficient. Data are presented as mean ± s.d. (n = 3 biologically independent samples).

**
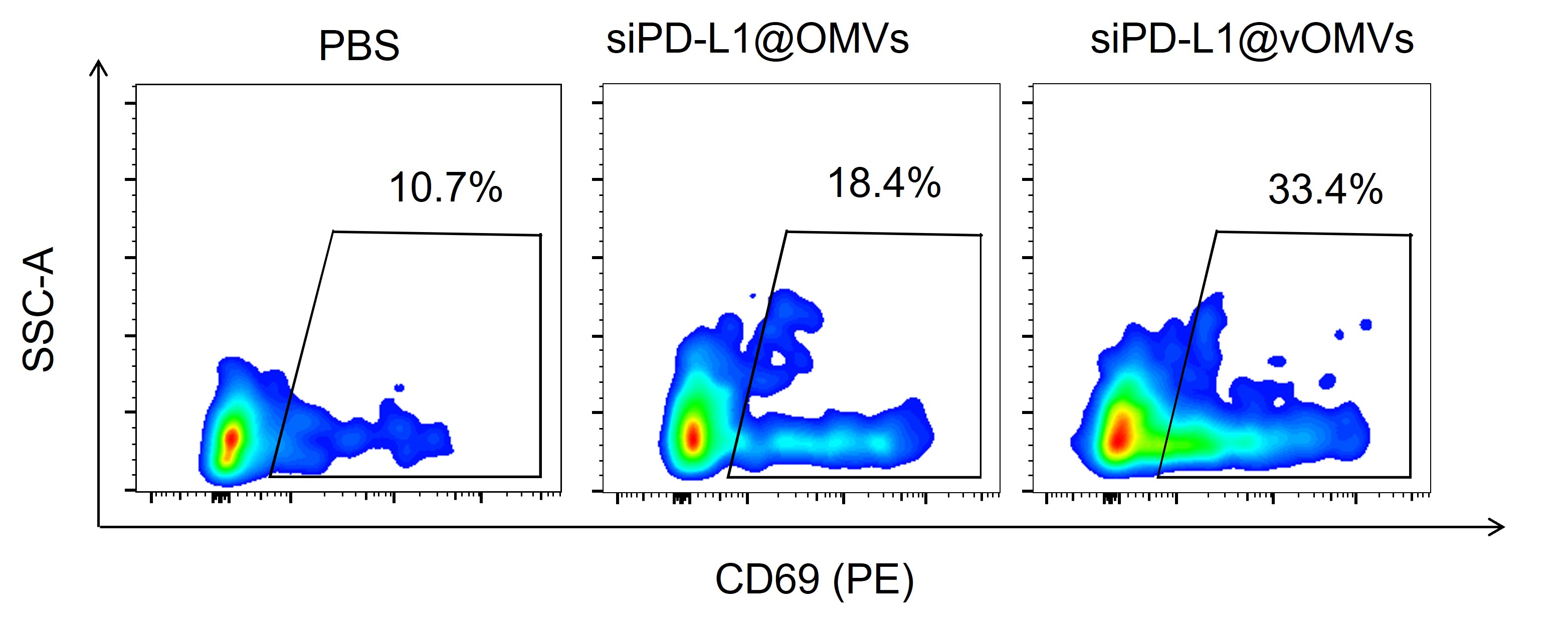
**

**Figure S19.** Flow cytometry of expression levels of CD69 gated on CD8^+^ T cells. The gating strategy was the same as in Figure S10.

**
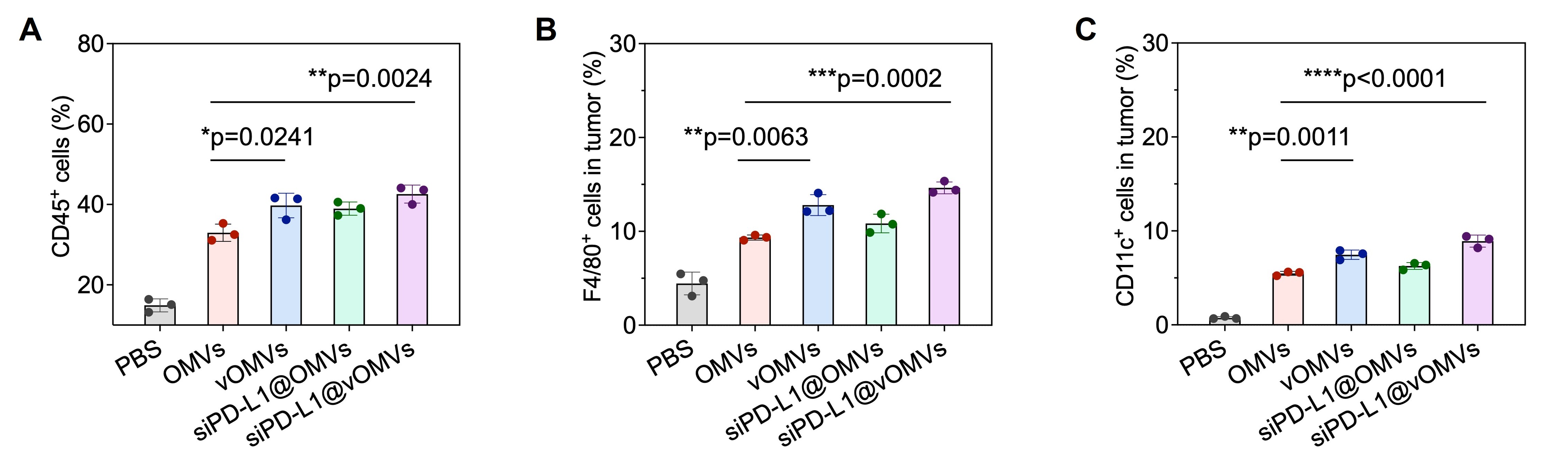
**

**Figure S20.** Flow cytometry of (**A**) tumor-infiltrating CD45^+^ immune cells, (**B**) F4/80^+^ macrophages, and (**C**) CD11c^+^ DCs in tumor after different treatments. Data are presented as mean ± s.d. (n = 3 biologically independent samples).

**
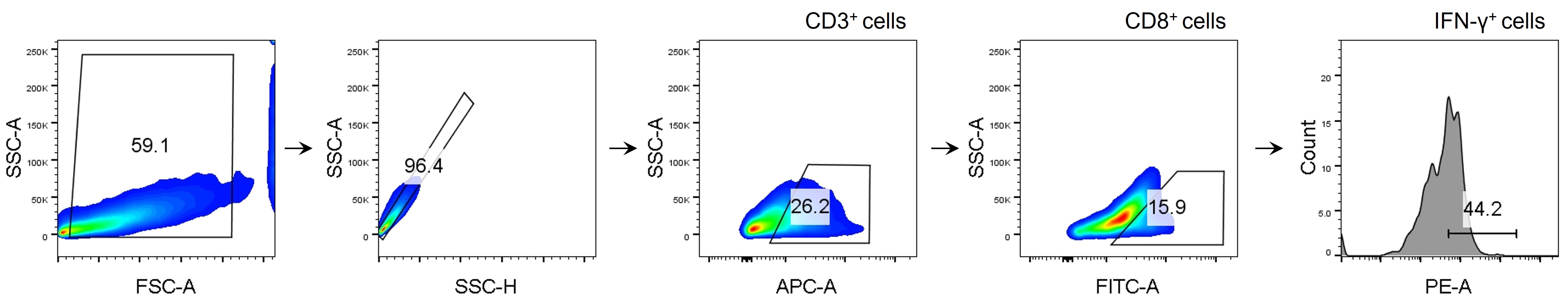
**

**Figure S21.** Gating strategy for flow cytometric studies of IFN-γ^+^ T cells (gated on CD3^+^CD8^+^ T cells) in tumors.

**
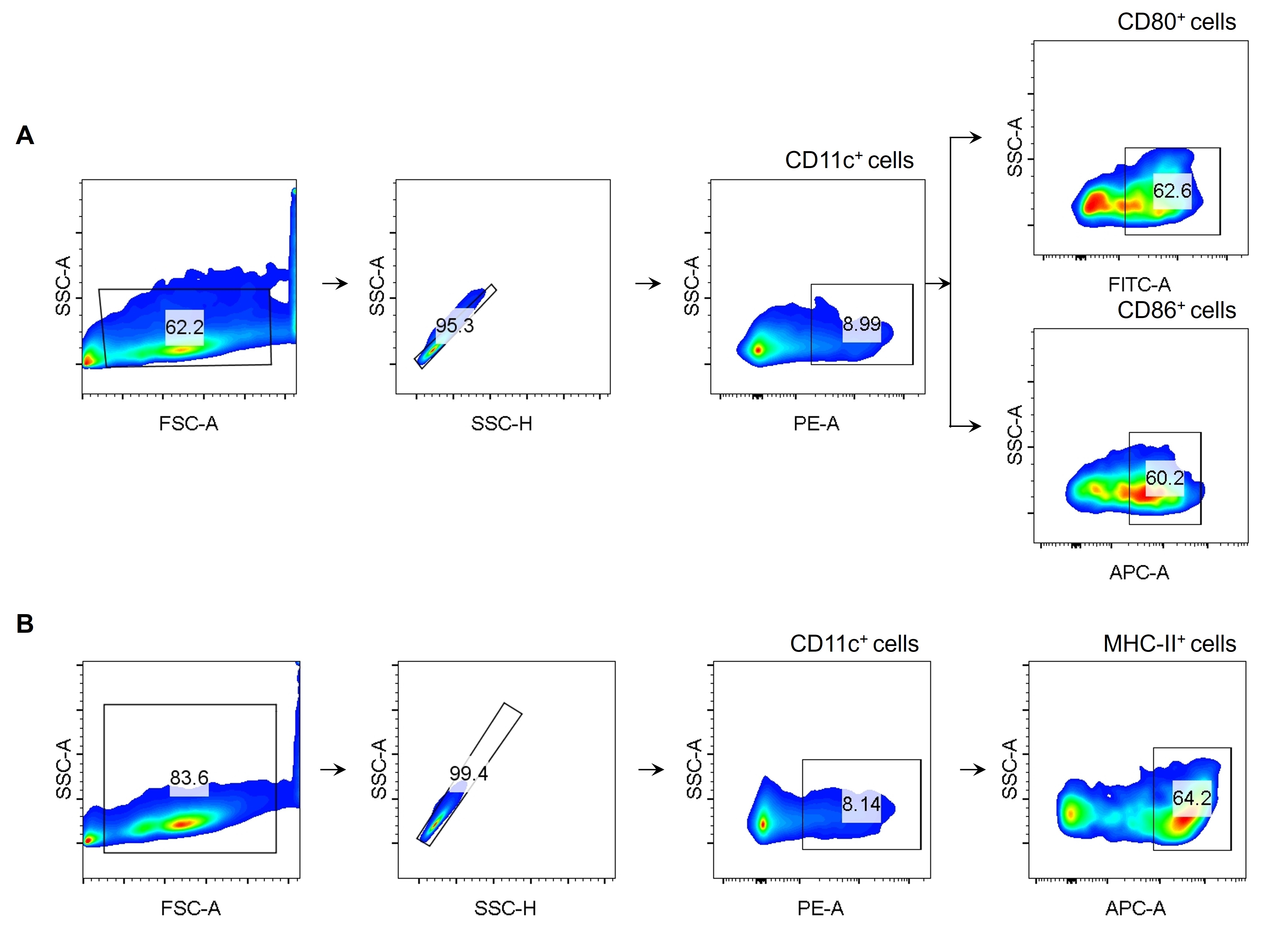
**

**Figure S22.** Gating strategy for flow cytometry analysis of CD80^+^, CD86^+^ **(A),** and MHC^+^ **(B)** gated on CD11c^+^ cells in TDLNs.

**
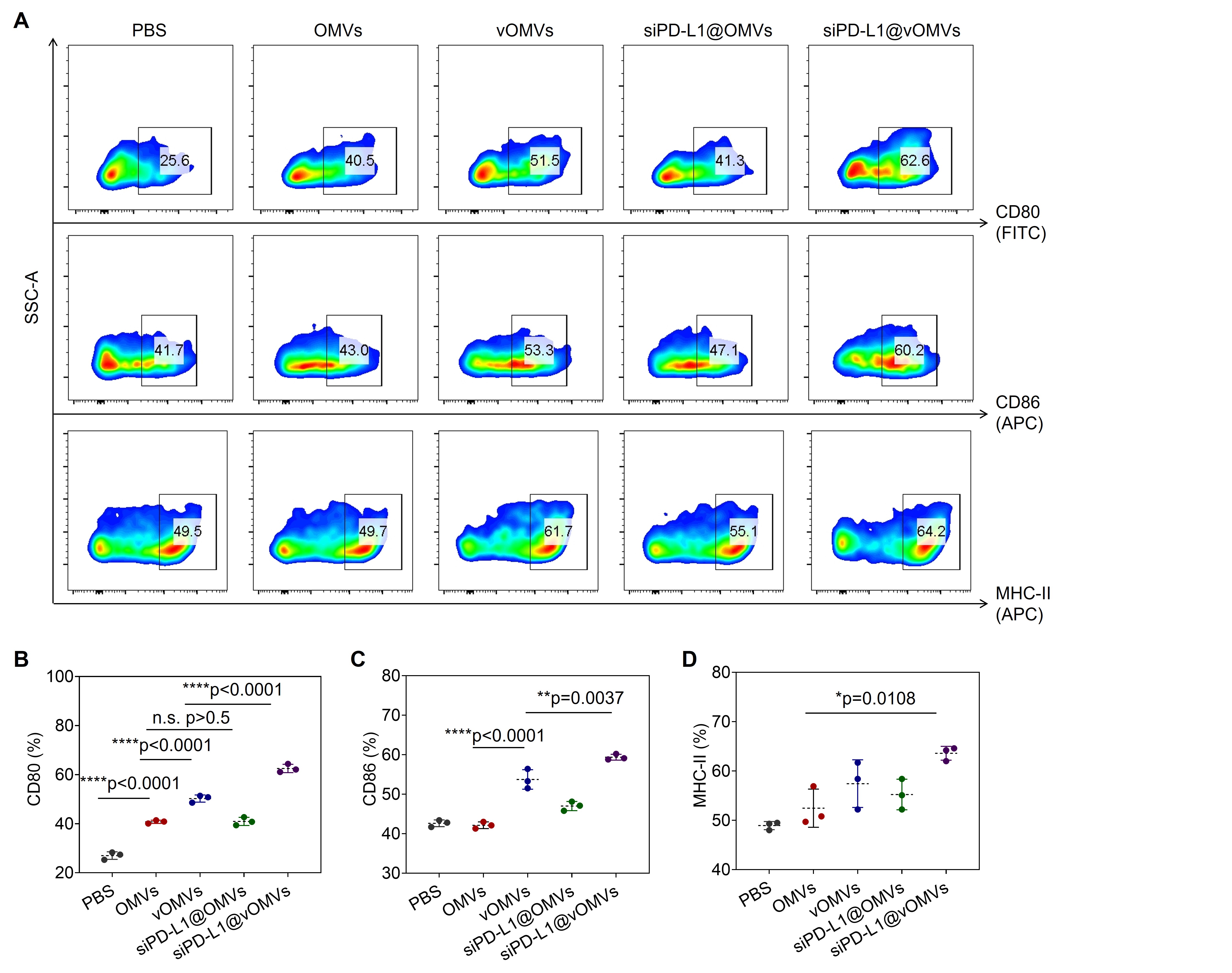
**

**Figure S23. A**) Representative flow cytometry plots of CD80^+^, CD86^+^, or MHC-II^+^ cells gated on CD11c^+^ cells from different treatment groups in TDLNs. **B-D**) Expression levels of CD80, CD86, and MHC-II gated on CD11c^+^ cells in TDLNs. Data are presented as mean ± s.d. (n = 3 biologically independent samples).

**
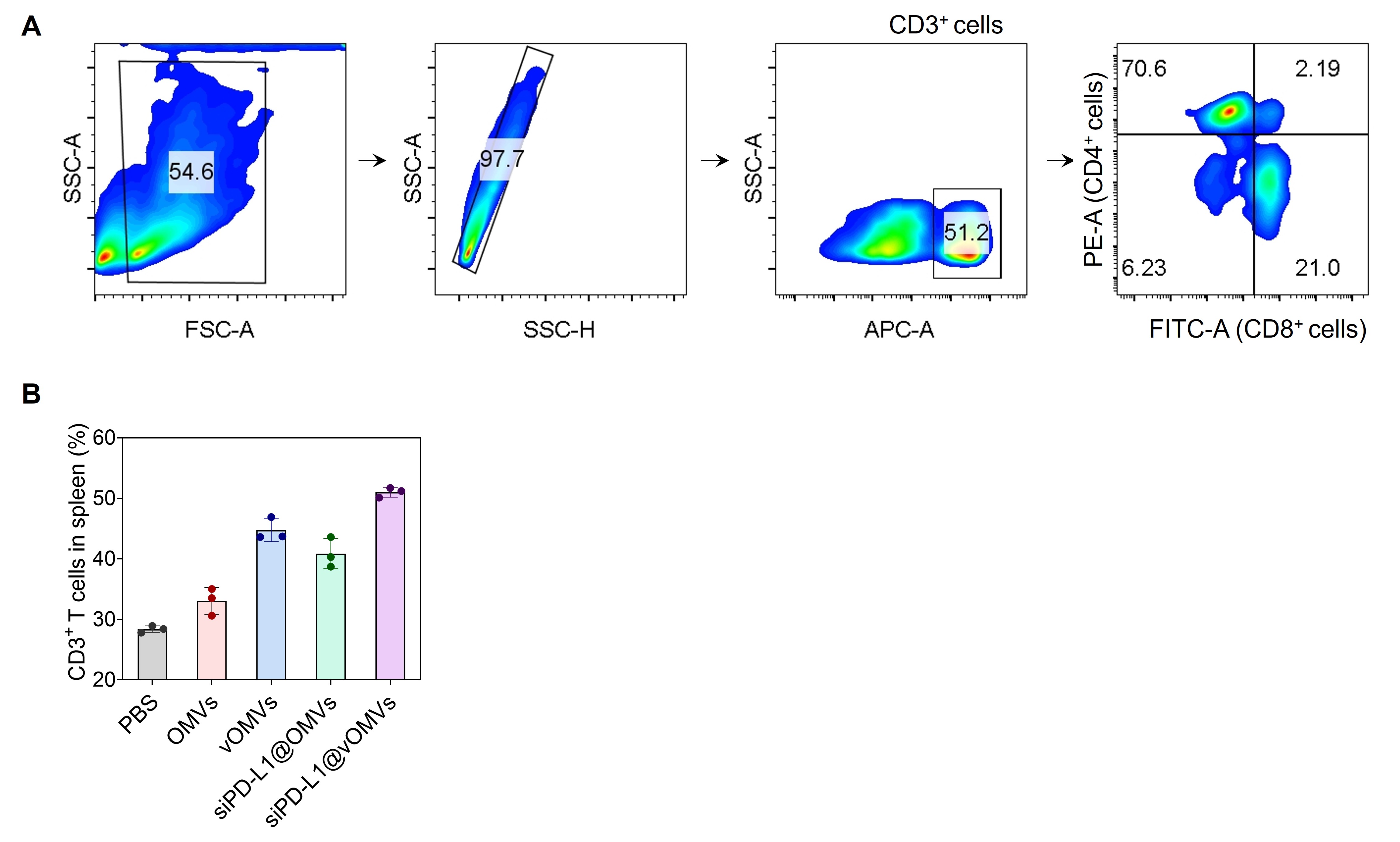
**

**Figure S24. A**) Gating strategy for flow cytometry analysis of CD4^+^, CD8^+^ T cells gated on CD3^+^ cells in spleens. **B**) Percentages of CD3^+^ T cells from different treatment groups in spleens. Data are presented as mean ± s.d. (n = 3 biologically independent samples).

**
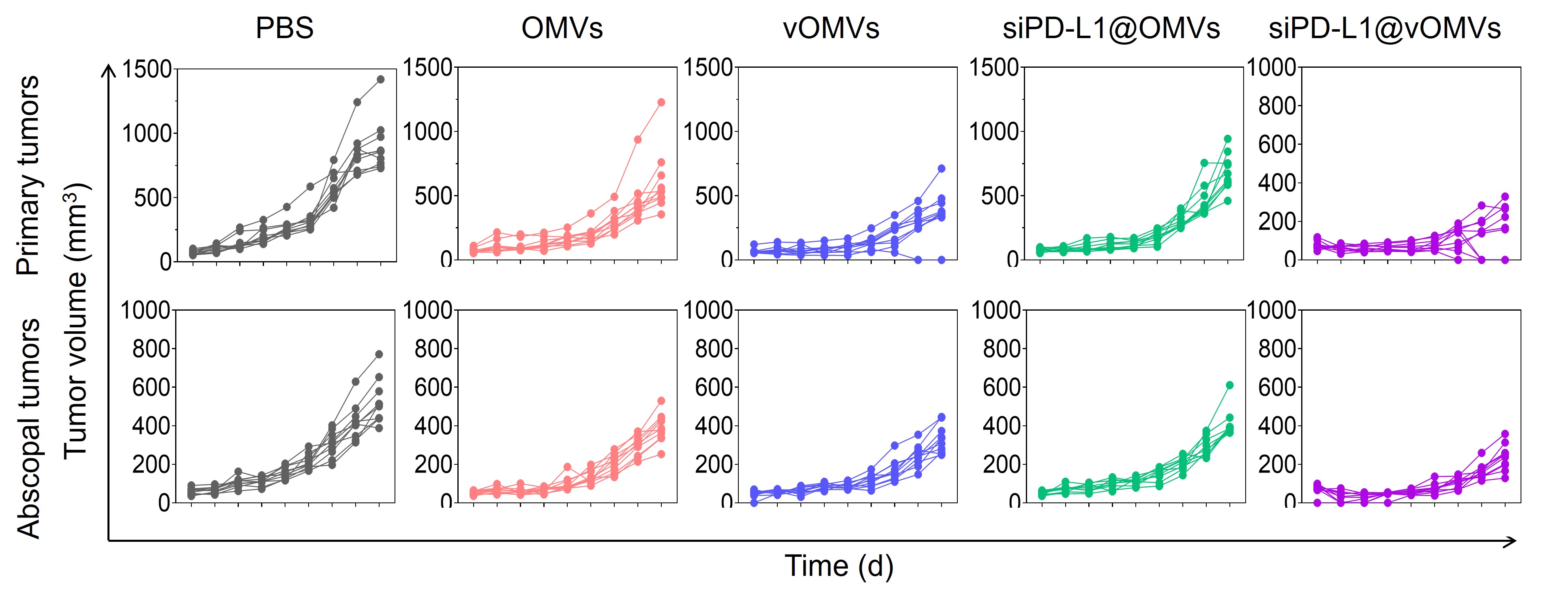
**

**Figure S25.** Treatment of CT26 tumor-bearing mice by different groups. Individual tumor growth kinetics were recorded every two days.

**
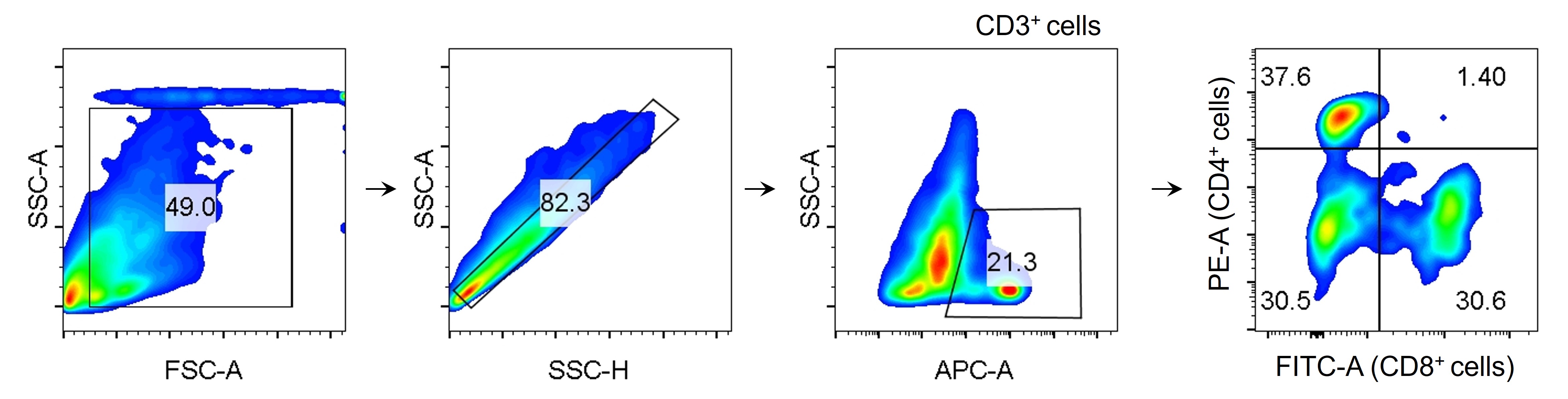
**

**Figure S26.** Gating strategy for flow cytometric studies of infiltrating T cells in primary or abscopal tumors. The tumors were isolated and analyzed on the 7th day after the final administration in the bilateral tumor model.

**
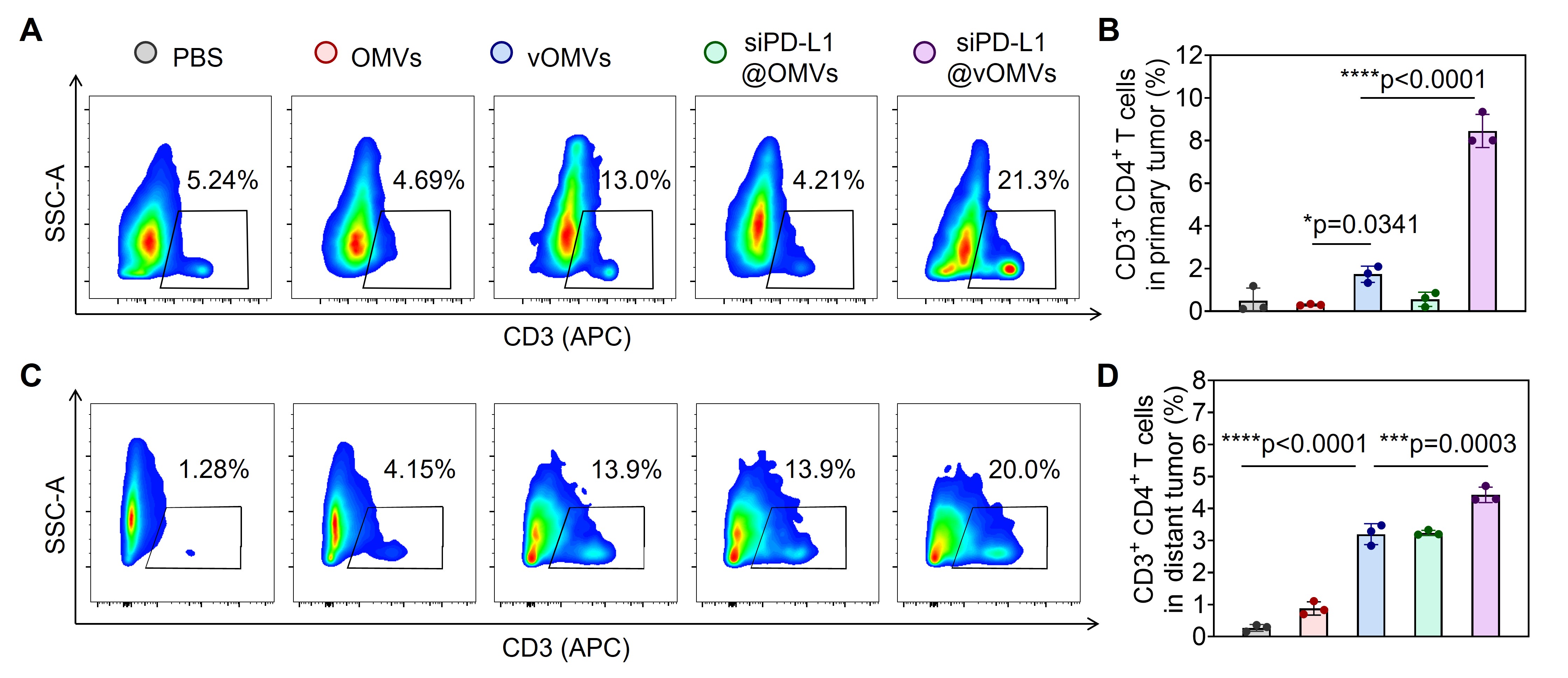
**

**Figure S27. A**) Representative flow cytometry plots showing the CD3^+^ T cells in primary tumors. **B**) Percentages of CD3^+^CD4^+^ T cells in primary tumors. **C**) Representative flow cytometry plots showing the CD3^+^ T cells in abscopal tumors. **D**) Percentages of CD3^+^CD4^+^ T cells in abscopal tumors. Data are presented as mean ± s.d. (n = 3 biologically independent samples).


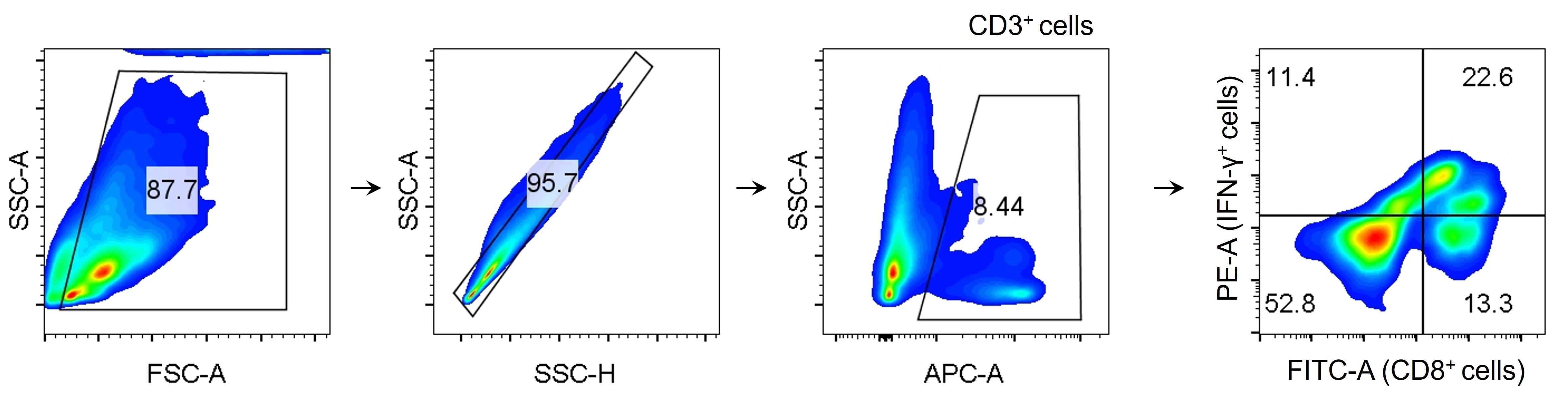


**Figure S28.** Gating strategy for flow cytometric studies of CD8^+^IFN-γ^+^ T cells (gated on CD3^+^ T cells) in splenocytes after restimulation with CT26 antigens for 24 h.


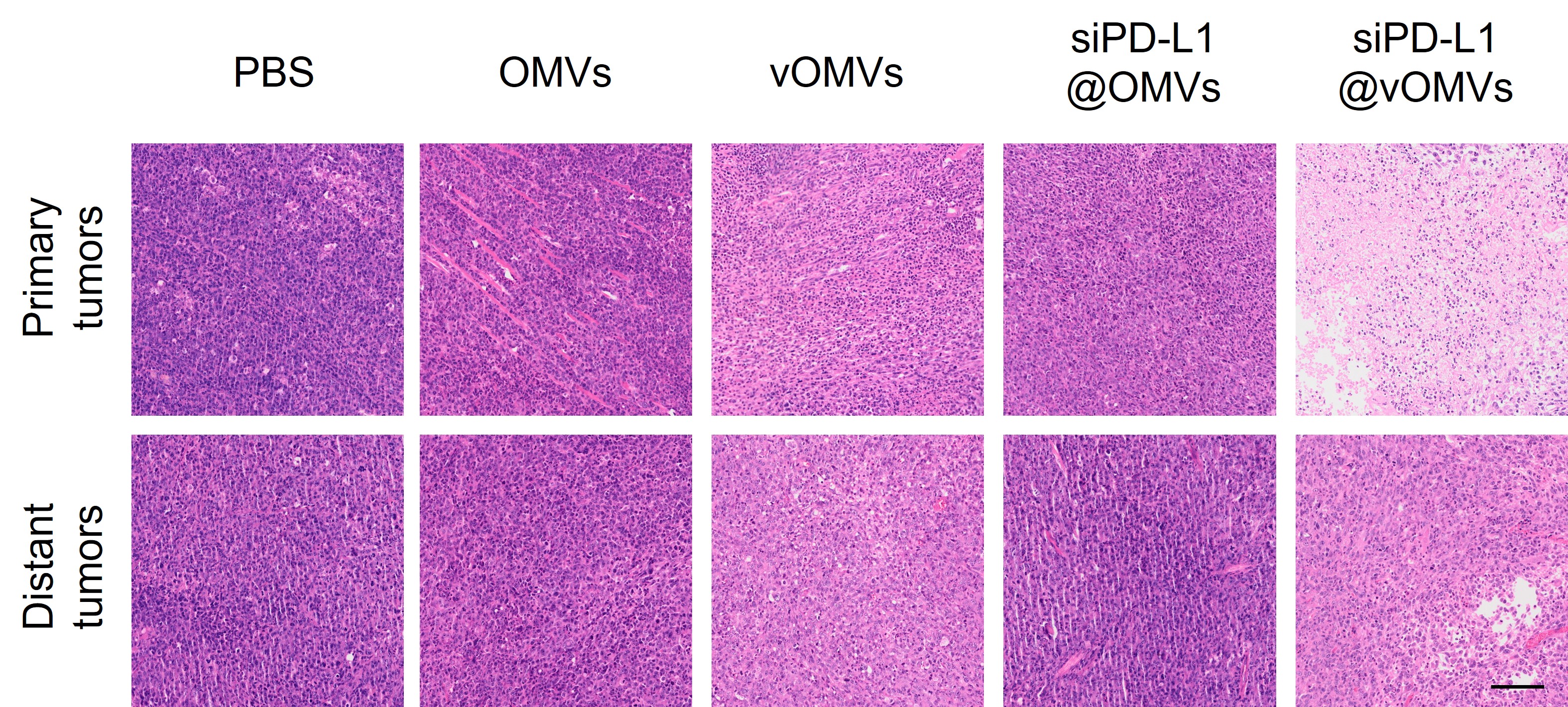


**Figure S29.** Hematoxylin and eosin (H&E) staining of primary and abscopal tumors at the end of the experiment. Scale bar: 100 μm.


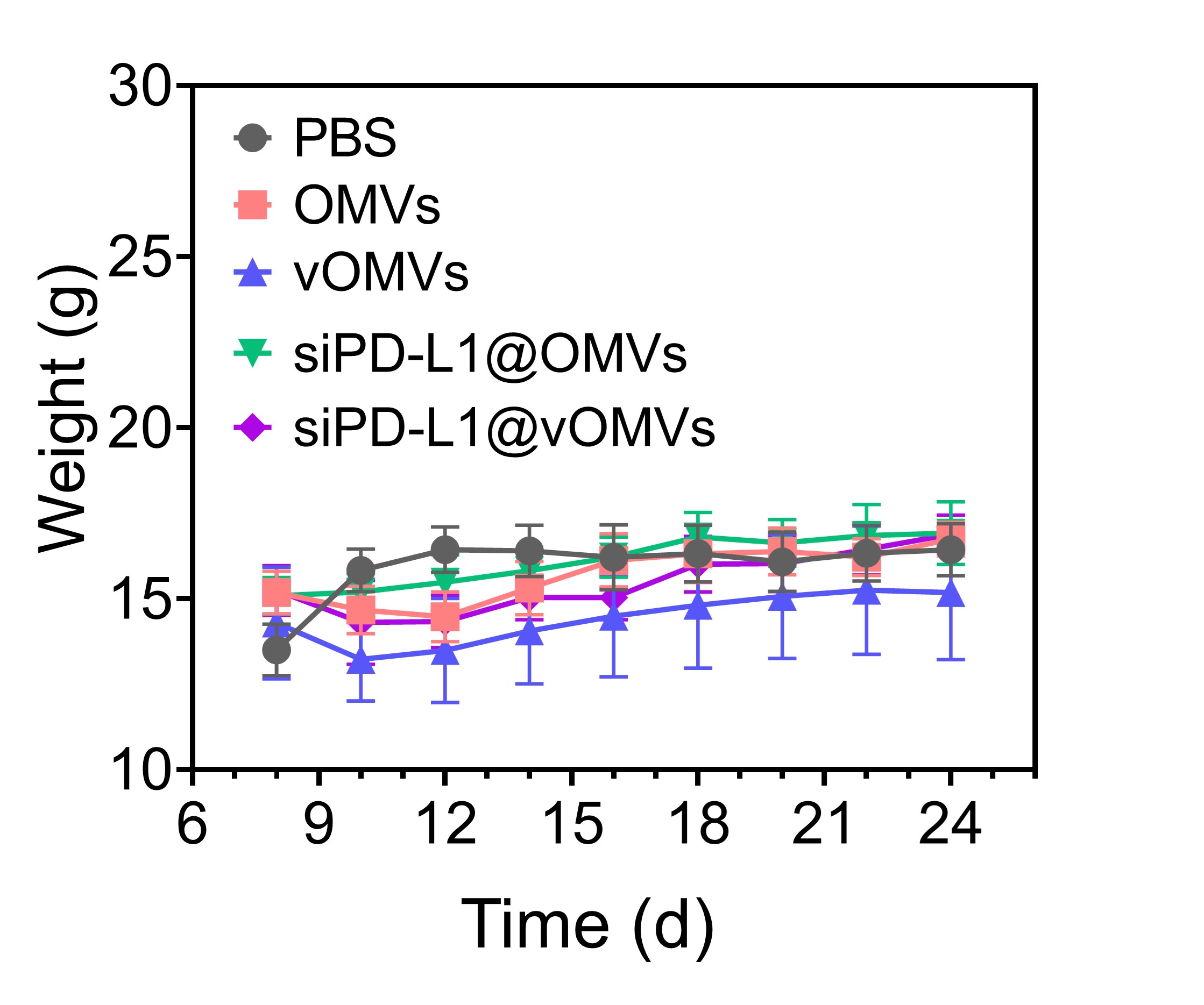


**Figure S30.** Body weight of mice during antitumor experiment against CT26. Data are presented as mean ± s.d. (n = 9 biologically independent mice).


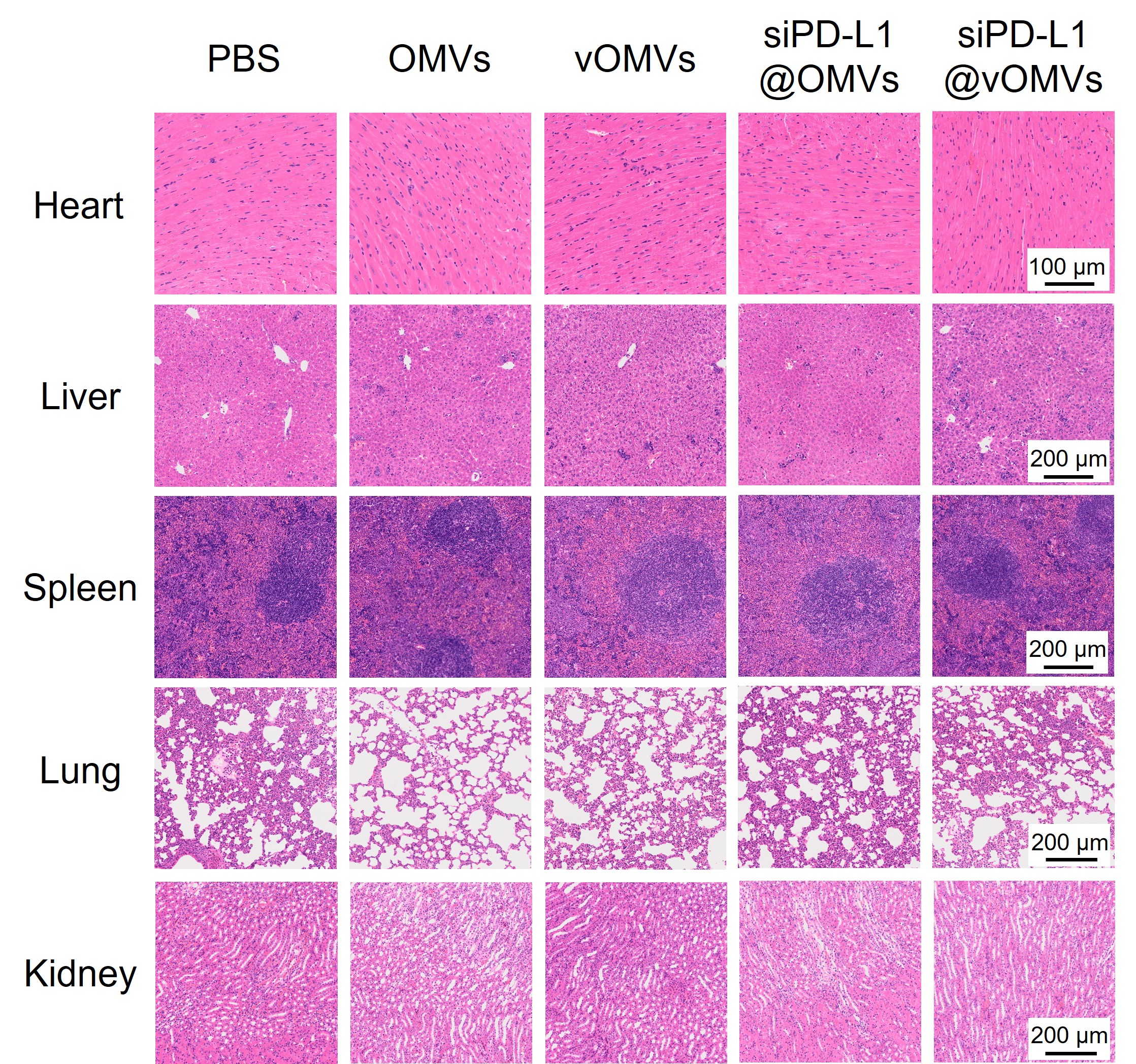


**Figure S31.** H&E staining of major organs at the endpoint of the antitumor experiment against CT26.


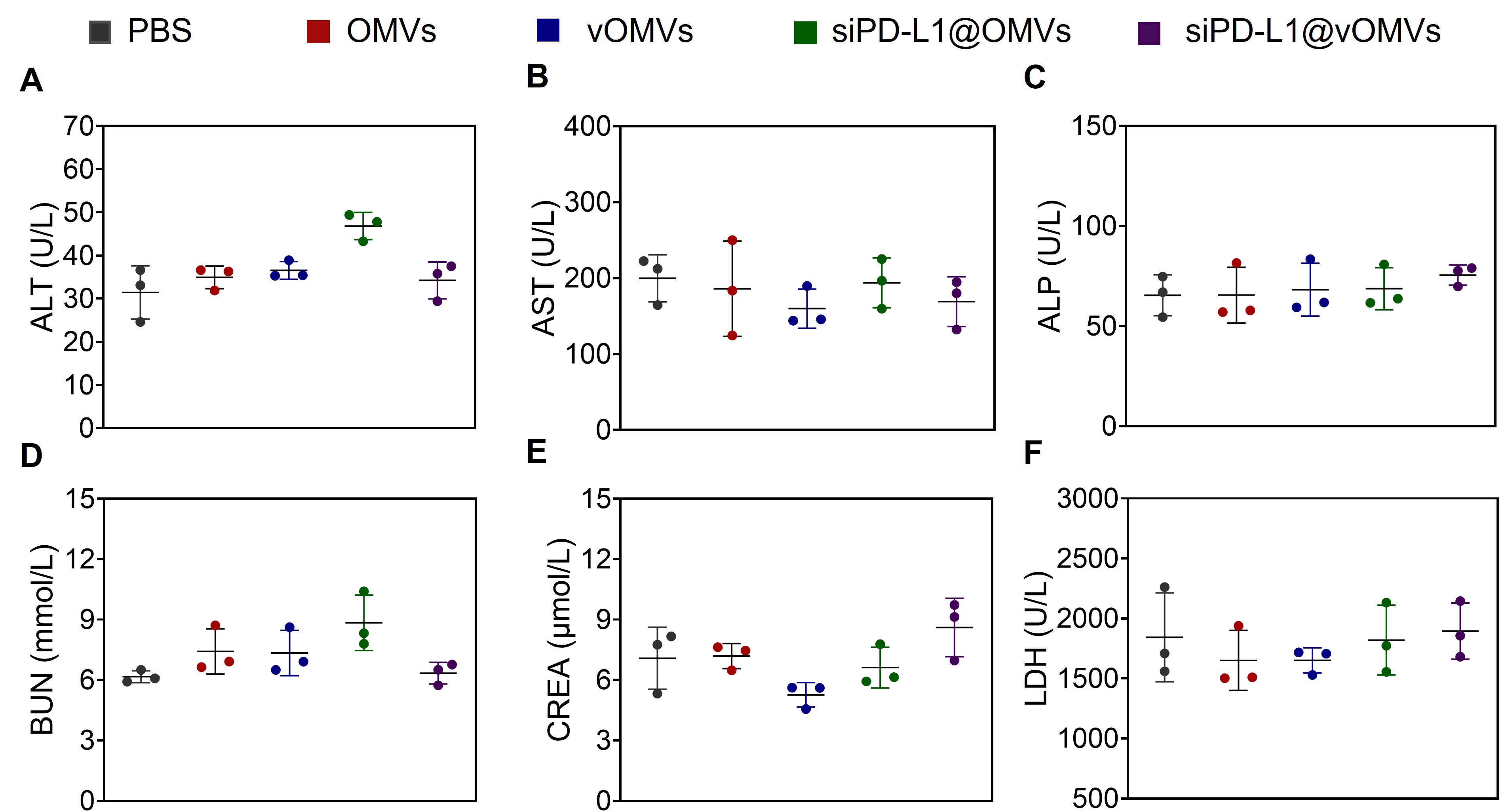


**Figure S32.** Serum biochemical indexes of hepatic function biomarkers, such as alanine transaminase (ALT) (**A**), aspartate transaminase (AST) (**B**), and alkaline phosphatase (ALP) (**C**), renal function biomarkers including urea nitrogen (BUN) (**D**) and creatinine (Crea) (**E**), and cardiac function biomarkers including lactate dehydrogenase (LDH) (**F**) in different groups at the endpoint of the antitumor experiment against CT26. Data are presented as mean ± s.d. (n = 3 biologically independent samples).


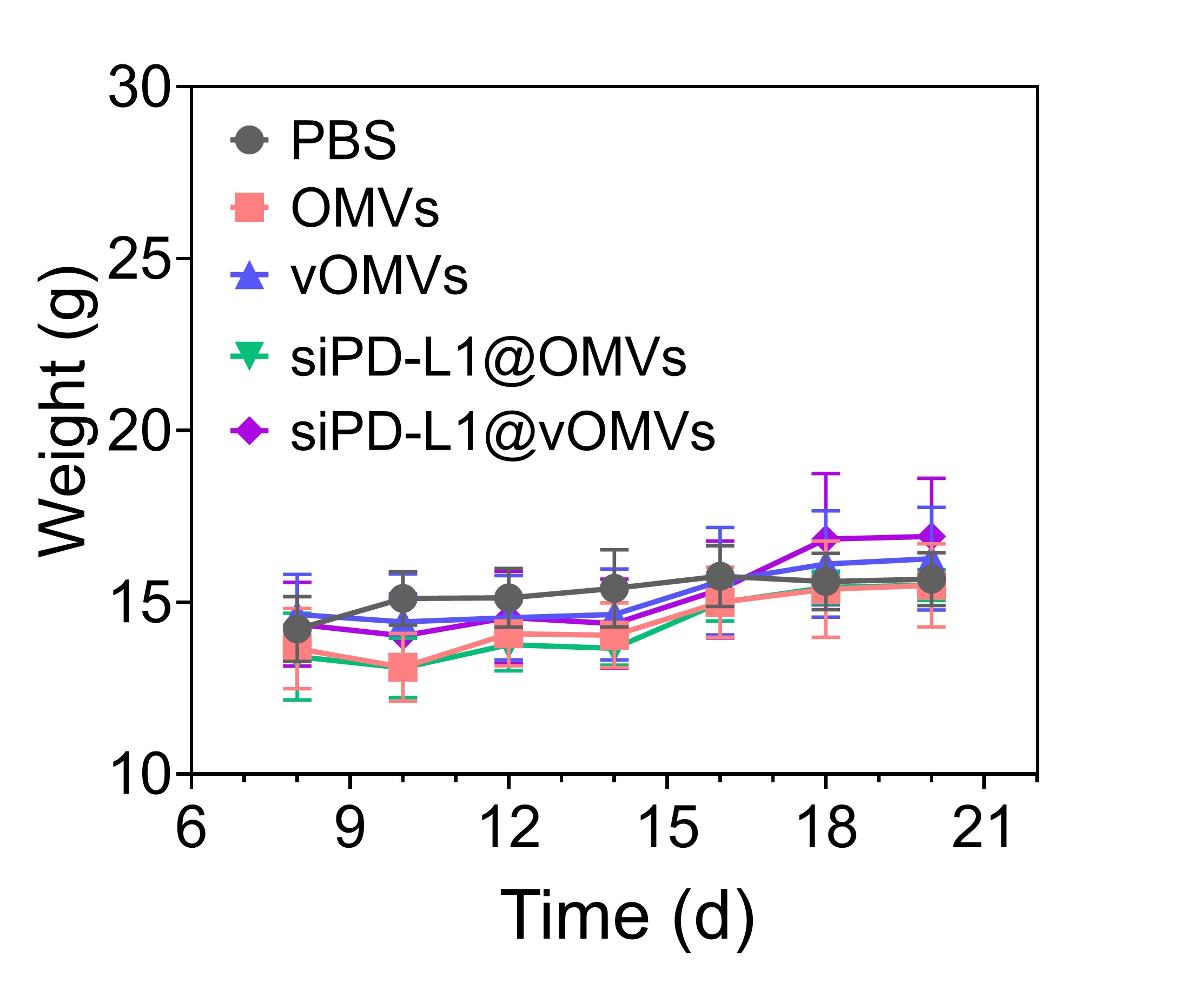


**Figure S33.** Body weight of mice during antitumor experiment against 4T1. Data are presented as mean ± s.d. (n = 9 biologically independent mice).

**
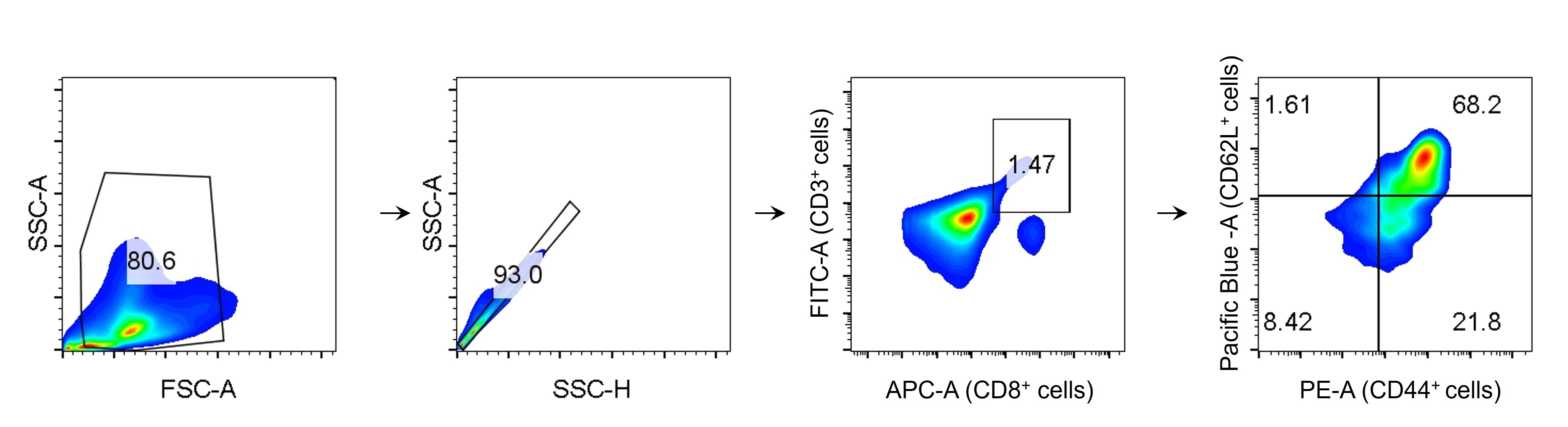
**

**Figure S34.** Gating strategy for flow cytometric studies of CD3^+^CD8^+^ T lymphocytes and CD3^+^CD8^+^CD44^+^CD62L^−^ T lymphocytes (Tem) in spleens.
